# Synthesis and photobiological applications of naphthalimide–benzothiazole conjugates: cytotoxicity and topoisomerase IIα inhibition†

**DOI:** 10.1039/d1ra04148g

**Published:** 2021-12-22

**Authors:** Iqubal Singh, Vijay Luxami, Diptiman Choudhury, Kamaldeep Paul

**Affiliations:** School of Chemistry and Biochemistry, Thapar Institute of Engineering and Technology Patiala-147001 India kpaul@thapar.edu

## Abstract

Conjugates of naphthalimide, benzothiazole, and indole moieties are synthesized that show excellent cytotoxicity against A549 (lung), MCF7 (breast), and HeLa (cervix) cancer cell lines with IC_50_ values in the range of 0.14–8.59 μM. Compounds 12 and 13 substituted with ethanolamine and propargyl groups reveal potent cytotoxicity towards A549 cancer cells with IC_50_ values of 140 and 310 nM, respectively. These compounds are further evaluated as potent inhibitors of human type IIα topoisomerase. These conjugates also reveal strong interaction towards human serum albumin (HSA) with binding constant values of 1.75 × 10^5^ M^−1^ and 1.88 × 10^5^ M^−1^, respectively, and formation of the stable complex at ground state with static quenching. Docking studies also confirm the effective interactions between conjugates and topoisomerase.

## Abstract 2

Conjugates of naphthalimide, benzothiazole, and indole moieties are synthesized that show excellent cytotoxicity against A549 (lung), MCF7 (breast), and HeLa (cervix) cancer cell lines with IC_50_ values in the range of 0.14–8.59 μM.

## Graphical abstract

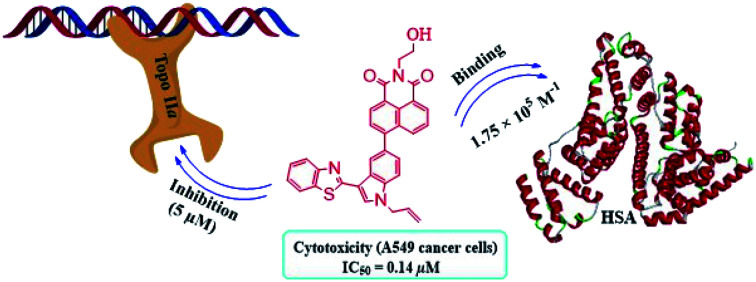

## 1. Introduction

With climate change, cancer is becoming a major problem in public health. Cancer can be described as the abnormal and uncontrolled growth of cells which spread to nearby organs *via* the circulatory system.^1,2^ A standard antitumor drug should selectively express cytotoxicity for cancer cells without harming normal cells; otherwise, it may lead to severe side effects.^3^ Existing cancer therapies, *i.e.*, radiotherapy, surgery, and chemotherapy, are becoming ineffective due to multidrug resistance. Apart from these glitches, current drugs for cancer therapies have several difficulties like poor oral bioavailability and pharmacodynamic properties.^4^ Further, different kinds of cancers can't be cured with a single anticancer drug, particularly in late-stage diseases.^5,6^ These situations motivate the researchers to develop new drug candidates with the necessary characteristics of cancer chemotherapy having the least side effects and higher therapeutic ability with minimum doses. Compounds comprising heteroatoms show a wide range of applications and are well-studied for various biological functions. Nearly about 60% of available cancer drugs contain heterocyclic moieties.^7,8^

Naphthalimide, a heterocyclic moiety, is a group of well-established antiproliferative agents, and some derivatives (amonafide, mitonafide, and UNBS5162) have reached different phases of clinical trials.^9,10^ Another heterocyclic moiety, benzothiazole-based compounds, were also reported to show extensive activities towards various biological agents^11–13^ in which this moiety exhibited excellent anticancer activity.^14–16^

**Fig. 1:**
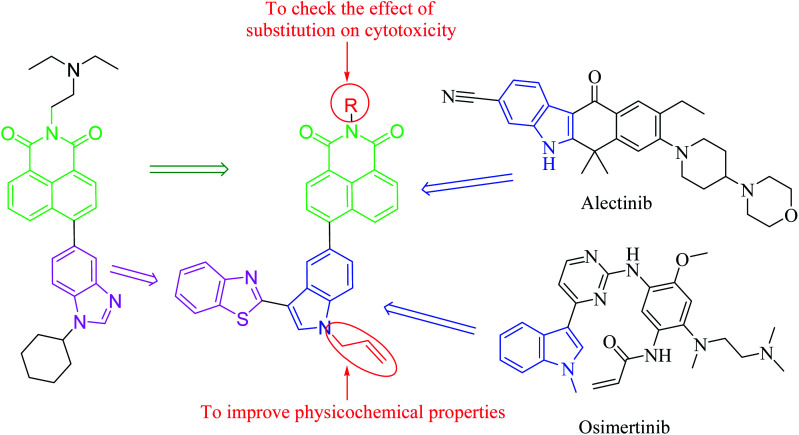
Design of naphthalimide, benzothiazole, and indole conjugates.

Previously, various hybrids of benzimidazole and naphthalimide moieties,^17^ combined without any linker, have been synthesized and were evaluated for their anticancer activities. These hybrids revealed more activity than hybrids of benzimidazole and imidazo[1,2-*a*]pyrazine,^18,19^ or naphthalimide and phenanthroimidazole.^20,21^ These hybrids also indicated higher antiproliferative activity than the clinical trial drug, amonafide. Thus, these previous results concluding the improved cytotoxicity of conjugates of naphthalimide and benzimidazole moieties. As a result of interesting biological activities with these two heterocyclic scaffolds, we decided to develop a new series of a conjugate comprising these two pharmacophoric moieties and linked together with another new pharmacophore. Therefore, we selected naphthalimide and benzothiazole (structurally related to benzimidazole) and joined these moieties using indole ring, a well-known moiety for anticancer property, as a linker (Fig. 1). Indole-based heterocyclic compounds also gained significant attention due to their wide range of pharmacological properties.^22^ Several indole derivatives, *i.e.*, alectinib and osimertinib, were found as potent anticancer agents.^23–25^ Vincristine and vinblastine are the indole-based inhibitors of tubulin protein that also increase the importance of indole scaffold in the field of cancer.^26,27^

These conjugates were synthesized possessing variable substituents, which were planned to achieve the aim of target cytotoxicity. These newly synthesized compounds were characterized by NMR and mass spectrometry and further evaluated for their cytotoxicity against three human cancer cell lines; A549 (lung), MCF7 (breast), and HeLa (cervix), and normal cell lines Hek293 (Kidney). Further, the two most active compounds were evaluated for their interactions with human type IIα topoisomerase (TOPO-IIα) and human serum albumin (HSA). The docking studies were also performed to confirm their interactions with TOPO IIα.

## 2. Chemistry

**Scheme 1:**
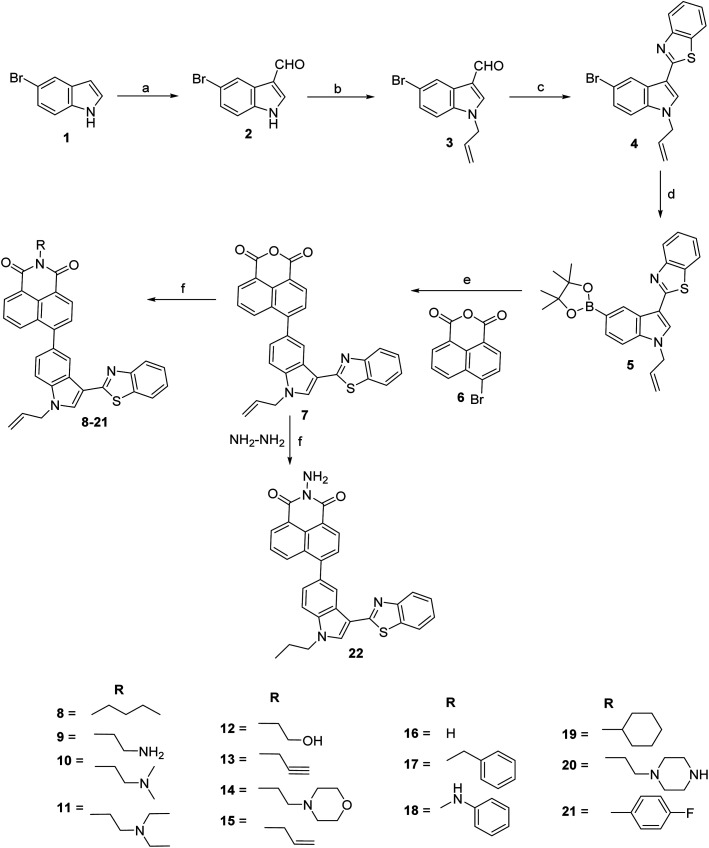
Synthesis of 6-(1-allyl-3-(benzo[*d*]thiazol-2-yl)-1*H*-indol-5-yl)-2-substituted-1*H*-benzo[*de*]isoquinoline-1,3(2*H*)-dione. Reagents and conditions: (a) POCl_3_, DMF, RT, 1 h, 94%; (b) allyl bromide, acetone-aq. NaOH, RT, 2 h, 89%; (c) 2-aminothiophenol, nitrobenzene, 80 °C, 8 h, 61%; (d) bis(pinacolato)diboron, Pd(PPh_3_)_2_Cl_2_, KOAc, dioxane, reflux, 3 h; (e) Pd(PPh_3_)_4_, K_2_CO_3_, CH_3_CN : water (9 : 1), reflux, 5 h, 55%; (f) RNH_2_, ethanol, reflux, 4–6 h, 61–79%.

Conjugates of indole, benzothiazole and naphthalimide were synthesized as depicted in Scheme 1. Commercially available 5-bromo indole 1 was subjected to react with Vilsmeier–Haack reagent (POCl_3_ and DMF) afforded 5-bromo-1*H*-indole-3-carbaldehyde 2. The appearance of singlet at *δ* 9.97 ppm in ^1^H NMR spectrum and signal at *δ* 184.9 ppm in ^13^C NMR spectrum corresponding to respective proton and carbon of carbaldehyde confirmed the formation of 2. Compound 2 was then treated with allyl bromide in acetone–NaOH (aq.) at room temperature to give brown color solid of 1-allyl-5-bromo-1*H*-indole-3-carbaldehyde 3 in 89% yield. Characteristic signals of allyl group (multiplet of one proton at *δ* 6.04–5.94 ppm representing CH of allyl, two doublets of two protons at *δ* 5.33 and 5.18 ppm representing CH_2_ of allyl and multiplet of two protons at *δ* 4.76–4.74 ppm representing N–CH_2_ of allyl) confirmed the formation of compound 3. Further, compound 3 was reacted with 2-amino thiophenol in the presence of nitrobenzene at 80 °C for 8 h, afforded reddish color solid of 2-(1-allyl-5-bromo-1*H*-indol-3-yl)benzo[*d*]thiazole 4 in 61% yield. The formation of compound 4 was confirmed by the disappearance of carbaldehyde singlet at *δ* 9.95 ppm in ^1^H NMR spectrum and the appearance of four protons of benzothiazole ring in the range of *δ* 8.62–7.24 ppm. Boronate of compound 4 was synthesized using bis(pinacolato)diboron in the presence of bis(triphenylphosphine)palladium chloride and potassium acetate in refluxing dioxane for 3 h. Compound 5 was further subjected to Suzuki–Miyaura cross-coupling with 6-bromo-1*H*,3*H*-benzo[*de*]isochromene-1,3-dione 6. Suzuki coupling afforded light yellow colour solid of 6-(1-allyl-3-(benzo[*d*]thiazol-2-yl)-1*H*-indol-5-yl)-1*H*,3*H*-benzo[*de*]isochromene-1,3-dione 7 in the presence of tetrakis(triphenylphosphine) palladium(0) and potassium carbonate in acetonitrile–water (9 : 1) mixture. ^1^H NMR spectrum of compound 7 revealed the presence of signals of naphthalimide moiety in the range of *δ* 8.72–7.32 ppm. The mass spectrum of compound 7 showed a molecular ion peak at *m*/*z* 487.1 (M^+^ + 1) that further confirmed the formation of this compound. Finally, compounds 8–21 were synthesized in 61–79% yield by condensing various amines *viz.*, allyl, butyl, propargyl, cyclohexyl, ethanolamine with compound 7 in the presence of ethanol at reflux temperature. Surprisingly, reaction with hydrazine hydrate afforded compound 22 with reduction of allyl group. The disappearance of allyl signals and the appearance of two triplets and one multiplet in ^1^H NMR confirmed the formation of compound 22. The synthesis of all these compounds was confirmed by NMR spectroscopy and mass spectrometry (Fig. S1–S55†).

## 3. Biology

### 3.1 Cytotoxicity against human cancer cells

Compounds 7–22 were tested for their cytotoxicity against A549 (lung), MCF7 (breast), and HeLa (cervix) human cancer cell lines at 1, 10, and 100 μM concentrations. All the synthesized compounds showed excellent cytotoxicity against these evaluated cancer cell lines with IC_50_ values in the range of 0.14 ± 0.01 to 8.59 ± 0.28 μM. These compounds exhibited more sensitivity towards A549 than MCF7 and HeLa cancer cells at the given concentrations. Ethanolamine substituted compound (12) revealed potent cytotoxicity against A549 cancer cells with an IC_50_ value of 0.14 ± 0.01 μM. Similarly, propargylamine substituted derivative (13) showed excellent cytotoxicity for A549 with an IC_50_ value of 0.31 ± 0.01 μM. Compound 15 substituted with allyl group found effective against three tested cancer cells with IC_50_ values of 1.89 ± 0.15 μM for A549, 0.38 ± 0.06 μM for MCF7, and 2.71 ± 0.09 μM for HeLa cancer cells. Furthermore, derivatives 18, 20, 21, and 22 showed better activity towards the A549 cell line (Table 1).

**Table 1:** Cytotoxicity of compounds (7–22) against A549, MCF7, and HeLa cancer cell lines

Compound	IC_50_ (μM)		
	A549	MCF7	HeLa
7	**0.45 ± 0.04**	6.94 ± 0.16	7.18 ± 0.28
8	1.19 ± 0.11	7.69 ± 0.20	5.52 ± 0.15
9	3.06 ± 0.13	4.17 ± 0.13	4.02 ± 0.18
10	1.02 ± 0.08	3.31 ± 0.11	3.40 ± 0.13
11	**0.81 ± 0.05**	3.57 ± 0.21	3.07 ± 0.17
12	**0.14 ± 0.01**	3.81 ± 0.19	3.76 ± 0.20
13	**0.31 ± 0.01**	3.49 ± 0.12	3.64 ± 0.19
14	1.11 ± 0.06	3.69 ± 0.09	3.93 ± 0.11
15	1.89 ± 0.15	**0.38 ± 0.06**	2.71 ± 0.09
16	1.91 ± 0.10	3.57 ± 0.08	3.61 ± 0.19
17	2.01 ± 0.09	4.39 ± 0.23	4.17 ± 0.21
18	**0.96 ± 0.12**	3.58 ± 0.14	3.96 ± 0.20
19	1.47 ± 0.12	1.65 ± 0.11	3.33 ± 0.11
20	**0.76 ± 0.04**	3.35 ± 0.16	3.45 ± 0.13
21	**0.63 ± 0.08**	4.10 ± 0.23	3.96 ± 0.16
22	**0.84 ± 0.07**	5.84 ± 0.28	8.59 ± 0.28
Etoposide	0.10 ± 0.02	0.33 ± 0.13	1.89 ± 0.19

### 3.2 Cytotoxicity against human normal cells

**Fig. 2:**
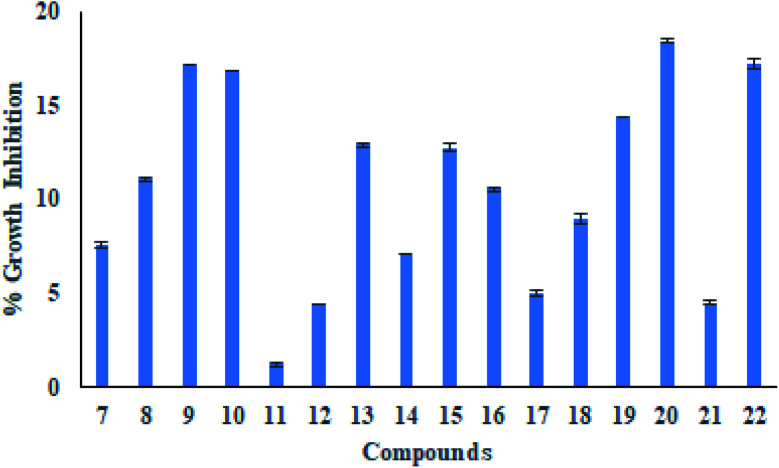
Effect of cytotoxicity of compounds (7–22) towards human normal cell line Hek293.

To evaluate the safety, the cytotoxic effect of all compounds (7–22) against Hek293 (kidney) human noncancerous cell line was also evaluated using a colorimetric assay (MTT assay) at the concentration level of 10 μM. The results showed that compound 20 exhibited maximum growth inhibition of 18%, followed by compound 9, which displayed growth inhibition of 17% (Fig. 2). These results indicated that all synthesized derivatives had low cytotoxicity towards normal mammalian cells. At the same time, conjugate 11 revealed the least toxicity with growth inhibition of about 2%, signifying that all synthesized compounds were able to kill cancer cells only selectively.

### 3.3 Topoisomerase IIα (TOPO IIα) inhibitory activity

The unwinding of DNA double helix is highly recommended at the time of replication and transcription in cell division which causes the helical tension to rise in the rest of the molecule of DNA. Topoisomerase, the nuclear enzyme, drives the topological changes and releases the tension of DNA, thus, necessary for cell proliferation.^28,29^ Especially human type II topoisomerase (topo II*a*) has been found an effective target in cancer treatment.^30,31^ Here, we have explored the ability of most active compounds 12, and 13 for their inhibition of topoisomerase IIα catalyzed relaxation activity towards supercoiled plasmid DNA, with etoposide as a positive control. In TOPO II inhibition assay, supercoiled plasmid DNA was treated with human topoisomerase II in the presence of the reference compound (etoposide) or tested compounds at concentrations of 1, 5, 10, and 50 μM. Inhibition of topoisomerase II was detected with the reference compound (etoposide), which produced a marked level of DNA double-stranded breaks corresponding to linear DNA. Fig. S56† shows that compounds 12 and 13 were significant and active inhibitors on topoisomerase II enzyme at concentrations of 5, 10, and 50 μM and partially inhibitor with 1 μM when compared with etoposide. The result indicated that introducing hydroxy and alkyne groups into the alkyl chain of naphthalimide could generate a series of derivatives with topoisomerase II inhibitory activity.

**Fig. 3:**
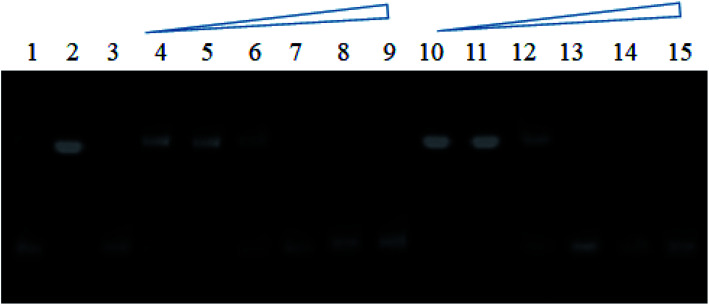
Agarose gel stained with ethidium bromide for inhibitory activity towards Topo IIα relaxation by compounds 12 and 13. Lane 1: pHOT1 plasmid DNA, lane 2: pHOT1 plasmid DNA + TOPO II, lane 3: plasmid DNA + TOPO II + etoposide (25 μM as positive control), lane 4–7 (compound 12) and lane 8–11 (compound 13): inhibition of relaxation of plasmid DNA by Topo IIα in the presence of 0.1, 0.5, 1, 1.5, 2 and 3 μM compound.

A further experiment was repeated to determine the more accurate concentration value of compounds required for topoisomerase inhibition. The concentrations 0.1, 0.5, 1, 1.5, 2, and 3 μM were taken for both compounds (Fig. 3). The results showed that inhibition of topoisomerase was started at 1 μM concentration, and complete inhibition was acquired at 1.5 μM concentration.

### 3.4 Human serum albumin (HSA) interaction studies

HSA exhibits hydrophobic and hydrophilic properties of its amino acids, which causes this protein to interact with amphiphilic molecules. HSA can bind reversibly to various drugs to increase solubility, decrease toxicity, and protect the bound ligands against oxidation in plasma. Given its exceptional abilities, HSA is commonly chosen as a target in drug–protein interaction for understanding the pharmacokinetics and pharmacological effects of drugs.

#### 3.4.1 UV-vis absorption studies

**Fig. 4:**
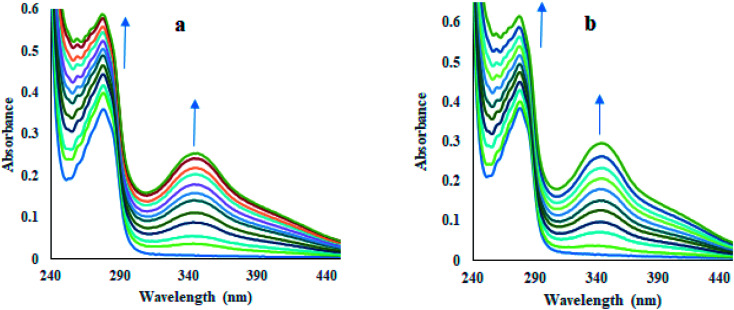
UV-visible absorption of HSA in the presence of increasing concentrations of compounds 12 (a) and 13 (b) in phosphate buffer (pH 7.4) at 298 K.

To check the interactions between HSA and compounds, UV-vis absorption spectra of HSA (10 μM) were recorded in the absence and incremental additions of compounds 12 and 13 (7 μM). Noteworthy, enhancement in absorbance of HSA at 280 nm (57% for compound 12 and 51% for compound 13) on increasing concentrations of compounds indicated perturbations in the microenvironment of protein's chromophores as a result of interaction between HSA and compounds along with the development of new band at 348 nm (Fig. 4). Increasing the concentration of compounds also produces an enhancement in absorbance value at 348 nm. The binding constants for the compound–HSA system were calculated by Benesi–Hildebrand eqn (2)^32^ as 1.75 × 10^5^ M^−1^ for compound 12 and 1.88 × 10^5^ M^−1^ for compound 13 (Fig. S57†). These strong binding affinities are favorable for the adequate transportation of these compounds to their target sites.

#### 3.4.2 Fluorescence spectra and quenching mechanism

**Fig. 5:**
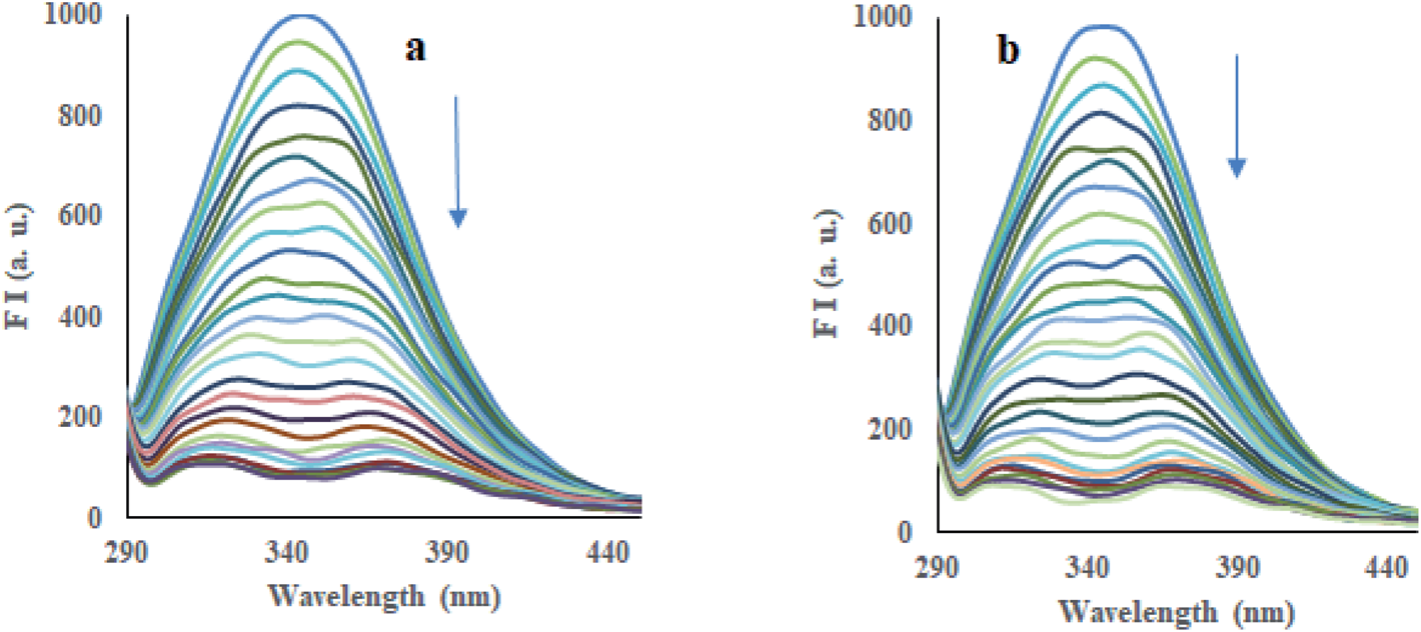
Emission spectra of HSA (*λ*_ex_ = 330 nm) in the presence of increasing concentrations of compounds 12 (a) and 13 (b) in phosphate buffer (pH 7.4) at 298 K.

HSA shows its intrinsic fluorescence mainly produced by aromatic fluorophores, *i.e.*, tryptophan (Trp) and tyrosine (Tyr) amino acid residues.^33^ The binding of compounds to HSA can affect the fluorescence of these fluorophores.^34–36^ The emission spectrum of HSA (10 μM) exhibited an emission band at 347 nm, using 280 nm as excitation wavelength in phosphate buffer (pH 7.4) at 298 K, 308 K, and 318 K, as tryptophan (Trp-214) amino acid residue located at subdomain IIA of protein.^37^ Incremental addition of compounds 12 and 13 (33 μM) led to good quenching (90–95%) of emission of HSA at 347 nm (Fig. 5, S58, and S59†), which indeed indicated the binding of compounds to HSA. Fluorescence quenching data have been analyzed using the Stern–Volmer equation eqn (3)^38^ at three different temperatures, *i.e.*, 298 K, 303 K, and 318 K, where Stern–Volmer plots were obtained (Fig. S60 and S61†). Good linearity of plots was observed having a correlation coefficient (*R*) ≥ 0.9396 throughout all the experiments. The values of the Stern–Volmer quenching constant (*K*_SV_), a measure of fluorescence quenching efficiency of the analyte, were calculated from these plots and are reported in Table 2. The fluorescence quenching mechanism, whether dynamic or static quenching, can be investigated using the dependency of *K*_SV_ values on temperature. A decrease in quenching constant is predictable with increasing temperature for static quenching, whereas the opposite trend is found in dynamic quenching.^39,40^Table 2 exhibited that the values of *K*_SV_ were promisingly decreased with increasing temperature, hence, representing the static quenching. Thus, the detected quenching in fluorescence of HSA on the addition of compounds 12 and 13 appears as a result of complex formation between compound and HSA. Moreover, the values of bimolecular quenching rate constant (*K*_q_) for compounds 12 (10.46 × 10^14^ M^−1^ s^−1^, 5.19 × 10^14^ M^−1^ s^−1^, 1.27 × 10^14^ M^−1^ s^−1^) and 13 (2.58 × 10^14^ M^−1^ s^−1^, 2.38 × 10^14^ M^−1^ s^−1^, 0.30 × 10^14^ M^−1^ s^−1^) were calculated at 298 K, 308 K and 318 K, where the values of the compound–HSA system were found considerably higher than the reported value of maximum dynamic quenching constant (2 × 10^10^ M^−1^ s^−1^) for the binding of fluorophore and quenchers in the bimolecular complex.^41^ Thus, in the present study, the static quenching phenomenon has occurred in the quenching process for represented HSA–compound systems.

**Table 2:** Interaction parameters of HSA with compounds 12 and 13 at three different temperatures (298 K, 308 K, and 318 K)

Comp.	*T* (K)	*K*_SV_ (10^6^ M^−1^)	*K*_q_ (10^14^ M^−1^ s^−1^)	*R* a	*K*_b_ (10^6^ M^−1^)	*n*	*R* a
12	298	10.46	10.46	0.9601	7.34	1.32	0.9920
	308	5.19	5.19	0.9710	1.52	1.17	0.9655
	318	1.27	1.27	0.9488	0.84	1.30	0.9763
13	298	2.58	2.58	0.9549	1.51	1.17	0.9629
	308	2.38	2.38	0.9396	0.36	1.07	0.9401
	318	0.30	0.30	0.9774	0.15	1.00	0.9669

a *R* is the correlation coefficient.

#### 3.4.3 Binding affinity

Fig. S62 and S63† display double logarithmic plots obtained from modified Stern–Volmer eqn (4)^42^ for binding of compounds 12 and 13 to HSA at different temperatures, *i.e.*, 298 K, 308 K, and 318 K. The values of binding constants (*K*_b_) for the interaction with HSA were calculated for compounds 12 (7.34 × 10^6^ M^−1^, 1.52 × 10^6^ M^−1^ and 0.84 × 10^6^ M^−1^) and 13 (1.51 × 10^6^ M^−1^, 0.36 × 10^6^ M^−1^ and 0.15 × 10^6^ M^−1^) at three different temperatures (Table 2). *K*_b_ values for the compound–HSA binding were obtained in the range of 7.34–0.15 × 10^6^ M^−1^, which directed the strong binding affinity between compounds and HSA. A decreasing trend of values of *K*_b_ for the compound–HSA system has been depicted with increasing temperature as a result of degradation of the compound–HSA complex at higher temperatures.

#### 3.4.4 Interaction forces

The thermodynamic process has been evaluated to get insight into the dependency of temperature on binding constant for compound–HSA interaction. Thermodynamic parameters, *viz.*, change in enthalpy (Δ*H*), change in entropy (Δ*S*), and binding free energy (Δ*G*) for the compound–HSA system, is essential to estimate the forces prevailing in binding interaction. The values of Δ*H* and Δ*S* were obtained from van't Hoff eqn (5), whereas the values of Δ*G* were obtained from eqn (6) at three different temperatures. The values of Δ*H* (−21.32 kJ M^−1^ for compound 12 and −21.41 kJ M^−1^ for compound 13) and Δ*S* (−40.44 J M^−1^ K^−1^ for compound 12 and −43.76 J M^−1^ K^−1^ for compound 13) were determined from the slope and intercept of the van't Hoff plot, respectively (Fig. S64†) (Table 3). Several non-covalent forces, like van der Waals interactions, hydrogen bonds, electrostatic interactions, and hydrophobic interactions, are identified to stabilize the ligand–protein complex.^43,44^ The nature of these binding forces can be predicted by the sign and magnitude of the values of Δ*S* and Δ*H*.^45^ The negative sign of Δ*H* value showed the exothermic nature of the formation of the compound–HSA complex. The negative value of Δ*S* is evidenced by the involvement of hydrogen bondings and van der Waal forces. Moreover, the negative value of Δ*G* indicated that the binding interactions are spontaneous at all temperatures and decreases with increasing temperature.

**Table 3:** Thermodynamic parameters of HSA binding with compounds 12 and 13 at three different temperatures (298 K, 308 K, and 318 K)

Comp.	*T* (K)	Δ*H*, kJ M^−1^	Δ*S*, J M^−1^ K^−1^	Δ*G*, kJ M^−1^	*R* a
12	298	−21.32	−40.44	−9.27	0.9591
	308			−8.87	
	318			−8.46	
13	298	−21.41	−43.76	−8.37	0.9827
	308			−7.93	
	318			−7.49	

a *R* is the correlation coefficient.

#### 3.4.5 Excited-state fluorescence lifetime study

A fluorescence lifetime experiment was performed to discover the quenching mechanism of compounds 12 and 13 for interactions with HSA. The decay lifetime (*τ*) generally remains unaltered in ground state quenching while it gets changed in excited state quenching.^46,47^ The fluorescence lifetime spectrum of HSA (7 μM) was performed upon increasing concentrations of compounds 12 and 13 (0–70 μM) (Fig. S65†). The results revealed that additions of compounds 12 and 13 to HSA did not produce any significant alterations in the decay time of free HSA (Table 4). Therefore, it can conclude that the fluorescence quenching of HSA by compounds 12 and 13 was characterized as a static quenching and thus formed a stable complex with HSA at the ground state.

**Table 4:** Fluorescence lifetime decay of HSA in free form and presence of compounds^a^

Comp.	Conc. (μM)	*τ*_1_ (ns)	*α*_1_	*τ*_2_ (ns)	*α*_2_	*τ*_3_ (ns)	*α*_3_	*τ* (ns)	*χ* ^2^
12	0	3.40	0.43	7.39	0.21	0.85	0.36	3.30	1.165
	7	3.29	0.42	7.32	0.20	0.80	0.38	3.13	1.132
	35	3.37	0.41	7.34	0.18	0.86	0.40	3.09	1.104
	70	3.14	0.39	7.03	0.20	0.80	0.41	2.97	1.153
13	0	3.25	0.42	7.16	0.24	0.81	0.34	3.38	1.188
	7	3.19	0.41	7.17	0.23	0.84	0.36	3.25	1.122
	35	3.12	0.39	6.97	0.24	0.85	0.37	3.19	1.176
	70	3.36	0.39	7.25	0.19	0.86	0.41	3.08	1.275

a *χ* ^2^ is the goodness of fit.

#### 3.4.6 Synchronous fluorescence spectroscopy

**Fig. 6:**
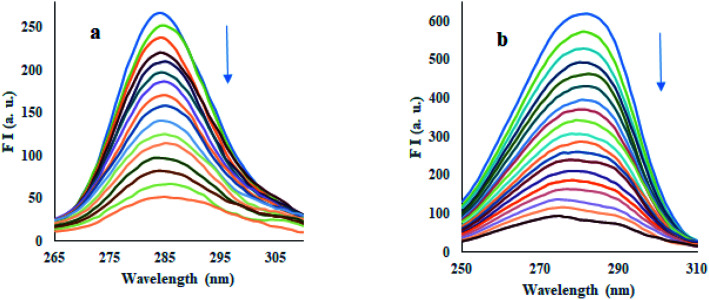
Synchronous fluorescence spectra of HSA upon addition of compound 12 at (a) Δ*λ* = 15 nm and (b) Δ*λ* = 60 nm.

Synchronous fluorescence spectroscopy (SFS) is commonly used to evaluate the effect of ligand binding on the microenvironment of protein and its amino acids. It is usually observed that a change in polarity of the chromophore of protein causes deviation in the excitation wavelength of a particular amino acid residue.^48,49^ This deviation can provide information about the change in confirmation of HSA on the binding of ligand. The SFS of HSA was noted using a fixed difference in wavelengths (Δ*λ*) of 15 nm and 60 nm of synchronized recording of excitation and emission monochromators, which are characteristics of tyrosine and tryptophan amino acid residues, respectively.^50,51^ As depicted in Fig. 6 and S66,† both compounds 12 and 13 were significantly quenched the fluorescence intensities of HSA at Δ*λ* = 15 nm and 60 nm. Results revealed that both tyrosine and tryptophan residues equally contribute to the quenching of HSA fluorescence. The addition of compounds 12 and 13 produced a slight blue shift of about 4 nm at Δ*λ* = 15 nm, corresponding to a tyrosine residue.

In contrast, a slight redshift of about 6 nm was observed at Δ*λ* = 60 nm, corresponding to tryptophan residue. The results indicated that these compounds altered the HSA confirmation so that a decrease in polarity near tyrosine residue while increasing the polarity around tryptophan residue was depicted. Therefore, tyrosine and tryptophan residues were positioned at higher and lower hydrophobic environments, respectively.

#### 3.4.7 Energy transfer between HSA and compounds

**Fig. 7:**
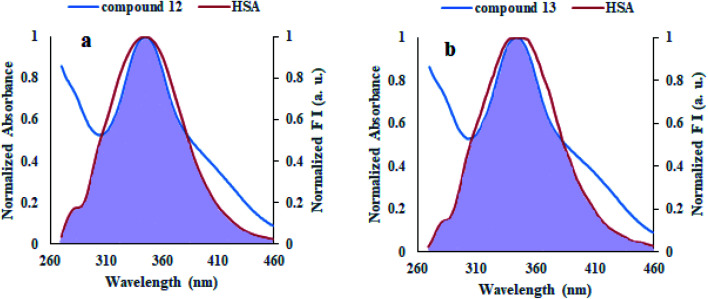
Overlap of a normalized absorption spectrum of compounds 12 (a) and 13 (b) (blue) with the normalized emission spectrum of HSA (red).

Förster Resonance Energy Transfer (FRET) concept could be used to determine the distance between the binding site of HSA and the fluorophore present in the protein.^52^Fig. 7 shows the overlapping of an emission spectrum of HSA and the absorption spectrum of compound 12 or 13. Eqn (8)–(10) are used to calculate the values of overlap integral of an emission spectrum of donor with the absorption spectrum of the acceptor (*J*), the critical distance (50% efficiency of energy transfer, *R*_0_), efficiency of energy transfer (*E*), and the distance among donor and acceptor (*r*). The calculated values for compound 12 were found to be *J* = 1.04 × 10^−18^ cm^3^ L mol^−1^, *R*_0_ = 0.592 nm, *E* = 0.92, and *r* = 0.393 nm, whereas compound 13 gave the values *J* = 1.039 × 10^−18^ cm^3^ L mol^−1^, *R*_0_ = 0.591 nm, *E* = 0.93, *r* = 0.377 nm. The calculated distance between donor and acceptor for compounds 12 and 13 was found less than 8 nm, and both compounds follow 0.5*R*_0_ < *r* < 1.5*R*_0_. These results showed that energy transfer from HSA to compound 12 or 13 has occurred with high probability.^53^

## 4. Molecular docking

**Fig. 8:**
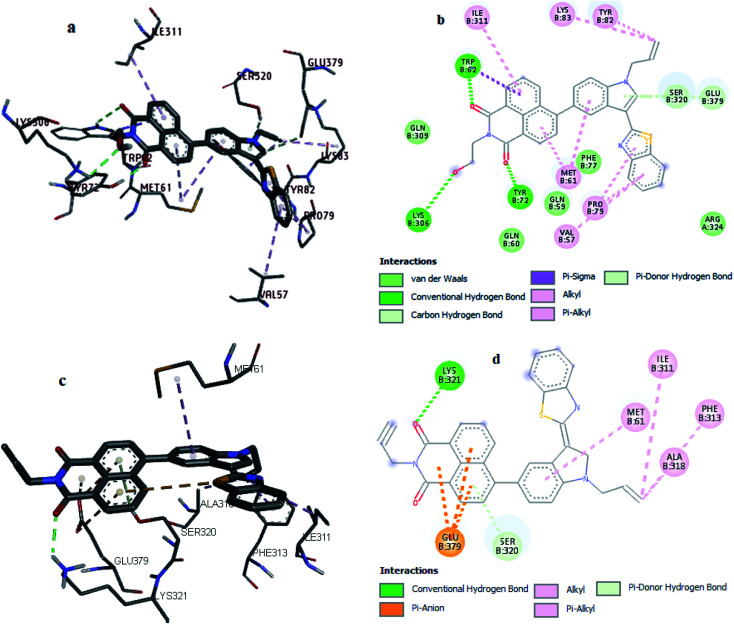
3D and 2D representation of interactions of compound 12 (a and b) and 13 (c and d) with human type II*a* topoisomerase.

To visual insight the interactions of human topoisomerase II*a* (PDB: 1ZXM)^54^ with compounds 8–22 and etoposide, molecular docking was performed by the AutoDock program (Tables S1–S4†).^55^ The docking studies of topoisomerase with compounds provided the best confirmation of the binding having minimum binding energy −10.7 and −11.0 kcal mol^−1^ for compounds 12 and 13, respectively (Table S2†). The results of docking studies demonstrated that these compounds embedded into the pocket at chain B of topoisomerase, which is enclosed by Val57, Gln59, Gln60, Met61, Trp62, Tyr72, Phe77, Pro79, Tyr82, Lys83, Lys306, Gln309, Ile311, Phe313, Ala318, Ser320, Lys321 and Glu379 amino acid residues (Fig. 8). Docking of compound 12–topoisomerase system revealed hydrogen bonding between the hydrogen atom of Trp62 and Tyr72 residues of topoisomerase and the oxygen atom of the amide group of compound 12 with bond lengths of 2.64 Å and 1.85 Å, respectively. The oxygen atom of the hydroxyl group of compound 12 exhibited hydrogen bonding with the hydrogen atom of Lys306 residue of topoisomerase with 2.78 Å bond length. Naphthalimide, benzothiazole, and indole rings of compound 12 presented hydrophobic interactions through π–alkyl with Met61, Pro79, Val57, and Ile311 residues topoisomerase. Allyl group of compound 12 interacts through π–alkyl and alkyl–alkyl interactions with Tyr82 and Lys83 residues of topoisomerase, respectively. Whereas compound 13 revealed hydrogen bonding between the hydrogen atom of Lys321 residue of topoisomerase and the oxygen atom of the amide group of compound 13 with a bond length of 2.24 Å. Hydrophobic interactions are shown by Ile311 and Ala318 residues through alkyl–alkyl and Phe313 residue through π–alkyl interactions with the allyl group of compound 13. Moreover, the benzimidazole ring of compound 13 showed hydrophobic interaction with Met61 residue through π–alkyl interactions. Naphthalimide ring of compound 13 displayed electrostatic interaction with Glu379 residue of topoisomerase through π–anion interaction (Table 5).

**Table 5:** Interactions between human type II*a* DNA topoisomerase and compounds (12 and 13) gained from molecular docking

Comp.	Interaction	Category of interaction	Type of interaction
12	B:Trp62:HE1[H-donor⋯H-acceptor]compound 12:O	H-Bonding	Hydrogen bond
	B:Tyr72:HH[H-donor⋯H-acceptor]compound 12:O	H-Bonding	Hydrogen bond
	B:Lys306:HZ1[H-donor⋯H-acceptor]compound 12:O	H-Bonding	Hydrogen bond
	Compound 12[π-orbitals⋯alkyl]B:Met61	Hydrophobic	π–Alkyl
	Compound 12[π-orbitals⋯alkyl]B:Pro79	Hydrophobic	π–Alkyl
	Compound 12[π-orbitals⋯alkyl]B:Val57	Hydrophobic	π–Alkyl
	Compound 12[π-orbitals⋯alkyl]B:Ile311	Hydrophobic	π–Alkyl
	B:Tyr82[π-orbitals⋯alkyl]compound 12	Hydrophobic	π–Alkyl
	Compound 12[alkyl⋯alkyl]B:Lys83	Hydrophobic	Alkyl–alkyl
	B:Trp62:CD1[π-orbitals⋯σ-orbitals]compound 12	Hydrophobic	π–σ
13	B:Lys321:HZ1[H-donor⋯H-acceptor]compound 13:O	H-bonding	Hydrogen bond
	Compound 13[π-orbitals⋯alkyl]B:Met61	Hydrophobic	π–Alkyl
	B:Phe313[π-orbitals⋯alkyl]compound 13	Hydrophobic	π–Alkyl
	B:Ala318[alkyl⋯alkyl]compound 13	Hydrophobic	Alkyl–alkyl
	Compound 13[alkyl⋯alkyl]B:Ile311	Hydrophobic	Alkyl–alkyl
	B:Glu379:OE1[negative⋯π-orbitals]compound 13	Electrostatic	π–Anion
	B:Glu379:OE2[negative⋯π-orbitals]compound 13	Electrostatic	π–Anion

## 5. Conclusion

A series of conjugates with three biological active pharmacophores of naphthalimide, benzothiazole, and indole substituted with different aliphatic and aromatic amines (7–22) has been synthesized in moderate to good yields. Compounds 7–22 were tested for their cytotoxicity against A549 (lung), MCF7 (breast), and HeLa (cervix) human cancer cells that showed cytotoxicity in the range of IC_50_ values of 0.14–8.59 μM, indicated that compounds showed excellent activity towards these cancer cell lines. Cytotoxicity studies towards normal mammalian kidney cells Hek293 designated that all synthesized compounds could selectively kill cancer cells only. Results of topoisomerase inhibition assays with compounds 12 and 13 revealed that the newly synthesized derivatives exerted their antitumor activities by inhibiting human topoisomerase II activity. Moreover, strong interactions of compounds 12 and 13 with HSA were found with binding constants 1.75 × 10^5^ M^−1^ and 1.88 × 10^5^ M^−1^, respectively, through static quenching, the process for the formation of compound–HSA complex. The thermodynamic parameters showed that the binding interactions are spontaneous and exothermic, as well as the involvement of hydrogen bonding and van der Waals forces. The results of energy transfer studies between HSA and compounds revealed a high probability of transfer energy with values of efficiency of energy transfer (*E*) of 0.9 and 0.93 for compounds 12 and 13, respectively. Molecular docking between topoisomerase and compounds gave the best confirmation of binding with minimum binding energies of −10.7 and −11.0 kcal mol^−1^ for compounds 12 and 13, respectively.

## 6. Experimental section

### 6.1 Chemistry

All commercially available compounds (Aldrich, Merck, Spectrochem, *etc.*) were used without further purification. All the recorded melting points were uncorrected and measured in open capillaries. ^1^H NMR and ^13^C NMR characterizations have been performed on Jeol ECS 400 NMR spectrometer, which was operated at 400 MHz for ^1^H nuclei and 100 MHz for ^13^C nuclei, taking CDCl_3_ and DMSO-*d*_6_ as solvents. Chemical shifts are reported in parts per million (ppm), and TMS was used as an internal reference. Coupling constants (*J*) were reported in hertz (Hz). The synthesized compounds were analyzed with mass spectra using Water Micromass-Q-T of Micro. Elemental analysis was done with Thermo Scientific (Flash 2000) analyzer. Purification of synthesized compounds was done through column chromatography with the help of silica gel having a mesh size of 60–120 using hexane/ethyl acetate and chloroform/methanol in various polarity systems. All absorption spectra of compounds and HSA were recorded on Shimadzu UV-visible spectrophotometer. All emission spectra were performed on a fluorescence spectrophotometer of Agilent technology. Time-resolved fluorescence spectra were recorded using DeltaFlex Modular Fluorescence Lifetime Spectro Fluorometer (HORIBA Scientific). For MTT assay, 96 well plates were read using a Bio-Tek ELISA plate reader. Supercoiled plasmid DNA (Inspiralis limited, Norwich UK) was supplied by labex corporation, New Delhi, India.

#### 5-Bromo-1*H*-indole-3-carbaldehyde (2)

5-Bromoindole 1 (2 g, 10.30 mmol) was taken in a dry round bottom flask (RBF). Vilsmeier–Haack reagent [*N*,*N*-dimethyl formamide (DMF) and phosphorous oxychloride (POCl_3_)] was added to RBF dropwise at room temperature. The reaction mixture was stirred for 1 h at room temperature, and the progress of the reaction was monitored with the help of thin-layer chromatography (TLC). The reaction mixture was poured onto crushed ice and neutralized the solution with 20% NaOH aqueous. Precipitated solid was filtered and washed thoroughly with cold water. Air-dried the precipitate to obtain the desired white solid product in 2.15 g; 94% yield; *R*_f_ 0.5 (20% ethyl acetate in hexane); mp 203–205 °C (lit.^56^ 202–204 °C); ^1^H NMR (CDCl_3_ + DMSO-*d*_6_, 400 MHz): *δ* (ppm) 9.97 (s, 1H, CHO), 8.41 (s, 1H, NH), 7.85 (d, *J* = 3.16 Hz, 1H, ArH), 7.38–7.35 (m, 3H, ArH); ^13^C NMR (CDCl_3_ + DMSO-*d*_6_, 100 MHz): *δ* (ppm) 184.9 (*C*HO), 137.3, 135.9, 126.6, 126.1, 124.2, 118.3, 115.9, 113.6 (Ar*C*).

#### 1-Allyl-5-bromo-1*H*-indole-3-carbaldehyde (3)

To synthesize 1-allyl-5-bromo-1*H*-indole-3-carbaldehyde (3), an oven dried RBF was charged with 10 ml of acetone–20% NaOH aqueous (4 : 1) and 5-bromo-1*H*-indole-3-carbaldehyde (2) (2 g, 9.00 mmol). Allyl bromide (1.6 g, 13.50 mmol) was added to reaction mixture and stirred at room temperature for 2 h. Progress of reaction was monitored through TLC. Acetone was distilled off and water was added to the reaction mixture. Precipitated brown solid was filtered, washed with cold water and air dried to obtain the desired product in 2.09 g; 89% yield; *R*_f_ 0.6 (10% ethyl acetate in hexane); mp 66–69 °C; ^1^H NMR (CDCl_3_, 400 MHz): *δ* (ppm) 9.95 (s, 1H, CHO), 8.46 (d, *J* = 1.84 Hz, 1H, ArH), 7.70 (s, 1H, ArH), 7.41 (dd, ^2^*J* = 8.72 Hz, ^3^*J* = 1.88 Hz, 1H, ArH), 7.22 (d *J* = 8.72 Hz, 1H, ArH), 6.04–5.94 (m, 1H, allyl-CH), 5.33 (d, *J* = 10.40 Hz, 1H, allyl-CH_2_), 5.18 (d, *J* = 16.88 Hz, 1H, allyl-CH_2_), 4.76–4.74 (m, 2H, allyl-CH_2_); ^13^C NMR (CDCl_3_, 100 MHz): *δ* (ppm) 184.4 (*C*HO), 138.9, 135.9, 131.4, 127.1, 126.9, 124.8, 119.4, 117.7, 116.7, 111.8 (Ar*C*), 49.8 (allyl-*C*H_2_); MS (ESI): *m*/*z* 264 (M^+^ + 1).

#### 2-(1-Allyl-5-bromo-1*H*-indol-3-yl)benzo[*d*]thiazole (4)

An oven dried RBF was charged with 1-allyl-5-bromo-1*H*-indole-3-carbaldehyde (3) (1 g, 3.78 mmol) followed by addition of nitrobenzene (2 ml). 2-Aminothiophenol (475 mg, 3.78 mmol) was added and stirred the reaction at 80 °C for 8 h. 30 ml of hexane was added to the reaction mixture and precipitate was filtered and washed with hexane. Crude product was purified by column chromatography using 5% ethyl acetate in hexane as eluents. 850 mg; reddish solid; 61% yield; *R*_f_ 0.3 (10% ethyl acetate in hexane); mp 111–114 °C; ^1^H NMR (CDCl_3_, 400 MHz): *δ* (ppm) 8.62 (d, *J* = 1.84 Hz, 1H, ArH), 8.05 (d, *J* = 8.20 Hz, 1H, ArH), 7.88 (d, *J* = 8.64 Hz, 1H, ArH), 7.85 (s, 1H, ArH), 7.49–7.45 (m, 1H, ArH), 7.42 (dd, ^2^*J* = 8.72 Hz, ^3^*J* = 1.92 Hz, 1H, ArH), 7.36–7.32 (m, 1H, ArH), 7.26–7.24 (m, 1H, ArH), 6.07–5.97 (m, 1H, allyl-CH), 5.32 (d, *J* = 10.08 Hz, 1H, allyl-CH_2_), 5.20 (d, *J* = 16.96 Hz, 1H, allyl-CH_2_), 4.79–4.77 (m, 2H, allyl-CH_2_); ^13^C NMR (CDCl_3_, 100 MHz): *δ* (ppm) 162.0, 153.9, 135.6, 133.7, 132.0, 130.5, 127.3, 126.2, 124.4, 124.2, 122.3, 121.3, 118.8, 115.3, 111.7, 111.1 (Ar*C*), 49.5 (allyl-*C*H_2_); MS (ESI): *m*/*z* 371 (M^+^ + 1).

#### 2-(1-Allyl-5-(4,4,5,5-tetramethyl-1,3,2-dioxaborolan-2-yl)-1*H*-indol-3-yl)benzo[*d*]thiazole (5)

2-(1-Allyl-5-bromo-1*H*-indol-3-yl)benzo[*d*]thiazole (4) (1 g, 2.71 mmol), bis(pinacolato)diboron (1.03 g, 4.06 mmol), Pd(PPh_3_)_2_Cl_2_ (1.0 mol%) and KOAc (265 mg, 2.71 mmol) were taken in a dry RBF and 20 ml of dioxane was added. The reaction was allowed to reflux for 3 h. Reaction progression was observed by TLC [*R*_f_ 0.4 (10% ethyl acetate in hexane)]. The solvent was distilled off under reduced pressure followed by 100 ml of water was added to it. Extraction was done with chloroform (3 × 50 ml) and dried the extract over anhydrous sodium sulphate. Chloroform was distilled off to get the crude product. The crude product was further used without any purification.

#### 6-(1-Allyl-3-(benzo[*d*]thiazol-2-yl)-1*H*-indol-5-yl)-1*H*,3*H*-benzo[*de*]isochromene-1,3-dione: (7)

2-(1-Allyl-5-(4,4,5,5-tetramethyl-1,3,2-dioxaborolan-2-yl)-1*H*-indol-3-yl)benzo[*d*]thiazole (5) (500 mg, 1.20 mmol), 6-bromo-1*H*,3*H*-benzo[*de*]isochromene-1,3-dione (6) (330 mg, 1.20 mmol), Pd(PPh_3_)_4_ (1.0 mol%) and K_2_CO_3_ (165 mg, 1.20 mmol) were added to the RBF followed by addition of CH_3_CN : water (9 : 1) (5 ml). Reaction was refluxed for 5 h and monitored through TLC. Solvent was distilled off under reduced pressure and 100 ml of water was added. Chloroform (3 × 30 ml) was used for extraction and dried over anhydrous sodium sulphate followed by distilled off the solvent to get crude product. Column chromatography was used for purification using 30% ethyl acetate in chloroform as eluents. 486 mg; Yellow solid; 55% yield; *R*_f_ 0.3 (25% ethyl acetate in chloroform); mp 255–258 °C; ^1^H NMR (CDCl_3_, 400 MHz): *δ* (ppm) 8.72–8.63 (m, 2H, ArH), 8.52–8.46 (m, 1H, ArH), 8.01–7.94 (m, 2H, ArH), 7.92–7.83 (m, 2H, ArH), 7.78–7.64 (m, 2H, ArH), 7.61–7.56 (m, 1H, ArH), 7.49–7.43 (m, 2H, ArH), 7.36–7.32 (m, 1H, ArH), 6.20–6.08 (m, 1H, allyl-CH), 5.43–5.28 (m, 2H, allyl-CH_2_), 4.94 (d, *J* = 4.92 Hz, 2H, allyl-CH_2_); ^13^C NMR (CDCl_3_, 100 MHz): *δ* (ppm) 162.0, 161.0, 160.8, 153.8, 149.6, 136.8, 134.7, 133.3, 133.0, 132.1, 132.0, 131.6, 130.9, 130.8, 130.7, 128.8, 127.1, 126.1, 126.0, 124.9, 124.4, 123.1, 122.2, 121.2, 118.9, 117.1, 111.8, 110.5 (Ar*C*), 49.5 (allyl-*C*H_2_); MS (ESI): *m*/*z* 487.1 (M^+^ + 1); anal. calcd for C_30_H_18_N_2_O_3_S: C, 74.06; H, 3.73; N, 5.76; S, 6.59; found C, 74.11; H, 3.71; N, 5.70; S, 6.68.

#### 6-(1-Allyl-3-(benzo[*d*]thiazol-2-yl)-1*H*-indol-5-yl)-2-substituted-1*H*-benzo[*de*]isoquinoline-1,3(2*H*)-dione (8–22)

For the synthesis of 6-(1-allyl-3-(benzo[*d*]thiazol-2-yl)-1*H*-indol-5-yl)-2-substituted-1*H*-benzo[*de*]isoquinoline-1,3(2*H*)-dione (8–22), respective amine was treated with 6-(1-allyl-3-(benzo[*d*]thiazol-2-yl)-1*H*-indol-5-yl)-1*H*,3*H*-benzo[*de*]isochromene-1,3-dione (7) in refluxing ethanol for 4–6 h. Thin-layer chromatography was used to check the progress of the reaction. After completion of the reaction, the solvent was distilled off under reduced pressure, and 50 ml of water was added. Filtered the residue and washed with water. Purification was done with column chromatography using ethyl acetate and chloroform as eluents.

#### 6-(1-Allyl-3-(benzo[*d*]thiazol-2-yl)-1*H*-indol-5-yl)-2-butyl-1*H*-benzo[*de*]isoquinoline-1,3(2*H*)-dione (8)

Light yellow solid; 76% yield; *R*_f_ 0.4 (20% ethyl acetate in chloroform); mp 277–280 °C; ^1^H NMR (CDCl_3_, 400 MHz): *δ* (ppm) 8.68–8.62 (m, 3H, ArH), 8.38 (d, *J* = 8.36 Hz, 1H, ArH), 7.97 (s, 1H, ArH), 7.96 (d, *J* = 8.08 Hz, 1H, ArH), 7.85 (t, *J* = 6.00 Hz, 2H, ArH), 7.69 (t, *J* = 7.64 Hz, 1H, ArH), 7.56 (d, *J* = 8.44 Hz, 1H, ArH), 7.46–7.40 (m, 2H, ArH), 7.32 (t, *J* = 7.40 Hz, 1H, ArH), 6.16–6.07 (m, 1H, allyl-CH), 5.39 (d, *J* = 10.16 Hz, 1H, allyl-CH_2_), 5.32 (d, *J* = 17.16 Hz, 1H, allyl-CH_2_), 4.91 (d, *J* = 5.32 Hz, 2H, allyl-CH_2_), 4.26 (t, *J* = 7.48 Hz, 2H, butyl-CH_2_), 1.80–1.73 (m, 2H, butyl-CH_2_), 1.53–1.44 (m, 2H, butyl-CH_2_), 1.02 (t, *J* = 7.32 Hz, 3H, butyl-CH_3_); ^13^C NMR (CDCl_3_, 100 MHz): *δ* (ppm) 164.5, 164.3, 162.1, 153.8, 147.8, 136.7, 133.6, 133.1, 132.4, 132.1, 131.1, 130.8, 130.6, 130.5, 128.3, 126.7, 126.0, 125.9, 125.1, 124.3, 122.9, 122.8, 122.2, 121.3, 121.2, 119.1, 118.8, 110.3 (Ar*C*), 49.5 (allyl-*C*H_2_), 40.2 (butyl-*C*H_2_), 30.2 (butyl-*C*H_2_), 20.4 (butyl-*C*H_2_), 13.9 (butyl-*C*H_3_); MS (ESI): *m*/*z* 542.2 (M^+^ + 1); anal. calcd for C_34_H_27_N_3_O_2_S: C, 75.39; H, 5.02; N, 7.76; S, 5.92; found C, 75.44; H, 5.09; N, 7.61; S, 5.98.

#### 6-(1-Allyl-3-(benzo[*d*]thiazol-2-yl)-1*H*-indol-5-yl)-2-(2-aminoethyl)-1*H*-benzo[*de*]isoquinoline-1,3(2*H*)-dione (9)

Yellow solid; 67% yield; *R*_f_ 0.3 (20% ethyl acetate in chloroform); mp 271–274 °C; ^1^H NMR (CDCl_3_, 400 MHz): *δ* (ppm) 8.68–8.61 (m, 3H, ArH), 8.38 (d, *J* = 8.44 Hz, 1H, ArH), 7.98 (s, 1H, ArH), 7.96 (d, *J* = 7.96 Hz, 1H, ArH), 7.85 (t, *J* = 8.44 Hz, 2H, ArH), 7.69 (t, *J* = 8.00 Hz, 1H, ArH), 7.56 (d, *J* = 8.52 Hz, 1H, ArH), 7.46–7.41 (m, 2H, ArH), 7.33 (t, *J* = 7.76 Hz, 1H, ArH), 6.17–6.07 (m, 1H, allyl-CH), 5.39 (d, *J* = 10.16 Hz, 1H, allyl-CH_2_), 5.32 (d, *J* = 17.04 Hz, 1H, allyl-CH_2_), 4.92 (d, *J* = 5.44 Hz, 2H, allyl-CH_2_), 4.41 (t, *J* = 6.96 Hz, 2H, ethyl-CH_2_), 2.72 (t, *J* = 7.20 Hz, 2H, ethyl-CH_2_); ^13^C NMR (CDCl_3_, 100 MHz): *δ* (ppm) 164.5, 164.3, 162.1, 153.8, 148.0, 136.7, 133.6, 133.2, 132.4, 132.1, 131.1, 130.9, 130.6, 130.5, 128.7, 128.3, 126.6, 126.0, 125.9, 125.1, 124.3, 123.0, 122.7, 122.2, 121.2, 118.8, 111.7, 110.3 (Ar*C*), 56.9 (ethyl-*C*H_2_), 49.5 (allyl-*C*H_2_), 38.1 (ethyl-*C*H_2_); anal. calcd for C_34_H_27_N_3_O_2_S: C, 72.71; H, 4.58; N, 10.60; S, 6.06; found C, 72.67; H, 4.51; N, 10.71; S, 6.12.

#### 6-(1-Allyl-3-(benzo[*d*]thiazol-2-yl)-1*H*-indol-5-yl)-2-(2-(dimethylamino)ethyl)-1*H*-benzo[*de*] isoquinoline-1,3(2*H*)-dione (10)

Yellow solid; 73% yield; *R*_f_ 0.3 (30% ethyl acetate in chloroform); mp 274–277 °C; ^1^H NMR (CDCl_3_, 400 MHz): *δ* (ppm) 8.67–8.61 (m, 3H, ArH), 8.37 (d, *J* = 8.36 Hz, 1H, ArH), 7.98 (s, 1H, ArH), 7.96 (d, *J* = 8.04 Hz, 1H, ArH), 7.85–7.80 (m, 2H, ArH), 7.68 (t, *J* = 7.80 Hz, 1H, ArH), 7.55 (d, *J* = 8.44 Hz, 1H, ArH), 7.45–7.40 (m, 2H, ArH), 7.32 (t, *J* = 7.40 Hz, 1H, ArH), 6.17–6.07 (m, 1H, allyl-CH), 5.39 (d, *J* = 10.28 Hz, 1H, allyl-CH_2_), 5.31 (d, *J* = 17.00 Hz, 1H, allyl-CH_2_), 4.91 (d, *J* = 5.36 Hz, 2H, allyl-CH_2_), 4.40 (t, *J* = 7.00 Hz, 2H, ethyl-CH_2_), 2.72 (t, *J* = 7.20 Hz, 2H, ethyl-CH_2_), 2.39 (s, 6H, N–CH_3_); ^13^C NMR (CDCl_3_, 100 MHz): *δ* (ppm) 164.5, 164.3, 162.1, 153.8, 148.0, 136.7, 133.6, 133.2, 132.4, 132.1, 131.1, 130.9, 130.6, 130.5, 128.7, 128.3, 126.6, 126.0, 125.9, 125.1, 124.3, 123.0, 122.7, 122.2, 121.2, 118.8, 111.7, 110.3 (Ar*C*), 56.9 (ethyl-*C*H_2_), 49.5 (allyl-*C*H_2_), 45.7 (N–*C*H_3_), 38.1 (ethyl-*C*H_2_); MS (ESI): *m*/*z* 557.2 (M^+^ + 1); anal. calcd for C_34_H_28_N_4_O_2_S: C, 73.36; H, 5.07; N, 10.06; S, 5.76; found C, 73.31; H, 5.01; N, 10.01; S, 5.81.

#### 6-(1-Allyl-3-(benzo[*d*]thiazol-2-yl)-1*H*-indol-5-yl)-2-(2-(diethylamino)ethyl)-1*H*-benzo[*de*] isoquinoline-1,3(2*H*)-dione (11)

Light yellow solid; 67% yield; *R*_f_ 0.3 (30% ethyl acetate in chloroform); mp 281–284 °C; ^1^H NMR (CDCl_3_, 400 MHz): *δ* (ppm) 8.68–8.61 (m, 3H, ArH), 8.38 (d, *J* = 8.40 Hz, 1H, ArH), 7.97 (s, 1H, ArH), 7.96 (d, *J* = 8.20 Hz, 1H, ArH), 7.85–7.81 (m, 2H, ArH), 7.69 (t, *J* = 7.68 Hz, 1H, ArH), 7.55 (d, *J* = 8.48 Hz, 1H, ArH), 7.45–7.40 (m, 2H, ArH), 7.33–7.29 (m, 1H, ArH), 6.16–6.06 (m, 1H, allyl-CH), 5.39 (d, *J* = 10.28 Hz, 1H, allyl-CH_2_), 5.31 (d, *J* = 17.16 Hz, 1H, allyl-CH_2_), 4.91 (d, *J* = 5.16 Hz, 2H, allyl-CH_2_), 4.37 (t, *J* = 7.48 Hz, 2H, ethyl-CH_2_), 2.86 (t, *J* = 7.36 Hz, 2H, ethyl-CH_2_), 2.73 (q, *J* = 7.04 Hz, 4H, ethyl-CH_2_), 1.15 (t, *J* = 7.08 Hz, 6H, ethyl-CH_3_); ^13^C NMR (CDCl_3_, 100 MHz): *δ* (ppm) 164.4, 164.2, 162.1, 153.8, 147.9, 136.7, 133.6, 133.2, 132.4, 132.1, 131.1, 130.8, 130.6, 130.5, 130.1, 128.7, 128.3, 126.7, 126.0, 125.9, 125.1, 124.3, 122.9, 122.2, 121.2, 118.8, 118.5, 111.7, 110.3 (Ar*C*), 49.8 (ethyl-*C*H_2_), 49.5 (allyl-*C*H_2_), 47.6 (ethyl-*C*H_2_), 37.9 (ethyl-*C*H_2_), 12.2 (ethyl-CH_3_); MS (ESI): *m*/*z* 585.2 (M^+^ + 1); anal. calcd for C_36_H_32_N_4_O_2_S: C, 73.95; H, 5.52; N, 9.58; S, 5.48; found C, 73.91; H, 5.48; N, 9.64; S, 5.42.

#### 6-(1-Allyl-3-(benzo[*d*]thiazol-2-yl)-1*H*-indol-5-yl)-2-(2-hydroxyethyl)-1*H*-benzo[*de*]isoquinoline-1,3(2*H*)-dione (12)

Light yellow solid; 69% yield; *R*_f_ 0.4 (30% ethyl acetate in chloroform); mp 292–295 °C; ^1^H NMR (CDCl_3_, 400 MHz): *δ* (ppm) 8.76 (d, *J* = 7.80 Hz, 1H, ArH), 8.72 (d, *J* = 7.28 Hz, 1H, ArH), 8.64 (s, 1H, ArH), 8.47 (d, *J* = 8.60 Hz, 1H, ArH), 7.99 (s, 1H, ArH), 7.96 (d, *J* = 8.08 Hz, 1H, ArH), 7.89 (d, *J* = 7.40 Hz, 1H, ArH), 7.85 (d, *J* = 7.76 Hz, 1H, ArH), 7.75 (t, *J* = 8.08 Hz, 1H, ArH), 7.58 (d, *J* = 8.40 Hz, 1H, ArH), 7.47–7.41 (m, 2H, ArH), 7.33 (t, *J* = 7.56 Hz, 1H, ArH), 7.00 (t, *J* = 8.28 Hz, 4H, 2 × ethyl-CH_2_), 6.17–6.08 (m, 1H, allyl-CH), 5.40 (d, *J* = 10.24 Hz, 1H, allyl-CH_2_), 5.32 (d, *J* = 17.08 Hz, 1H, allyl-CH_2_), 4.92 (d, *J* = 5.36 Hz, 2H, allyl-CH_2_); ^13^C NMR (CDCl_3_, 100 MHz): *δ* (ppm) 165.3, 165.2, 162.1, 153.7, 148.3, 136.7, 133.5, 133.6, 132.2, 132.1, 131.3, 131.0, 130.7, 130.4, 128.7, 128.3, 126.6, 126.1, 125.8, 125.0, 124.3, 122.9, 122.3, 122.2, 121.2, 120.9, 118.8, 111.6, 110.4 (Ar*C*), 61.9 (ethyl-*C*H_2_), 49.5 (allyl-*C*H_2_), 42.8 (ethyl-*C*H_2_); MS (ESI): *m*/*z* 530.1 (M^+^ + 1); anal. calcd for C_32_H_23_N_3_O_3_S: C, 72.57; H, 4.38; N, 7.93; S, 6.05; found C, 72.62; H, 4.42; N, 7.89; S, 6.09.

#### 6-(1-Allyl-3-(benzo[*d*]thiazol-2-yl)-1*H*-indol-5-yl)-2-(prop-2-yn-1-yl)-1*H*-benzo[*de*]isoquinoline-1,3(2*H*)-dione (13)

Light yellow solid; 76% yield; *R*_f_ 0.5 (20% ethyl acetate in chloroform); mp 282–285 °C; ^1^H NMR (CDCl_3_, 400 MHz): *δ* (ppm) 8.69 (d, *J* = 7.56 Hz, 1H, ArH), 8.65 (d, *J* = 7.20 Hz, 1H, ArH), 8.61 (s, 1H, ArH), 8.39 (d, *J* = 8.08 Hz, 1H, ArH), 7.97 (s, 1H, ArH), 7.95 (d, *J* = 8.08 Hz, 1H, ArH), 7.84 (d, *J* = 7.88 Hz, 1H, ArH), 7.82 (d, *J* = 7.56 Hz, 1H, ArH), 7.68 (t, *J* = 8.20 Hz, 1H, ArH), 7.55 (d, *J* = 8.48 Hz, 1H, ArH), 7.45–7.39 (m, 2H, ArH), 7.32 (t, *J* = 7.20 Hz, 1H, ArH), 6.17–6.07 (m, 1H, allyl-CH), 5.39 (d, *J* = 10.32 Hz, 1H, allyl-CH_2_), 5.32 (d, *J* = 17.08 Hz, 1H, allyl-CH_2_), 5.02 (d, *J* = 2.36 Hz, 2H, propagyl-CH_2_), 4.92 (d, *J* = 5.44 Hz, 2H, allyl-CH_2_), 2.24 (t, *J* = 2.40 Hz, 1H, propagyl-CH); ^13^C NMR (CDCl_3_, 100 MHz): *δ* (ppm) 163.7, 163.5, 162.0, 153.8, 148.4, 136.7, 133.6, 132.2, 132.1, 131.4, 131.2, 130.7, 130.6, 128.7, 128.3, 126.7, 126.0, 125.9, 125.1, 124.3, 123.0, 122.3, 122.2, 121.2, 120.9, 118.8, 111.7, 110.3 (Ar*C*), 78.7 (propargyl-*C*), 70.4 (propargyl-*C*H), 49.5 (allyl-*C*H_2_), 29.4 (propargyl-*C*H_2_); MS (ESI): *m*/*z* 524.1 (M^+^ + 1); anal. calcd for C_33_H_21_N_3_O_2_S: C, 75.70; H, 4.04; N, 8.03; S, 6.12; found C, 75.68; H, 4.09; N, 8.07; S, 6.10.

#### 6-(1-Allyl-3-(benzo[*d*]thiazol-2-yl)-1*H*-indol-5-yl)-2-(2-morpholinoethyl)-1*H*-benzo[*de*]isoquinoline-1,3(2*H*)-dione (14)

Yellow solid; 72% yield; *R*_f_ 0.3 (20% ethyl acetate in chloroform); mp 285–288 °C; ^1^H NMR (CDCl_3_, 400 MHz): *δ* (ppm) 8.68–8.62 (m, 3H, ArH), 8.39 (d, *J* = 8.36 Hz, 1H, ArH), 7.98 (s, 1H, ArH), 7.96 (d, *J* = 8.00 Hz, 1H, ArH), 7.84 (d, *J* = 7.44 Hz, 2H, ArH), 7.71 (t, *J* = 7.88 Hz, 1H, ArH), 7.56 (d, *J* = 8.48 Hz, 1H, ArH), 7.49–7.40 (m, 2H, ArH), 7.32 (t, *J* = 7.40 Hz, 1H, ArH), 6.15–6.07 (m, 1H, allyl-CH), 5.39 (d, *J* = 10.40 Hz, 1H, allyl-CH_2_), 5.32 (d, *J* = 17.12 Hz, 1H, allyl-CH_2_), 4.91 (d, *J* = 5.08 Hz, 2H, allyl-CH_2_), 4.42 (t, *J* = 6.80 Hz, 2H, ethyl-CH_2_), 3.72 (t, *J* = 3.92 Hz, 4H, morph-CH_2_), 2.77 (t, *J* = 6.84 Hz, 2H, ethyl-CH_2_), 2.63 (bs, 4H, morph-CH_2_); ^13^C NMR (CDCl_3_, 100 MHz): *δ* (ppm) 164.5, 164.3, 162.1, 153.8, 148.0, 136.7, 133.6, 133.2, 132.4, 132.1, 131.1, 130.9, 130.6, 130.5, 128.7, 128.4, 126.7, 126.0, 125.9, 125.1, 124.3, 123.0, 122.7, 122.2, 121.2, 118.8, 111.7, 110.3 (Ar*C*), 67.0 (morph-*C*H_2_), 56.1 (ethyl-*C*H_2_), 53.8 (morph-*C*H_2_), 49.5 (allyl-*C*H_2_), 37.1 (ethyl-*C*H_2_); MS (ESI): *m*/*z* 599.2 (M^+^ + 1); anal. calcd for C_36_H_30_N_4_O_3_S: C, 72.22; H, 5.05; N, 9.36; S, 5.35; found C, 72.20; H, 5.11; N, 9.31; S, 5.30.

#### 2-Allyl-6-(1-allyl-3-(benzo[*d*]thiazol-2-yl)-1*H*-indol-5-yl)-1*H*-benzo[*de*]isoquinoline-1,3(2*H*)-dione (15)

Yellow solid; 79% yield; *R*_f_ 0.4 (15% ethyl acetate in chloroform); mp 288–291 °C; ^1^H NMR (CDCl_3_, 400 MHz): *δ* (ppm) 8.69–8.60 (m, 3H, ArH), 8.39 (t, *J* = 7.48 Hz, 1H, ArH), 8.15 (s, 1H, ArH), 7.97–7.94 (m, 1H, ArH), 7.86 (t, *J* = 8.12 Hz, 1H, ArH), 7.70 (d, *J* = 8.36 Hz, 1H, ArH), 7.66 (d, *J* = 8.52 Hz, 1H, ArH), 7.56–7.41 (m, 3H, ArH), 7.34 (t, *J* = 7.44 Hz, 1H, ArH), 6.14–6.00 (m, 2H, allyl-CH), 5.38 (d, *J* = 17.12 Hz, 2H, allyl-CH_2_), 5.25 (d, *J* = 10.36 Hz, 2H, allyl-CH_2_), 4.87 (d, *J* = 5.60 Hz, 4H, allyl-CH_2_); ^13^C NMR (CDCl_3_, 100 MHz): *δ* (ppm) 164.2, 164.0, 153.8, 147.9, 135.9, 133.7, 133.2, 132.9, 132.2, 131.3, 131.0, 128.4, 127.0, 126.7, 126.1, 125.9, 125.6, 124.5, 123.8, 123.7, 123.1, 122.6, 122.4, 121.2, 117.4, 115.6, 113.2, 110.2 (Ar*C*), 42.4 (allyl-*C*H_2_); MS (ESI): *m*/*z* 526.1 (M^+^ + 1); anal. calcd for C_33_H_23_N_3_O_2_S: C, 75.41; H, 4.41; N, 7.99; S, 6.10; found C, 75.35; H, 4.40; N, 7.90; S, 6.16.

#### 6-(1-Allyl-3-(benzo[*d*]thiazol-2-yl)-1*H*-indol-5-yl)-1*H*-benzo[*de*]isoquinoline-1,3(2*H*)-dione (16)

Yellow solid; 66% yield; *R*_f_ 0.5 (10% ethyl acetate in chloroform); mp 277–280 °C; ^1^H NMR (CDCl_3_, 400 MHz): *δ* (ppm) 8.70–8.62 (m, 3H, ArH), 8.58 (s, 1H, NH), 8.50–8.41 (m, 1H, ArH), 7.99 (d, *J* = 2.80 Hz, 1H, ArH), 7.96 (d, *J* = 8.04 Hz, 1H, ArH), 7.89 (t, *J* = 7.56 Hz, 1H, ArH), 7.85 (t, *J* = 4.76 Hz, 1H, ArH), 7.75–7.68 (m, 1H, ArH), 7.58–7.55 (m, 1H, ArH), 7.46–7.41 (m, 2H, ArH), 7.33 (t, *J* = 7.76 Hz, 1H, ArH), 6.17–6.07 (m, 1H, allyl-CH), 5.40 (d, *J* = 10.36 Hz, 1H, allyl-CH_2_), 5.32 (d, *J* = 17.20 Hz, 1H, allyl-CH_2_), 4.92 (d, *J* = 5.40 Hz, 2H, allyl-CH_2_); ^13^C NMR (CDCl_3_, 100 MHz): *δ* (ppm) 162.0, 161.1, 160.9, 153.9, 149.7, 136.9, 134.8, 133.3, 133.1, 132.1, 131.7, 131.0, 130.9, 130.8, 128.8, 128.6, 128.5, 127.2, 126.2, 126.1, 125.0, 124.5, 123.2, 122.3, 121.3, 119.0, 117.2, 111.9, 110.6 (Ar*C*), 49.6 (allyl-*C*H_2_); MS (ESI): *m*/*z* 486.1 (M^+^ + 1); anal. calcd for C_30_H_19_N_3_O_2_S: C, 74.21; H, 3.94; N, 8.65; S, 6.60; found C, 74.15; H, 3.99; N, 8.60; S, 6.66.

#### 6-(1-Allyl-3-(benzo[*d*]thiazol-2-yl)-1*H*-indol-5-yl)-2-benzyl-1*H*-benzo[*de*]isoquinoline-1,3(2*H*)-dione (17)

Yellow solid; 74% yield; *R*_f_ 0.5 (10% ethyl acetate in chloroform); mp 281–284 °C; ^1^H NMR (CDCl_3_, 400 MHz): *δ* (ppm) 8.70–8.61 (m, 3H, ArH), 8.38 (d, *J* = 8.12 Hz, 1H, ArH), 7.97–7.93 (m, 2H, ArH), 7.85–7.81 (m, 2H, ArH), 7.69–7.64 (m, 2H, ArH), 7.59 (d, *J* = 7.40 Hz, 2H, ArH), 7.55 (t, *J* = 8.52 Hz, 1H, ArH), 7.47–7.40 (m, 2H, ArH), 7.35 (t, *J* = 7.20 Hz, 3H, ArH), 6.16–6.06 (m, 1H, allyl-CH), 5.44 (s, 2H, benzyl-CH_2_), 5.39 (d, *J* = 10.32 Hz, 1H, allyl-CH_2_), 5.31 (d, *J* = 17.12 Hz, 1H, allyl-CH_2_), 4.90 (d, *J* = 5.40 Hz, 2H, allyl-CH_2_); ^13^C NMR (CDCl_3_, 100 MHz): *δ* (ppm) 163.5, 163.4, 162.2, 161.6, 153.9, 153.6, 149.0, 146.4, 136.9, 134.2, 132.2, 131.9, 130.8, 129.3, 128.6, 127.0, 126.2, 125.2, 124.5, 124.4, 124.4, 123.1, 122.5, 122.5, 122.4, 122.3, 121.3, 120.9, 118.9, 117.8, 115.0, 110.5 (Ar*C*), 49.6 (allyl-*C*H_2_), 43.4 (benzyl-*C*H_2_); MS (ESI): *m*/*z* 576.2 (M^+^ + 1); anal. calcd for C_37_H_25_N_3_O_2_S: C, 77.20; H, 4.38; N, 7.30; S, 5.57; found C, 77.25; H, 4.30; N, 7.38; S, 5.62.

#### 6-(1-Allyl-3-(benzo[*d*]thiazol-2-yl)-1*H*-indol-5-yl)-2-(phenylamino)-1*H*-benzo[*de*]isoquinoline-1,3(2*H*)-dione (18)

Yellow solid; 65% yield; *R*_f_ 0.4 (10% ethyl acetate in chloroform); mp 273–276 °C; ^1^H NMR (CDCl_3_, 400 MHz): *δ* (ppm) 8.76 (d, *J* = 7.48 Hz, 1H, ArH), 8.72 (d, *J* = 7.20 Hz, 1H, ArH), 8.64 (s, 1H, ArH), 8.47 (d, *J* = 8.48 Hz, 1H, ArH), 7.99 (s, 1H, ArH), 7.97 (d, *J* = 8.16 Hz, 1H, ArH), 7.89 (d, *J* = 7.52 Hz, 1H, ArH), 7.86 (d, *J* = 7.92 Hz, 1H, ArH), 7.75 (t, *J* = 7.92 Hz, 1H, ArH), 7.58 (d, *J* = 8.44 Hz, 1H, ArH), 7.48–7.41 (m, 2H, ArH), 7.33 (t, *J* = 7.64 Hz, 1H, ArH), 7.29–7.25 (m, 1H, ArH), 7.00–6.96 (m, 4H, ArH), 6.17–6.08 (m, 1H, allyl-CH), 5.40 (d, *J* = 10.20 Hz, 1H, allyl-CH_2_), 5.32 (d, *J* = 17.04 Hz, 1H, allyl-CH_2_), 4.93 (d, *J* = 5.44 Hz, 2H, allyl-CH_2_); ^13^C NMR (CDCl_3_, 100 MHz): *δ* (ppm) 163.5, 163.3, 162.1, 161.5, 153.8, 148.9, 146.3, 136.8, 134.1, 132.8, 132.6, 132.2, 132.1, 131.8, 130.8, 130.7, 129.2, 128.6, 126.9, 127.0, 126.1, 125.1, 124.3, 123.1, 122.3, 122.2, 121.2, 120.8, 118.8, 114.9, 110.4 (Ar*C*), 49.5 (allyl-*C*H_2_); MS (ESI): *m*/*z* 598.2 (M^+^ + 1); anal. calcd for C_36_H_24_N_4_O_2_S: C, 74.98; H, 4.20; N, 9.72; S, 5.56; found C, 74.90; H, 4.15; N, 9.78; S, 5.50.

#### 6-(1-Allyl-3-(benzo[*d*]thiazol-2-yl)-1*H*-indol-5-yl)-2-cyclohexyl-1*H*-benzo[*de*]isoquinoline-1,3(2*H*)-dione (19)

Yellow solid; 69% yield; *R*_f_ 0.5 (10% ethyl acetate in chloroform); mp 285–288 °C; ^1^H NMR (CDCl_3_, 400 MHz): *δ* (ppm) 8.64–8.58 (m, 3H, ArH), 8.35 (t, *J* = 7.68 Hz, 1H, ArH), 7.97 (t, *J* = 8.40 Hz, 1H, ArH), 7.86–7.79 (m, 2H, ArH), 7.68 (t, *J* = 8.28 Hz, 2H, ArH), 7.57–7.40 (m, 3H, ArH), 7.34 (t, *J* = 7.44 Hz, 1H, ArH), 6.16–6.05 (m, 1H, allyl-CH), 5.39 (d, *J* = 10.20 Hz, 1H, allyl-CH_2_), 5.32 (d, *J* = 17.12 Hz, 1H, allyl-CH_2_), 5.11–5.04 (m, 1H, cyclohex-CH), 4.91 (d, *J* = 5.36 Hz, 2H, allyl-CH_2_), 2.65–2.56 (m, 2H, cyclohex-CH_2_), 1.94–1.90 (m, 3H, cyclohex-CH_2_), 1.80–1.72 (m, 2H, cyclohex-CH_2_), 1.53–1.33 (m, 3H, cyclohex-CH_2_); ^13^C NMR (CDCl_3_, 100 MHz): *δ* (ppm) 164.9, 164.7, 154.1, 147.5, 130.9, 130.7, 128.3, 126.8, 126.7, 126.1, 126.0, 125.9, 125.6, 124.5, 123.9, 123.4, 123.1, 123.0, 122.9, 122.4, 122.0, 121.2, 118.8, 115.5, 113.2, 110.3, 110.2 (Ar*C*), 53.7 (cyclohex-*C*H), 49.5 (allyl-*C*H_2_), 29.1 (cyclohex-*C*H_2_), 26.5 (cyclohex-*C*H_2_), 25.5 (cyclohex-*C*H_2_); MS (ESI): *m*/*z* 568.2 (M^+^ + 1); anal. calcd for C_36_H_29_N_3_O_2_S: C, 76.17; H, 5.15; N, 7.40; S, 5.65; found C, 76.25; H, 5.11; N, 7.45; S, 5.60.

#### 6-(1-Allyl-3-(benzo[*d*]thiazol-2-yl)-1*H*-indol-5-yl)-2-(2-(piperazin-1-yl)ethyl)-1*H*-benzo[*de*] isoquinoline-1,3(2*H*)-dione (20)

Yellow solid; 76% yield; *R*_f_ 0.3 (30% ethyl acetate in chloroform); mp 294–297 °C; ^1^H NMR (CDCl_3_, 400 MHz): *δ* (ppm) 8.66–8.59 (m, 3H, ArH), 8.37 (t, *J* = 8.20 Hz, 1H, ArH), 7.96–7.92 (m, 1H, ArH), 7.84–7.79 (m, 2H, ArH), 7.68–7.62 (m, 2H, ArH), 7.54–7.38 (m, 3H, ArH), 7.32 (t, *J* = 7.56 Hz, 1H, ArH), 6.14–6.03 (m, 1H, allyl-CH), 5.37 (d, *J* = 10.20 Hz, 1H, allyl-CH_2_), 5.29 (d, *J* = 17.08 Hz, 1H, allyl-CH_2_), 4.89 (d, *J* = 5.36 Hz, 2H, allyl-CH_2_), 4.40 (t, *J* = 6.92 Hz, 2H, ethyl-CH_2_), 2.90 (t, *J* = 4.72 Hz, 4H, pip-CH_2_), 2.74 (t, *J* = 7.24 Hz, 2H, ethyl-CH_2_), 2.60 (bs, 4H, pip-CH_2_); ^13^C NMR (CDCl_3_, 100 MHz): *δ* (ppm) 164.4, 164.2, 153.8, 147.8, 136.6, 135.9, 133.7, 133.1, 132.1, 131.1, 130.8, 128.7, 128.3, 127.0, 126.7, 126.1, 125.9, 124.5, 123.8, 123.1, 122.7, 121.2, 118.8, 115.6, 113.2, 111.7, 110.2 (Ar*C*), 56.3 (ethyl-*C*H_2_), 54.5 (pip-*C*H_2_), 49.5 (allyl-*C*H_2_), 46.0 (pip-*C*H_2_), 37.3 (ethyl-*C*H_2_); MS (ESI): *m*/*z* 568.2 (M^+^ + 1); anal. calcd for C_36_H_31_N_5_O_2_S: C, 72.34; H, 5.23; N, 11.72; S, 5.36; found C, 72.39; H, 5.28; N, 11.70; S, 5.32.

#### 6-(3-(Benzo[*d*]thiazol-2-yl)-1-propyl-1*H*-indol-5-yl)-2-(4-fluorophenyl)-1*H*-benzo[*de*]isoquinoline-1,3(2*H*)-dione (21)

Light brown solid; 61% yield; *R*_f_ 0.4 (20% ethyl acetate in chloroform); mp 288–291 °C; ^1^H NMR (CDCl_3_, 400 MHz): *δ* (ppm) 8.62 (t, *J* = 3.72 Hz, 2H, ArH), 8.58 (d, *J* = 7.28 Hz, 1H, ArH), 8.42 (d, *J* = 8.44 Hz, 1H, ArH), 8.36 (s, 1H, ArH), 7.97 (d, *J* = 7.64 Hz, 1H, ArH), 7.93 (t, *J* = 7.32 Hz, 1H, ArH), 7.86 (t, *J* = 8.16 Hz, 2H, ArH), 7.77 (d, *J* = 8.40 Hz, 1H, ArH), 7.49–7.39 (m, 4H, ArH), 7.36–7.31 (m, 3H, ArH), 6.21–6.11 (m, 1H, allyl-CH), 5.33 (d, *J* = 11.20 Hz, 1H, allyl-CH_2_), 5.29 (d, *J* = 18.40 Hz, 1H, allyl-CH_2_), 5.07 (d, *J* = 4.60 Hz, 2H, allyl-CH_2_); ^13^C NMR (CDCl_3_, 100 MHz): *δ* (ppm) 163.5, 163.3, 162.1, 161.6, 153.9, 153.4, 149.0, 146.4, 136.9, 134.2, 132.2, 131.9, 130.8, 129.3, 128.6, 127.3, 127.1, 127.0, 126.2, 125.8, 125.2, 124.4, 123.7, 123.1, 122.5, 121.3, 120.9, 118.9, 117.8, 115.0, 110.5 (Ar*C*), 49.6 (allyl-*C*H_2_); MS (ESI): *m*/*z* 580.1 (M^+^ + 1); anal. calcd for C_36_H_22_FN_3_O_2_S: C, 74.60; H, 3.83; N, 7.25; S, 5.53; found C, 74.69; H, 3.78; N, 7.38; S, 5.73.

#### 2-Amino-6-(3-(benzo[*d*]thiazol-2-yl)-1-propyl-1*H*-indol-5-yl)-1*H*-benzo[*de*]isoquinoline-1,3(2*H*)-dione (22)

Light brown solid; 62% yield; *R*_f_ 0.3 (20% ethyl acetate in chloroform); mp 289–292 °C; ^1^H NMR (CDCl_3_, 400 MHz): 8.73 (*J* = 7.16 Hz, 1H, ArH), 8.60 (s, 1H, ArH), 8.44 (d, *J* = 8.60 Hz, 1H, ArH), 7.99 (s, 1H, ArH), 7.96 (d, *J* = 8.12 Hz, 1H, ArH), 7.87 (t, *J* = 7.76 Hz, 2H, ArH), 7.73 (t, *J* = 8.08 Hz, 1H, ArH), 7.59 (d, *J* = 8.36 Hz, 1H, ArH), 7.46 (d, *J* = 7.40 Hz, 1H, ArH), 7.41 (m, 2H, ArH), 7.32 (t, *J* = 7.40 Hz, 1H, ArH), 5.60 (s, 2H, NH_2_), 4.28 (t, *J* = 7.16 Hz, 2H, propyl-CH_2_), 2.09–1.99 (m, 2H, propyl-CH_2_), 1.08 (t, *J* = 7.28 Hz, 3H, propyl-CH_3_); ^13^C NMR (CDCl_3_, 100 MHz): *δ* (ppm) 161.0, 153.8, 148.8, 136.9, 136.8, 133.9, 133.6, 132.6, 132.1, 131.4, 131.2, 130.7, 130.6, 128.5, 127.4, 126.8, 126.0, 125.9, 125.0, 124.2, 123.0, 122.1, 121.2, 111.6, 110.3 (Ar*C*), 48.7 (propyl-*C*H_2_), 23.4 (propyl-*C*H_2_), 11.5 (propyl-*C*H_3_); MS (ESI): *m*/*z* 503.1 (M^+^ + 1); anal. calcd for C_30_H_22_N_4_O_2_S: C, 71.69; H, 4.41; N, 11.15; S, 6.38; found C, 71.62; H, 4.39; N, 11.10; S, 6.31.

### 6.2 MTT assay protocol

Human malignant cell lines, *i.e.*, MCF7 (breast), HeLa (cervix), and A549 (lung), as well as non-cancerous human cell line Hek293 (kidney), were cultured using the Ham's media or DMEM having 10% FBS, 100 mg ml^−1^ streptomycin, 100 U ml^−1^ penicillin, and 50 mM glutamine. For seeding of cells, 96 well plates were used, maintaining the density of 1 × 10^−5^ cells per well with the help of DMEM as a media having 10% FBS cells. Then, seeded cells were incubated in an incubator having a 5% CO_2_ supply at 37 °C. Compounds were added to the wells at 1, 10, and 100 μM concentrations, and cells were incubated for 48 h at 37 °C and then added 10 μl of MTT to each well prepared from 5 mg ml^−1^ stock solution using 1 × PBS buffer and were incubated for 4 h at 37 °C in the dark. DMSO (100 μl) was used to dissolve the formazan crystals. To determine the concentration of formazan, the crystal absorbance difference was recorded at 570 nm. The percentage of relative cell toxicity was calculated by using the following eqn (1):1
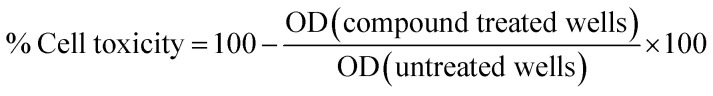


### 6.3. Relaxation assay of human topoisomerase II*a*

The assay of human type II*a* topoisomerase mediated relaxation of supercoiled plasmid DNA was performed using reaction buffer (20 μl) (5 mM dithiothreitol, 0.5 M Tris–HCl, pH 8.0, 20 mM ATP, 1.50 M NaCl, 300 μg ml^−1^ BSA, and 100 mM MgCl_2_) having supercoiled pHOT1 plasmid DNA (500 ng) and human type II*a* topoisomerase enzyme (4 units). The reaction mixtures were incubated for 30 min at 37 °C, followed by adding 10% sodium dodecyl sulfate (2 μl) to stop the reaction. Added 0.5 mg ml^−1^ proteinase K (1 μl) to the reaction mixture and allowed to incubate for 15 min at 37 °C. Then, added loading buffer, *i.e.*, 0.25% bromophenol blue and 50% glycerol (1 μl) to the reaction mixture and run on 1% agarose gel electrophoresis using TAE buffer (1.14 ml acetic acid, and 0.37 g EDTA pH 8.1, and 100 ml of 10× stock solution–4.8 g of Tris base). The gel containing DNA was stained with ethidium bromide, washed, and photographed using UV light.

### 6.4 Preparation of stock solution

The stock solutions of HSA and compounds (12 and 13) (10^−3^ M) were made by dissolving into distilled water and DMSO, respectively.

### 6.5 UV-visible spectroscopic study

To record the absorption spectra of HSA, a fixed amount of HSA (10 μM) was titrated with the increasing concentrations of compounds 12 and 13 (7 μM) in the phosphate buffer of pH 7.4 at 298 K. The phosphate buffer was used as a blank solution for baseline corrections. The absorption spectra were recorded in the range of 200–800 nm. Benesi–Hildebrand eqn (2) was used to calculate the binding constants (*K*_b_):2
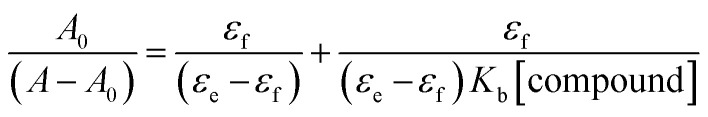
In this equation, *A*_0_ and *A* denote the absorbance of HSA in the free form and presence of compounds (12 and 13), respectively, while *ε*_f_ and *ε*_b_ denote the molar extinction coefficients of HSA in the free form and presence of compounds (12 and 13), respectively. To calculate the *K*_b_ value, the ratio of intercept to the slope was used as *A*_0_/(*A* − *A*_0_) *versus* 1/[compound] plots.

### 6.6 Fluorescence study

To record the emission spectra, a fixed amount of HSA (10 μM) was titrated with increasing concentration of compounds 12 and 13 (33 μM) in phosphate buffer of pH 7.4 at 298 K, 308 K, and 318 K. All spectra were recorded in the range of 200–800 nm at excitation wavelength 280 nm and a constant slit width was used for excitation and emission throughout the experiments.

To determine the quenching constant, following Stern–Volmer eqn (3) was used.3
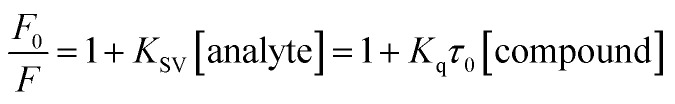
where, *F*_0_ and *F* denote the emission intensity of HSA in free form and the presence of compounds 12 and 13, respectively. The plots of *F*_0_/*F versus* [compound] were used to determine the Stern–Volmer quenching constants (*K*_SV_) and bimolecular quenching constants (*K*_q_).

Further to determine the binding constants (*K*_b_) and the average number of binding sites (*n*), the following modified Stern–Volmer eqn (4) was used.4
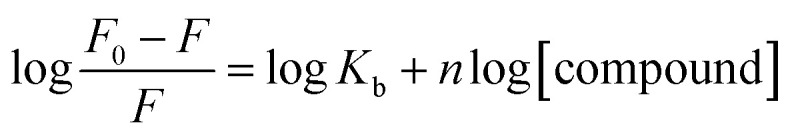
where *F*_0_ and *F* are the same as in eqn (3). The values of *K*_b_ and *n* were determined from intercept and slope, respectively, as log{(*F*_0_ − *F*)/*F versus* log[compound]} plots.

The thermodynamic parameters such as the change in enthalpy (Δ*H*) and change in entropy (Δ*S*) were determined by following the van't Hoff eqn (5):5
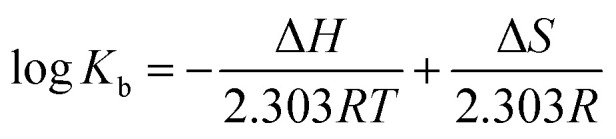
*R* and *T* denote the gas constant and absolute temperature, respectively.

The change in free energy (Δ*G*) for various temperatures was determined by the following eqn (6):

6 Δ*G* = Δ*H* − *T*Δ*S*

### 6.7 Synchronous fluorescence study

The synchronous fluorescence experiment of HSA was performed at a fixed amount of HSA and increasing concentrations of compounds 12 and 13. The spectra were recorded by taking the difference between the wavelengths of excitation and emission (Δ*λ* = *λ*_emi_ − *λ*_exc_) at 15 nm and 60 nm.

### 6.8 Excited-state fluorescence lifetime analysis

Time-resolved fluorescence was recorded by taking a fixed amount of HSA (10 μM) with incremental additions of compounds (12 and 13) (0–20 μM) by maintaining the ratio of HSA : compound = 1 : 0, 1 : 1, 1 : 5 and 1 : 10. The spectra were recorded at 345 nm emission wavelength in phosphate buffer of pH 7.4 at 298 K. To determine the average fluorescence lifetime (*τ*), the following eqn (7) was used.

7 τ = ∑*τ*_i_*α*_i_

where *τ*_i_ and *α*_i_ denote decay time and relative amplitude, respectively.

### 6.9 Energy transfer between HSA and compounds

The energy transfer study was accomplished by recording the HSA emission spectrum at 280 nm excitation wavelength and the absorption spectra of compounds 12 and 13.

The following eqn (8)–(10) were used to determine the donor to acceptor energy transfer (*E*), the critical distance required for energy transfer by 50% (*R*_0_), overlap integral of the emission spectrum of donor with the absorption spectrum of the acceptor (*J*), and distance among the donor and the acceptor (*r*):^39,57^8
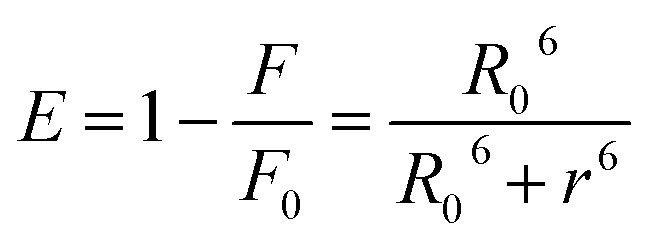
In this equation, *F*_0_ and *F* denote the emission intensities of HSA in free form and the presence of compounds 12 and 13, respectively.

9 *R*_0_^6^ = 8.8 × 10^−25^*k*^2^*η*^−4^*ϕJ*

where *k*^2^, *η*, *ϕ* denote the orientation factor of dipoles, refracted index for used medium, and fluorescence quantum yield of the donor.10
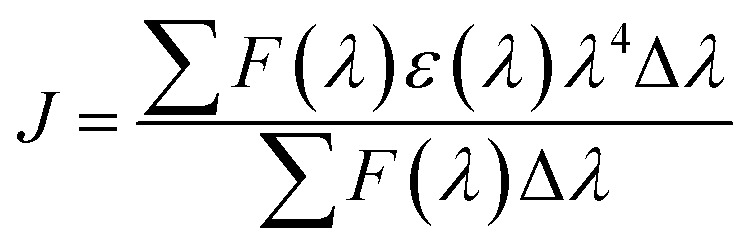
In this equation, *F*(*λ*) denotes the emission intensity for the free form of the donor at the wavelength *λ*, whereas *ε*(*λ*) denotes the molar absorption coefficient of acceptor at the wavelength *λ*.

### 6.10 Docking study

Molecular docking studies of compounds 12 and 13 with 3-D X-ray structure of human type II*a* topoisomerase (PDB: 1ZXM) were performed by the tools of the AutoDock software package (vina). The Gaussian 09W package was used to optimize the ligands' 3D structures. The molecules were prepared using gauss view 5 software, and further structure optimization was carried out using density functional theory utilizing B3LYP function and 6-31g(d) basis set. These structures were saved in PDB format. AutoDockTools (1.5.6rc3) setup of the graphical user interface was used for the setting of each interaction between compounds and topoisomerase. All the hydrogen atoms to the target topoisomerase were added and Gasteiger charges were determined. The partial charges of PDB files of ligands were altered with the purpose that the charges of the nonpolar hydrogens could be allocated to hydrogen attached atoms and the files were saved in Pdbqt format. A grid having a spacing of 0.375 Å along with directing in *x*, *y*, and *z* directions 126 Å, 100 Å, 126 Å, respectively, was built.

## Conflicts of interest

The author declared no conflict of interest

## Supplementary Material

RA-012-D1RA04148G-s001
