# Supplementary material for: Synthesis and photobiological applications of naphthalimide–benzothiazole conjugates: cytotoxicity and topoisomerase IIα inhibition
Source: RSC Adv. 2021 Dec 22;12(1):483–97. doi: 10.1039/d1ra04148g (PMC8694140; doi:10.1039/d1ra04148g)
Supplement: RA-012-D1RA04148G-s001 [file RA-012-D1RA04148G-s001.pdf]

Electronic Supplementary Material (ESI) for RSC Advances.
This journal is © The Royal Society of Chemistry 2021

Synthesis and photobiological applications of naphthalimide-benzothiazole conjugates: Cytotoxicity and topoisomerase IIα inhibition

*Iqubal Singh, Vijay Luxami, Diptiman Choudhury and Kamaldeep Paul**

School of Chemistry and Biochemistry, Thapar Institute of Engineering and Technology, Patiala-147001, India

Email: kpaul@thapar.edu

Table of Contents

	Contents	Page No
**Figures S1-S55**	^{1}H, ^{13}C NMR, and mass spectra of compounds	S2-S29
**Figures S56**	Agarose gel stained with ethidium bromide for inhibitoty activity towards Topo IIα relaxation by compounds **12** and **13**	S29
**Figure S57**	Benesi-Hildebrand plot of HSA for absorption spectra on incremental addition of compounds **12** and **13**	S30
**Figure S58**	Effect of incremental addition of compound **12** on emission spectra of HSA at 308 K and 318 K	S30
**Figure S59**	Effect of incremental addition of compound **13** on emission spectra of HSA at 308 K and 318 K	S31
**Figure S60**	Stern-Volmer plots of compound **12** with HSA	S31
**Figure S61**	Stern-Volmer plots of compound **12** with HSA	S32
**Figure S62**	Modified Stern-Volmer plots of compound **12** with HSA	S33
**Figure S63**	Modified Stern-Volmer plots of compound **13** with HSA	S34
**Figure S64**	Van't Hoff plots for interaction of compounds **12** and **13** with HSA	S35
**Figure S65**	Fluorescence lifetime spectra of HSA in free form and the presence of compounds **12** (a) and **13** (b)	S35
**Figure S66**	Synchronous fluorescence spectra of the HSA with increasing concentration of compound **13**	S36
**Table S1**	Binding energy of compounds **8-11** based upon docking studies	S36
**Table S2**	Binding energy of compounds **12-15** based upon docking studies	S37
**Table S3**	Binding energy of compounds **16-19** based upon docking studies	S37
**Table S4**	Binding energy of compounds **20-22** and etoposide based upon docking studies	S38

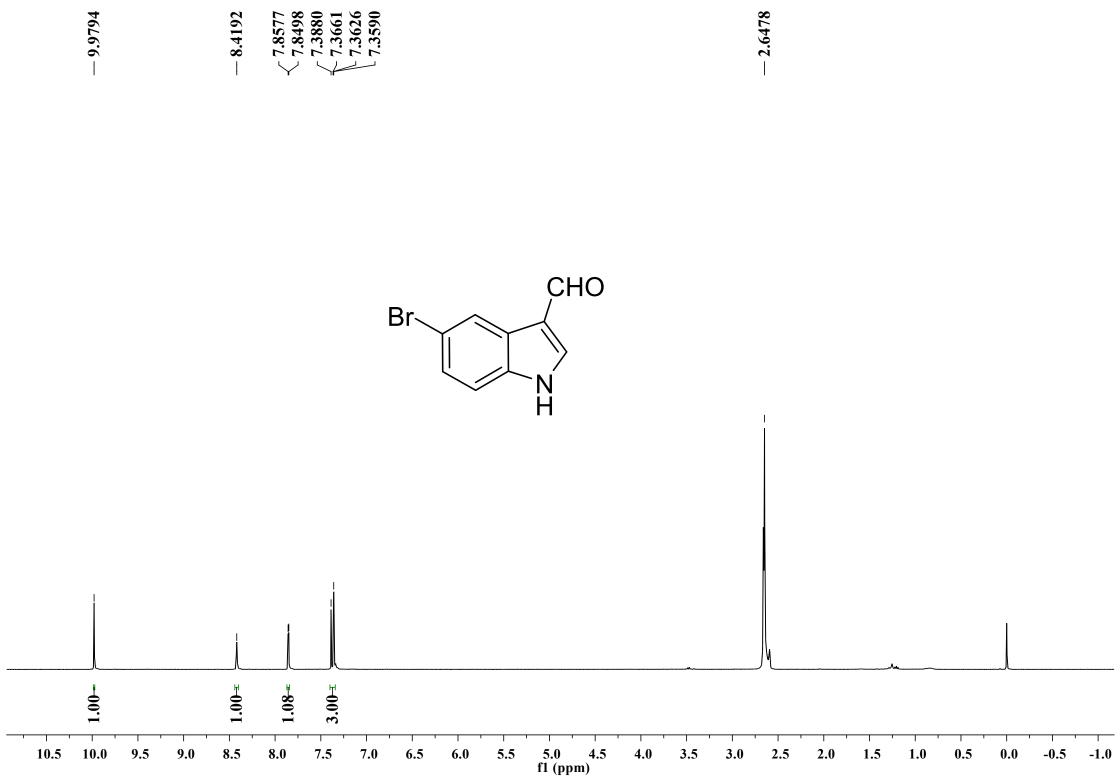

Figure S1: ^{1}H NMR spectrum of 5-bromo-1*H*-indole-3-carbaldehyde (**2**)

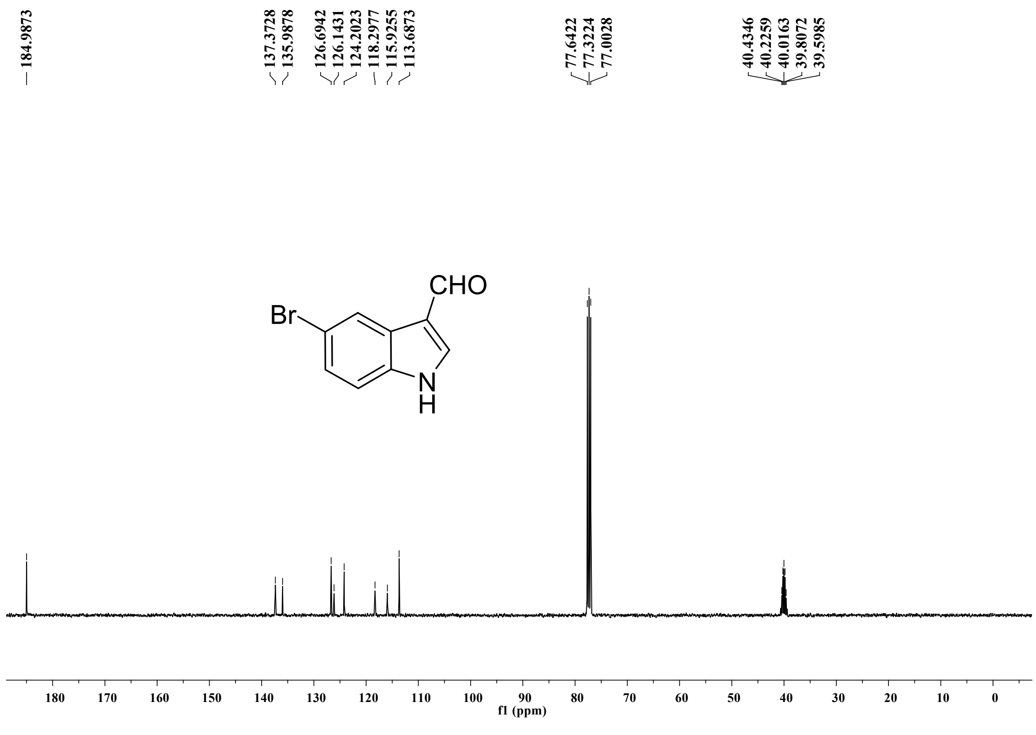

Figure S2: ^{13}C NMR spectrum of 5-bromo-1*H*-indole-3-carbaldehyde (**2**)

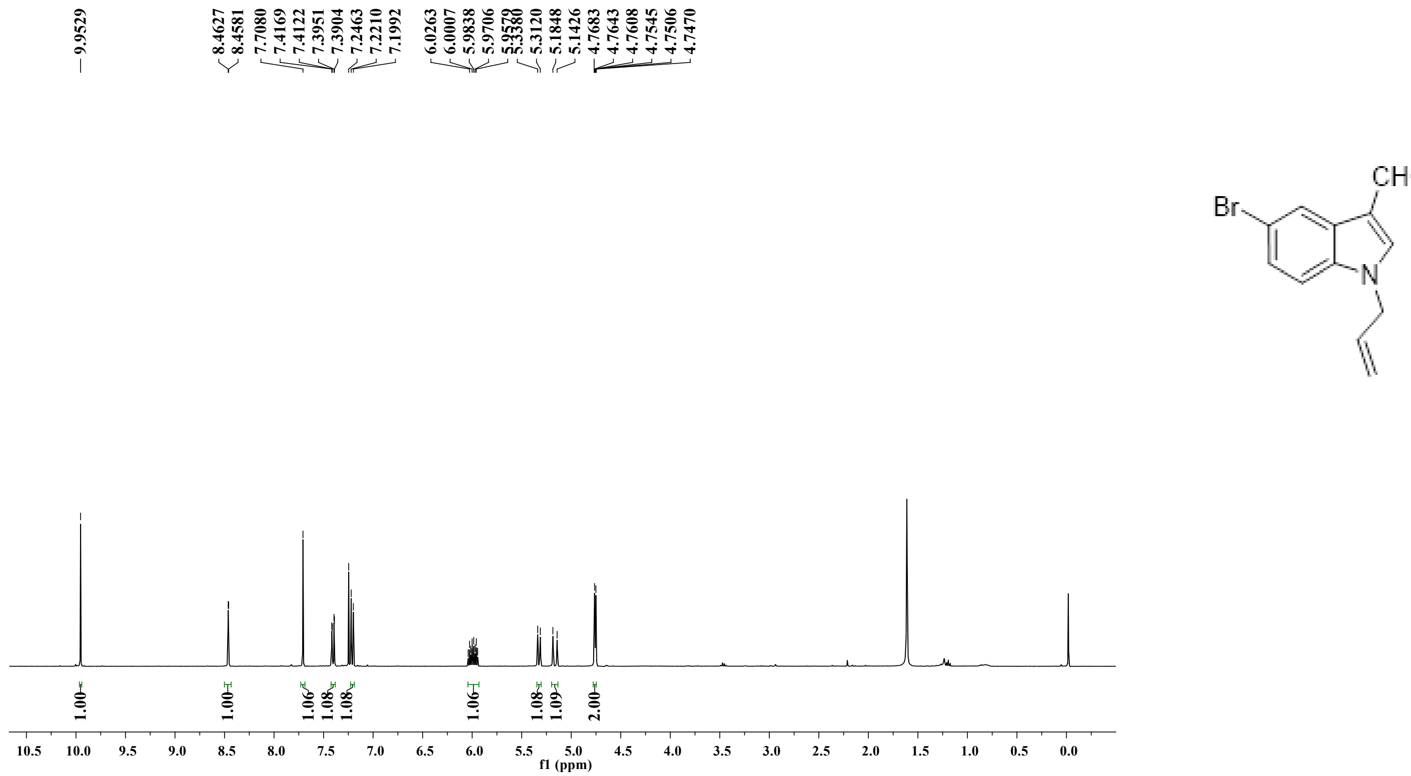

Figure S3: ^{1}H NMR spectrum of 1-allyl-5-bromo-1*H*-indole-3-carbaldehyde (**3**)

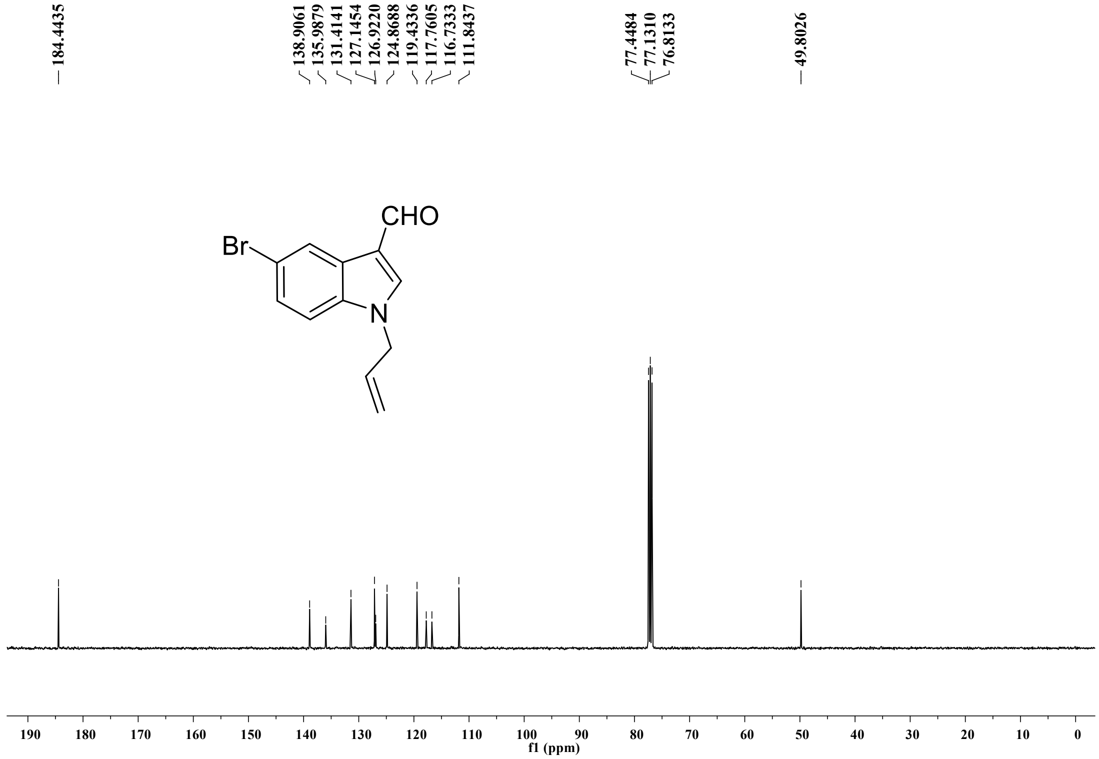

Figure S4: ^{13}C NMR spectrum of 1-allyl-5-bromo-1*H*-indole-3-carbaldehyde (**3**)

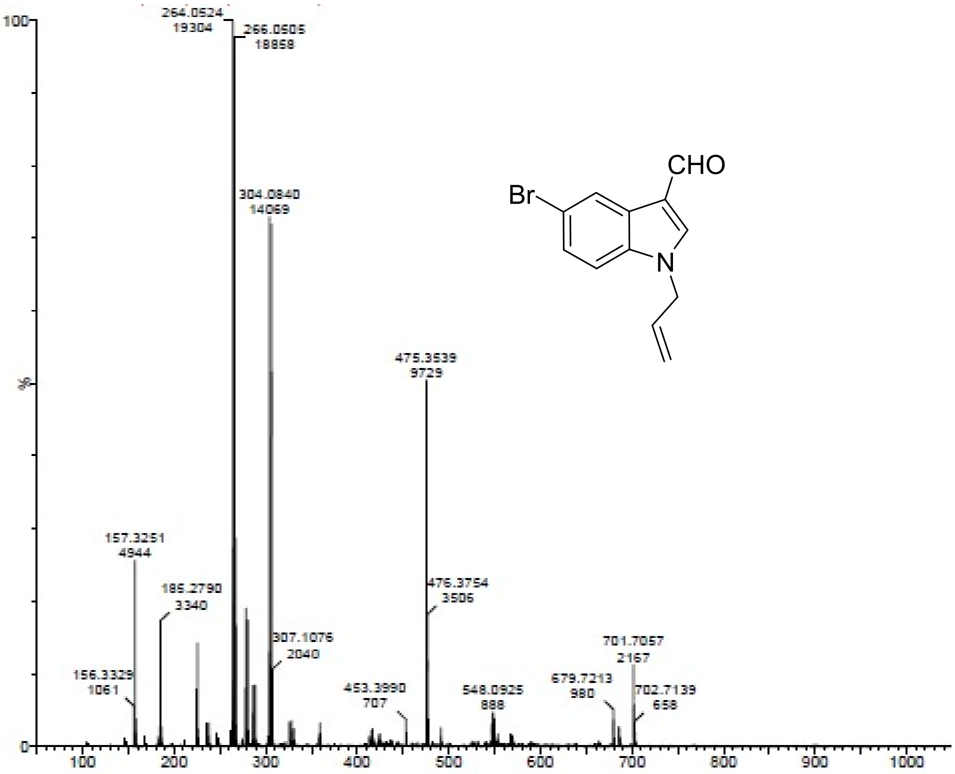

Figure S5: Mass spectrum of 1-allyl-5-bromo-1*H*-indole-3-carbaldehyde (**3**)

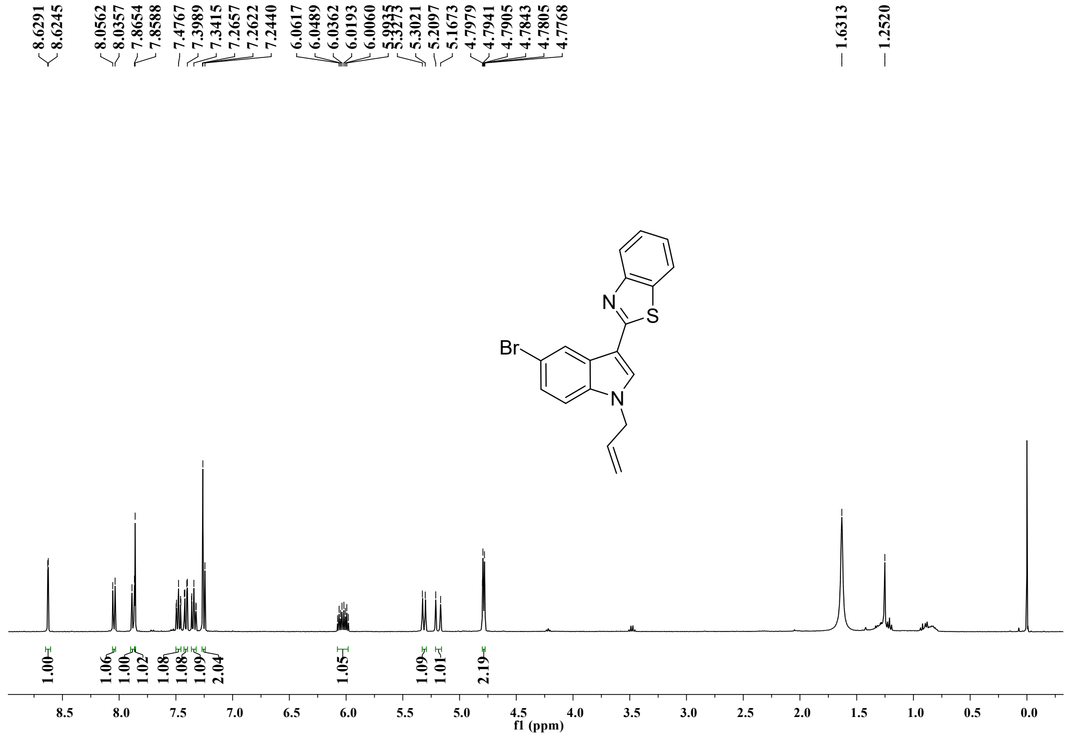

Figure S6: ^{1}H NMR spectrum of 2-(1-allyl-5-bromo-1*H*-indol-3-yl)benzo[*d*]thiazole (**4**)

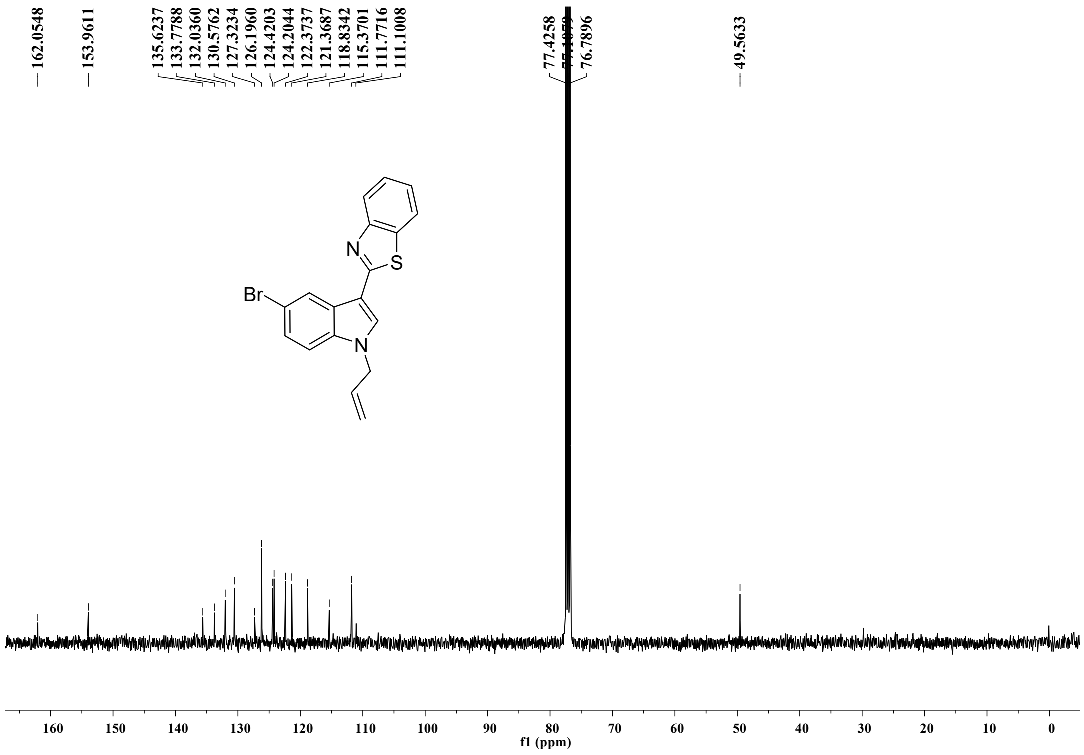

Figure S7: ^{13}C NMR spectrum of 2-(1-allyl-5-bromo-1*H*-indol-3-yl)benzo[*d*]thiazole (**4**)

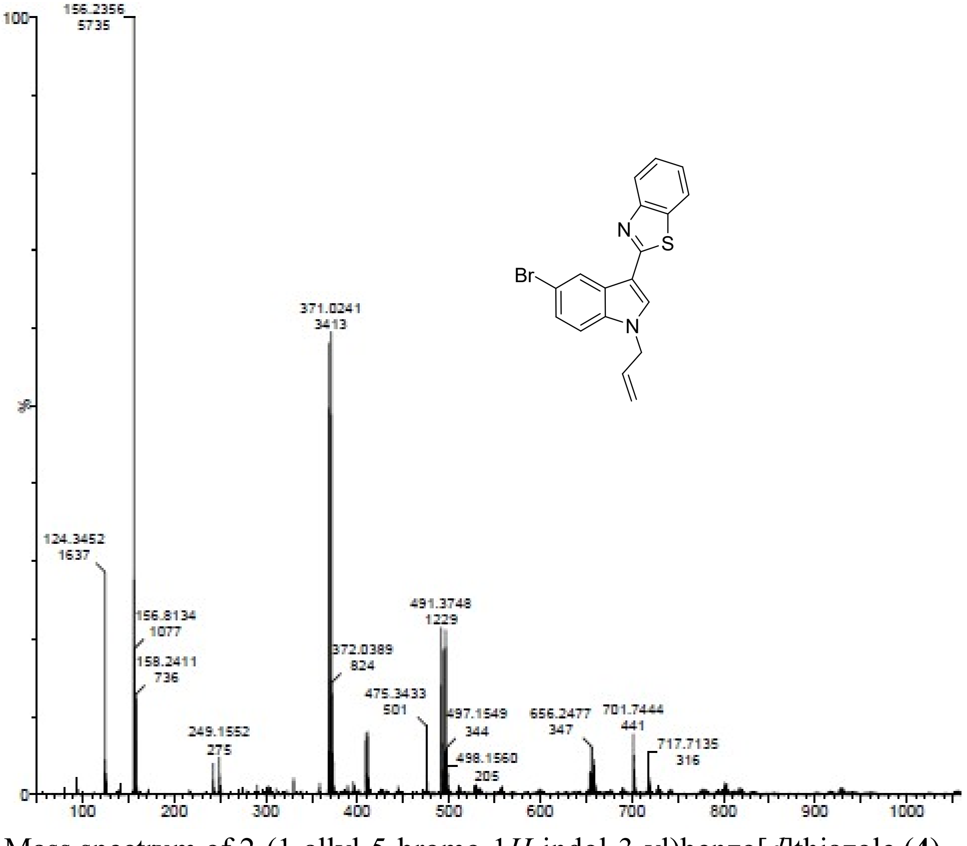

Figure S8: Mass spectrum of 2-(1-allyl-5-bromo-1*H*-indol-3-yl)benzo[*d*]thiazole (**4**)

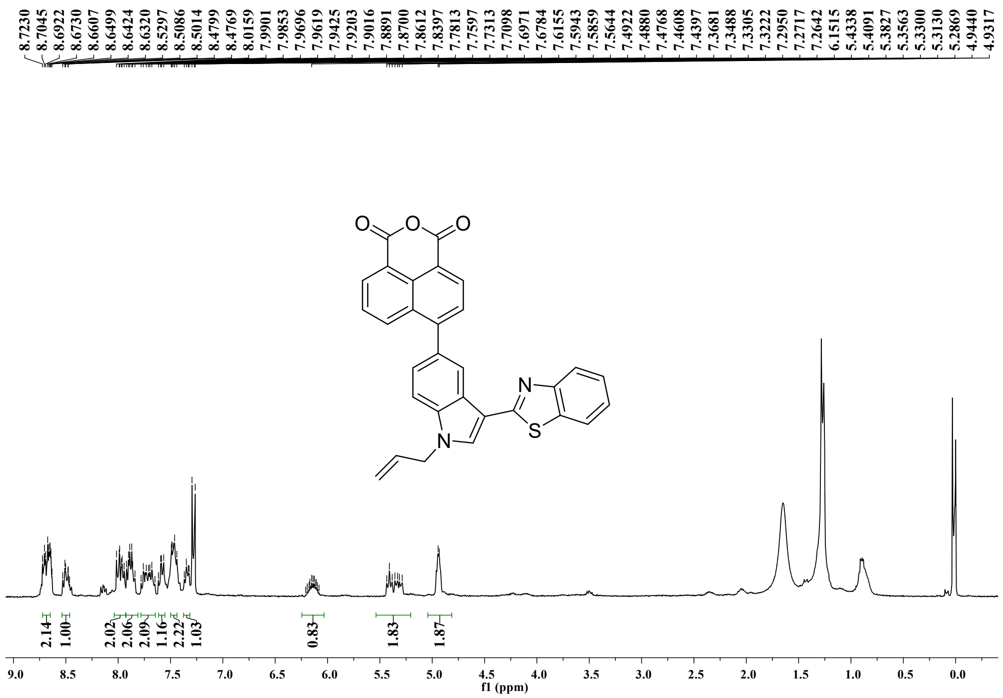

Figure S9: ^{1}H NMR spectrum of 6-(1-allyl-3-(benzo[*d*]thiazol-2-yl)-1*H*-indol-5-yl)-1*H*,3*H*-benzo[*de*]isochromene-1,3-dione (**7**)

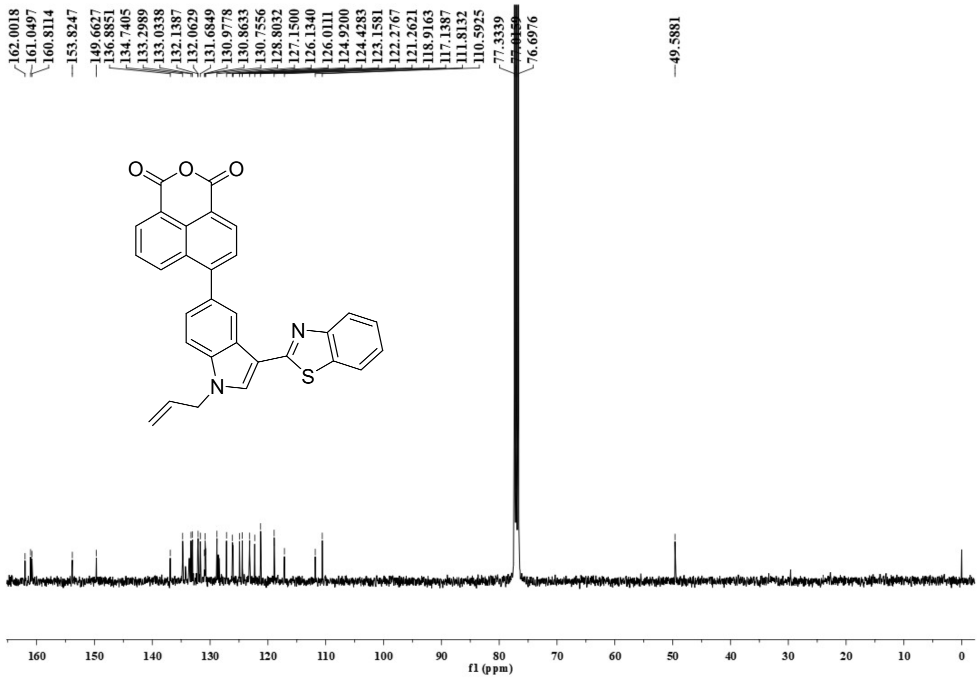

Figure S10: ^{13}C NMR spectrum of 6-(1-allyl-3-(benzo[*d*]thiazol-2-yl)-1*H*-indol-5-yl)-1*H*,3*H*-benzo[*de*]isochromene-1,3-dione (7)

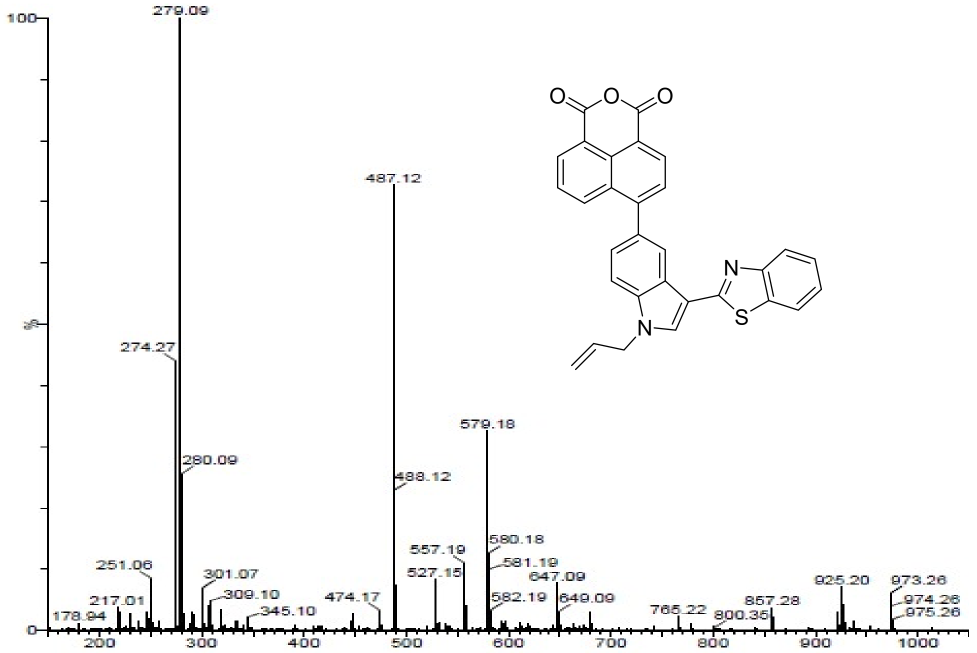

Figure S11: Mass spectrum of 6-(1-allyl-3-(benzo[*d*]thiazol-2-yl)-1*H*-indol-5-yl)-1*H*,3*H*-benzo[*de*]isochromene-1,3-dione (**7**)

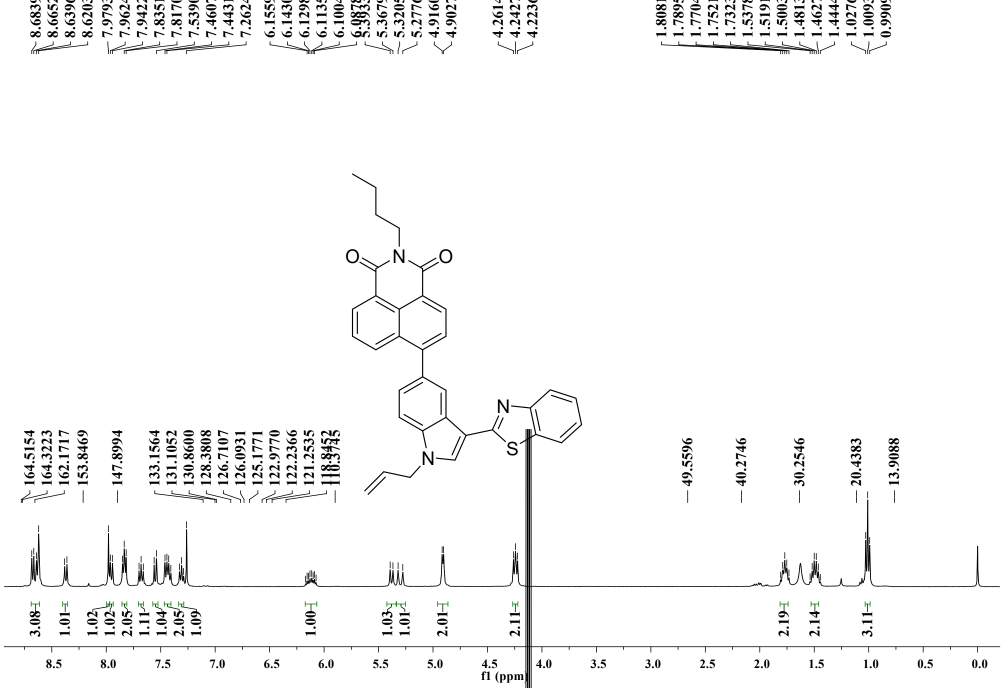

Figure S12: ^{1}H NMR spectrum of 6-(1-allyl-3-(benzo[*d*]thiazol-2-yl)-1*H*-indol-5-yl)-2-butyl-1*H*-benzo[*de*]isoquinoline-1,3(2*H*)-dione (**8**)

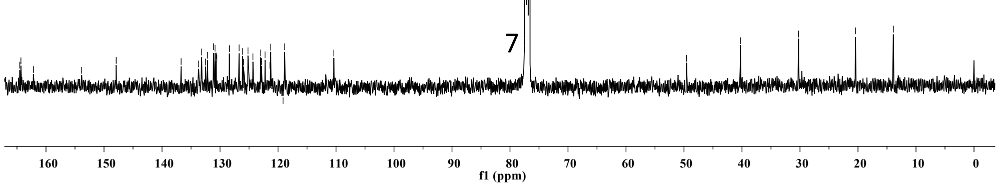

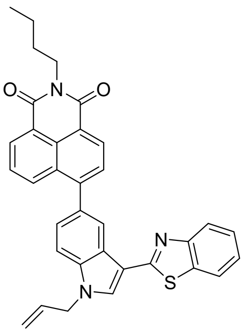

Fgure S13: ^{13}C NMR spectrum of 6-(1-allyl-3-(benzo[*d*]thiazol-2-yl)-1*H*-indol-5-yl)-2-butyl-1*H*-benzo[*de*]isoquinoline-1,3(2*H*)-dione (**8**)

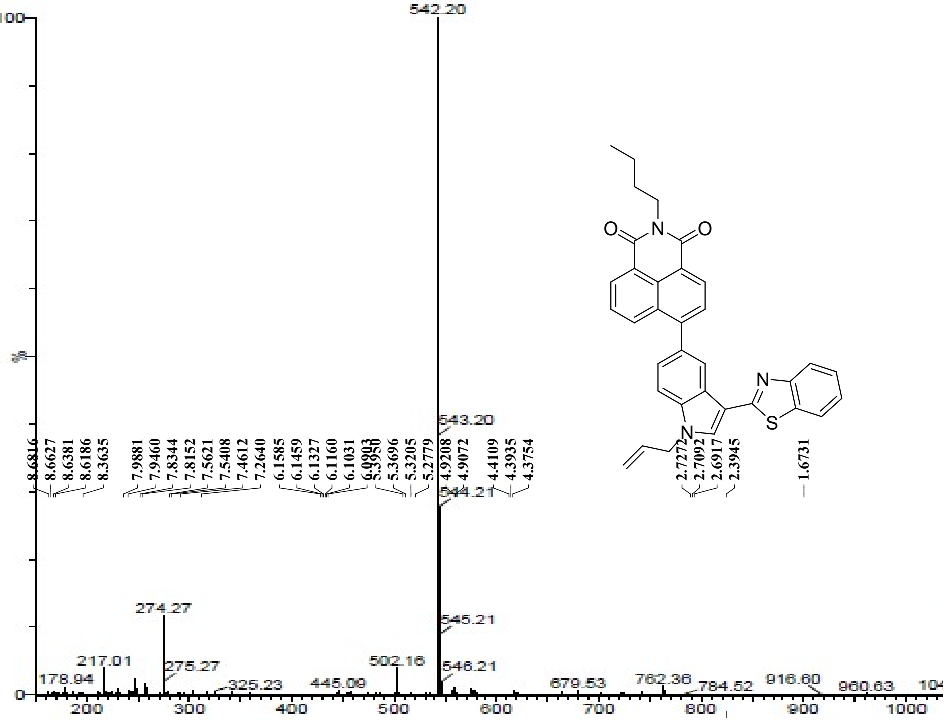

Figure S14: Mass spectrum of 6-(1-allyl-3-(benzo[*d*]thiazol-2-yl)-1*H*-indol-5-yl)-2-butyl-1*H*-benzo[*de*]isoquinoline-1,3(2*H*)-dione (**8**)

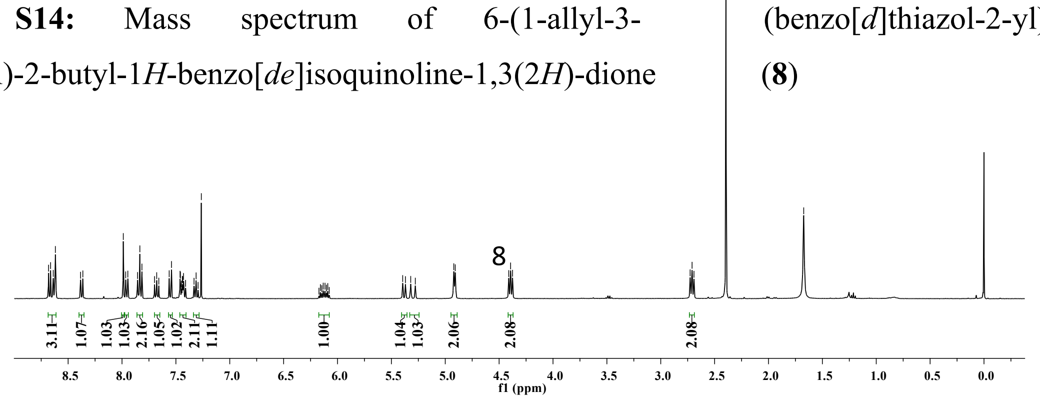

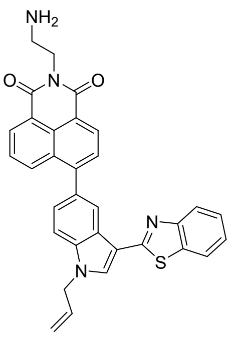

Figure S15: ^{1}H NMR spectrum of 6-(1-allyl-3-(benzo[*d*]thiazol-2-yl)-1*H*-indol-5-yl)-2-(2-aminoethyl)-1*H*-benzo[*de*]isoquinoline-1,3(2*H*)-dione (**9**)

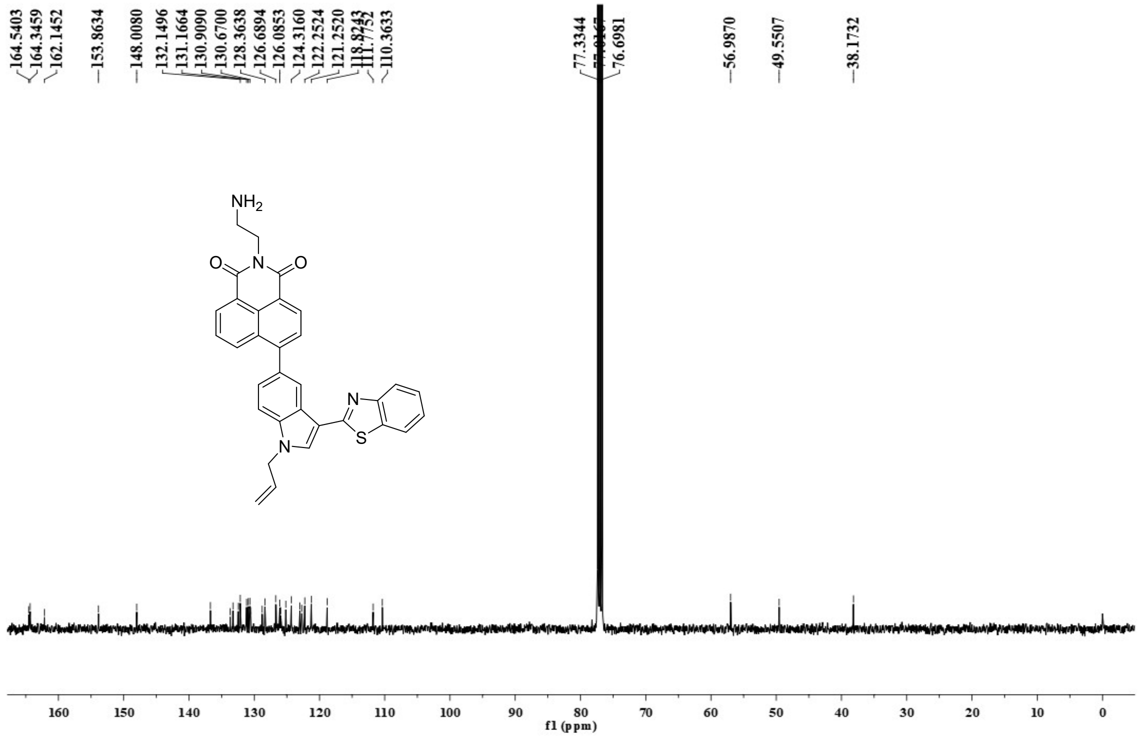

Figure S16: ^{13}C NMR spectrum of 6-(1-allyl-3-(benzo[*d*]thiazol-2-yl)-1*H*-indol-5-yl)-2-(2-aminoethyl)-1*H*-benzo[*de*]isoquinoline-1,3(2*H*)-dione (**9**)

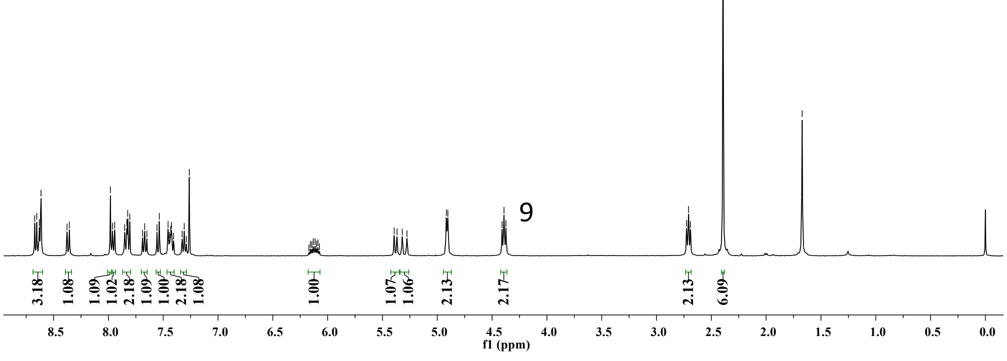

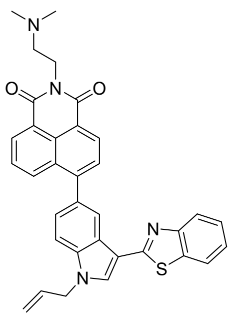

Figure S17: ^{1}H NMR spectrum of 6-(1-allyl-3-(benzo[*d*]thiazol-2-yl)-1*H*-indol-5-yl)-2-(2-(dimethylamino)ethyl)-1*H*-benzo[*de*]isoquinoline-1,3(2*H*)-dione (**10**)

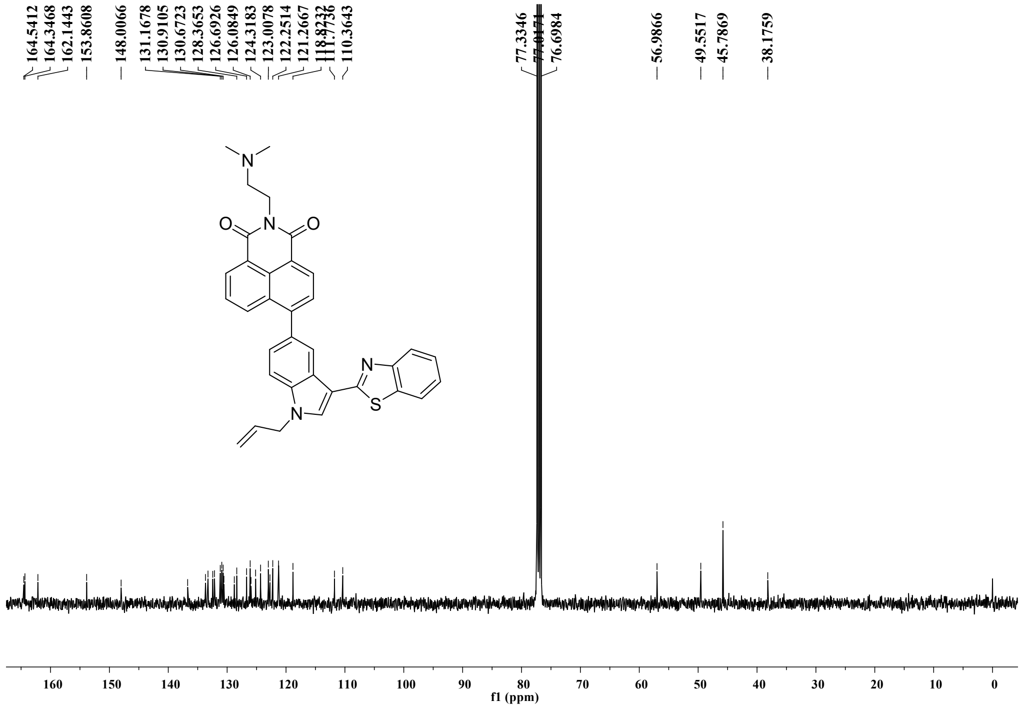

Figure S18: ^{13}C NMR spectrum of 6-(1-allyl-3-(benzo[*d*]thiazol-2-yl)-1*H*-indol-5-yl)-2-(2-(dimethylamino)ethyl)-1*H*-benzo[*de*]isoquinoline-1,3(2*H*)-dione (**10**)

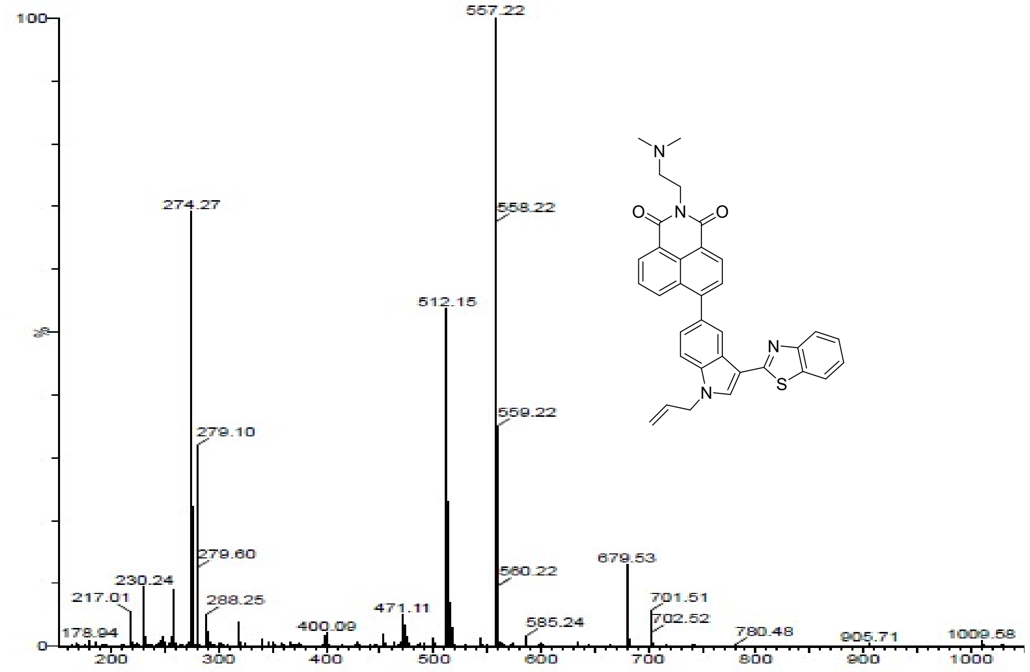

Figure S19: Mass spectrum of 6-(1-allyl-3-(benzo[*d*]thiazol-2-yl)-1*H*-indol-5-yl)-2-(2-(dimethylamino)ethyl)-1*H*-benzo[*de*]isoquinoline-1,3(2*H*)-dione (**10**)

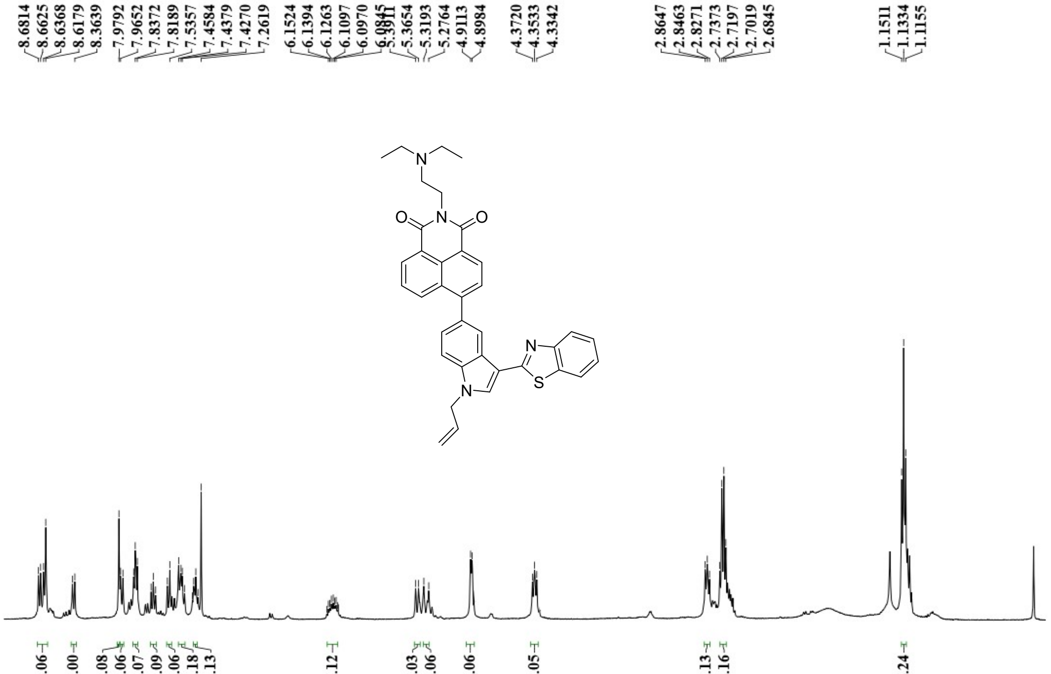

Figure S20: ^{1}H NMR spectrum of 6-(1-allyl-3-(benzo[*d*]thiazol-2-yl)-1*H*-indol-5-yl)-2-(2-(diethylamino)ethyl)-1*H*-benzo[*de*]isoquinoline-1,3(2*H*)-dione (**11**)

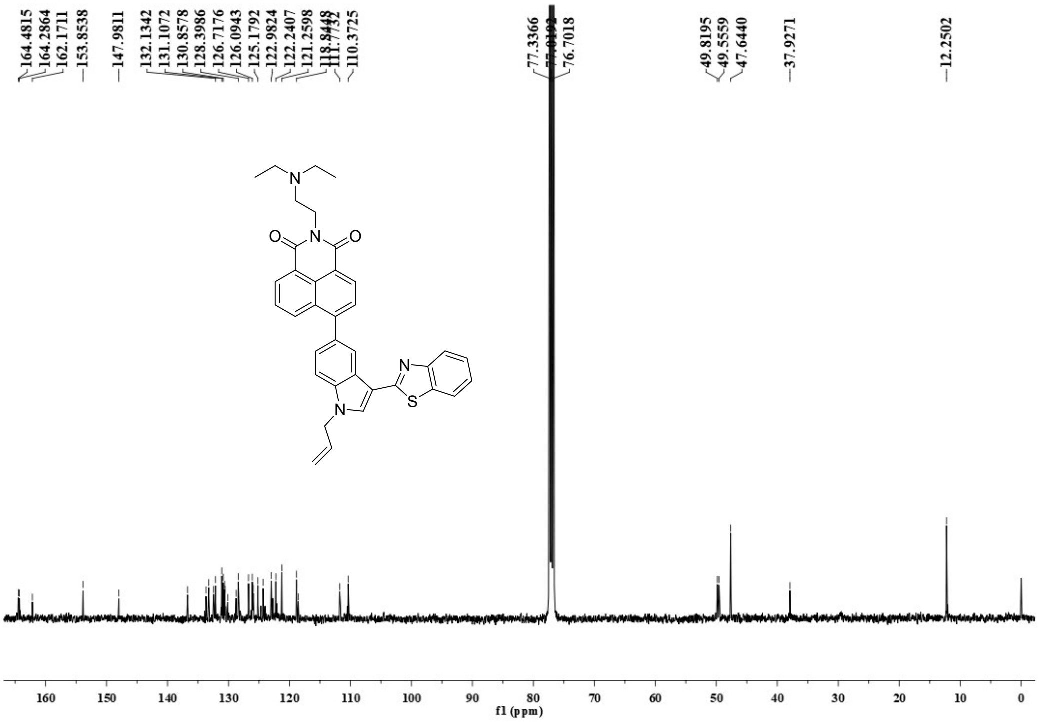

Figure S21: ^{13}C NMR spectrum of 6-(1-allyl-3-(benzo[*d*]thiazol-2-yl)-1*H*-indol-5-yl)-2-(2-(diethylamino)ethyl)-1*H*-benzo[*de*]isoquinoline-1,3(2*H*)-dione (**11**)

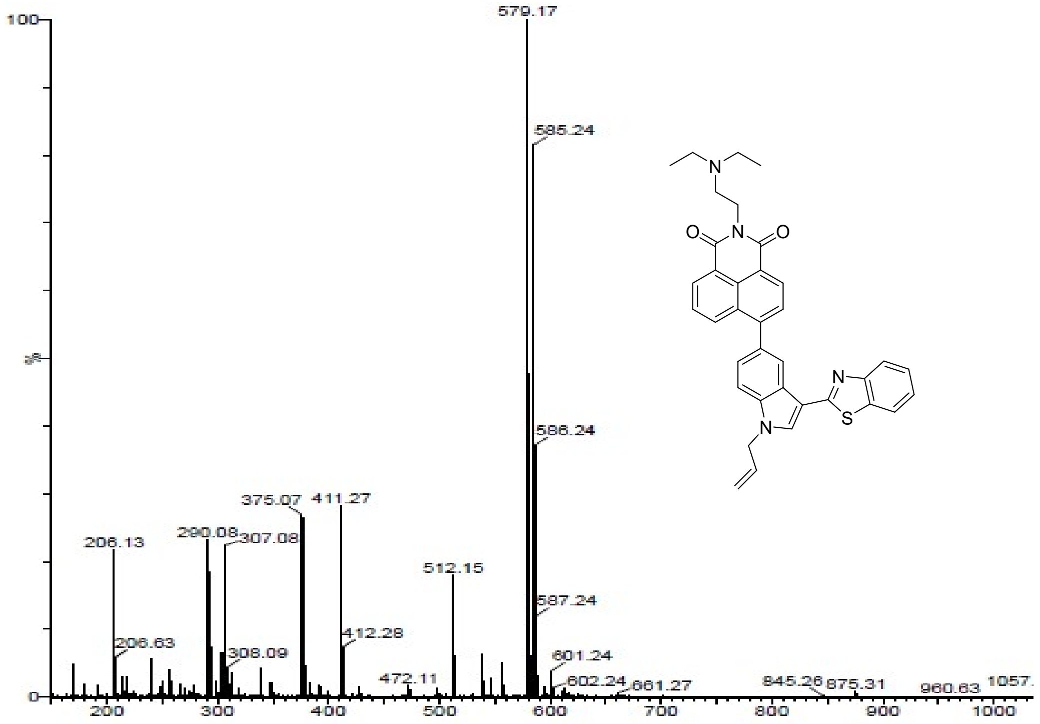

Figure S22: Mass spectrum of 6-(1-allyl-3-(benzo[*d*]thiazol-2-yl)-1*H*-indol-5-yl)-2-(2-(diethylamino)ethyl)-1*H*-benzo[*de*]isoquinoline-1,3(2*H*)-dione (**11**)

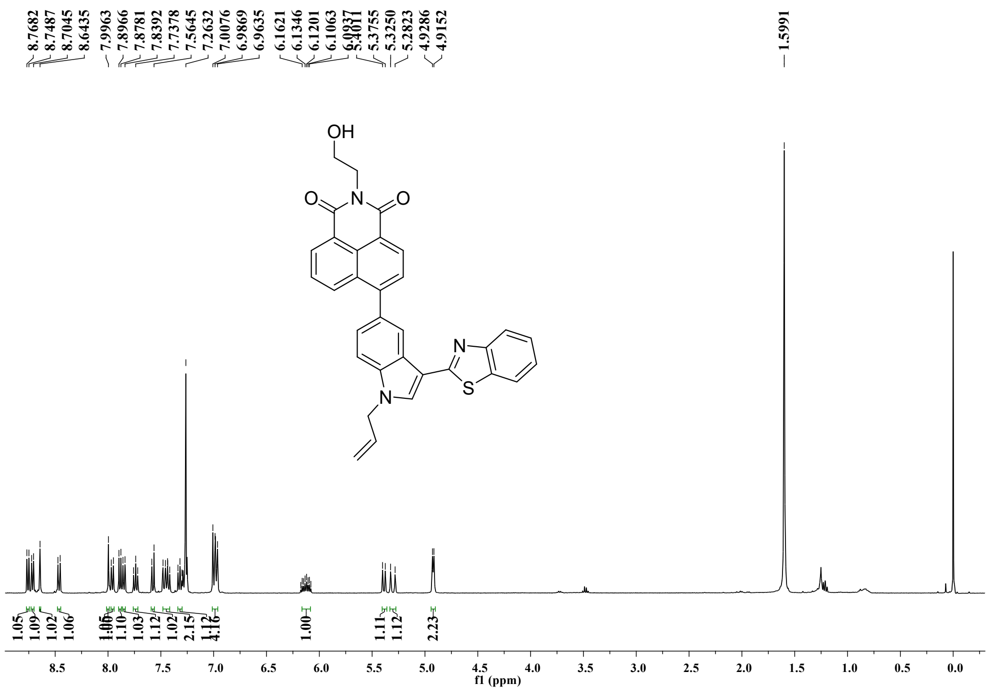

Figure S23: ^{1}H NMR spectrum of 6-(1-allyl-3-(benzo[*d*]thiazol-2-yl)-1*H*-indol-5-yl)-2-(2-hydroxyethyl)-1*H*-benzo[*de*]isoquinoline-1,3(2*H*)-dione (**12**)

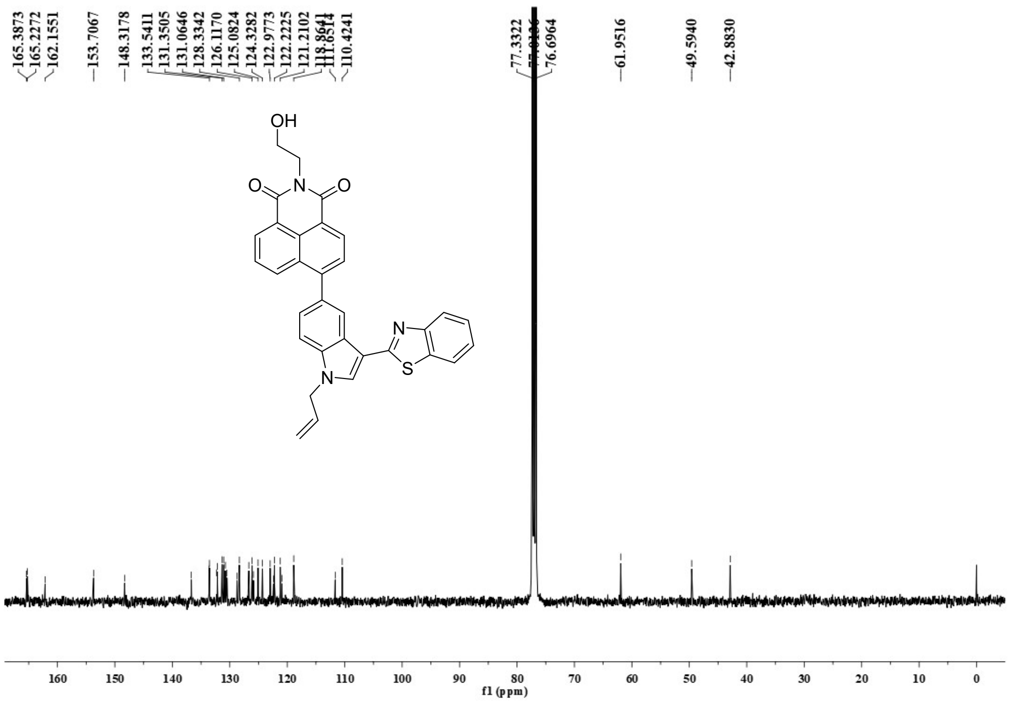

Figure S24: ^{13}C NMR spectrum of 6-(1-allyl-3-(benzo[*d*]thiazol-2-yl)-1*H*-indol-5-yl)-2-(2-hydroxyethyl)-1*H*-benzo[*de*]isoquinoline-1,3(2*H*)-dione (**12**)

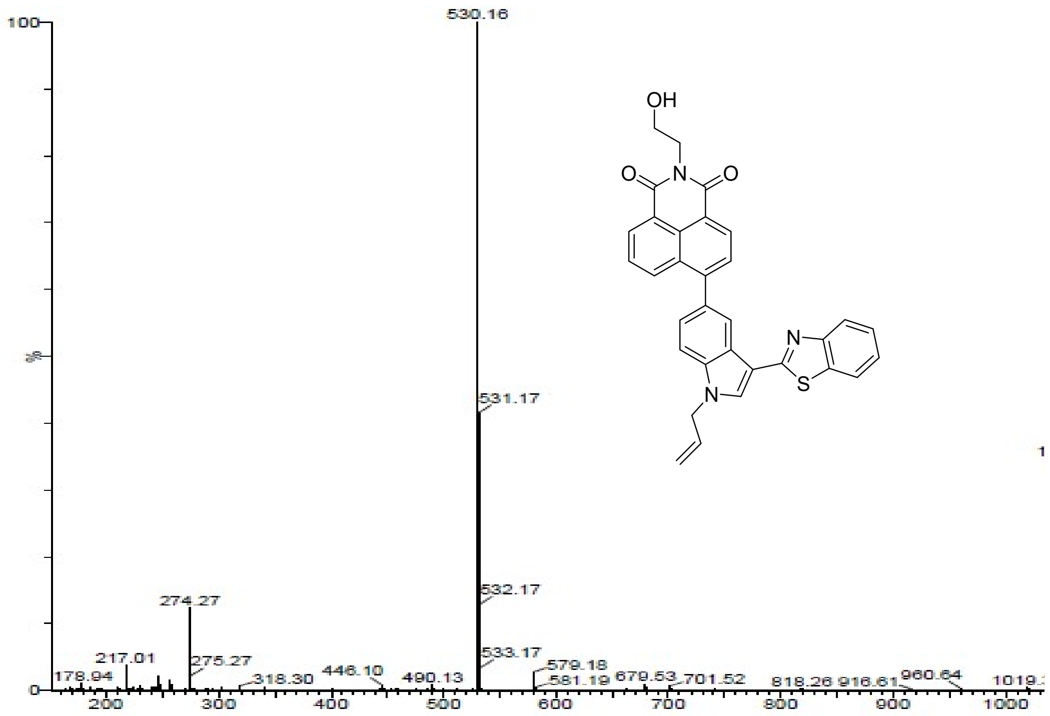

Figure S25: Mass spectrum of 6-(1-allyl-3-(benzo[*d*]thiazol-2-yl)-1*H*-indol-5-yl)-2-(2-hydroxyethyl)-1*H*-benzo[*de*]isoquinoline-1,3(2*H*)-dione (**12**)

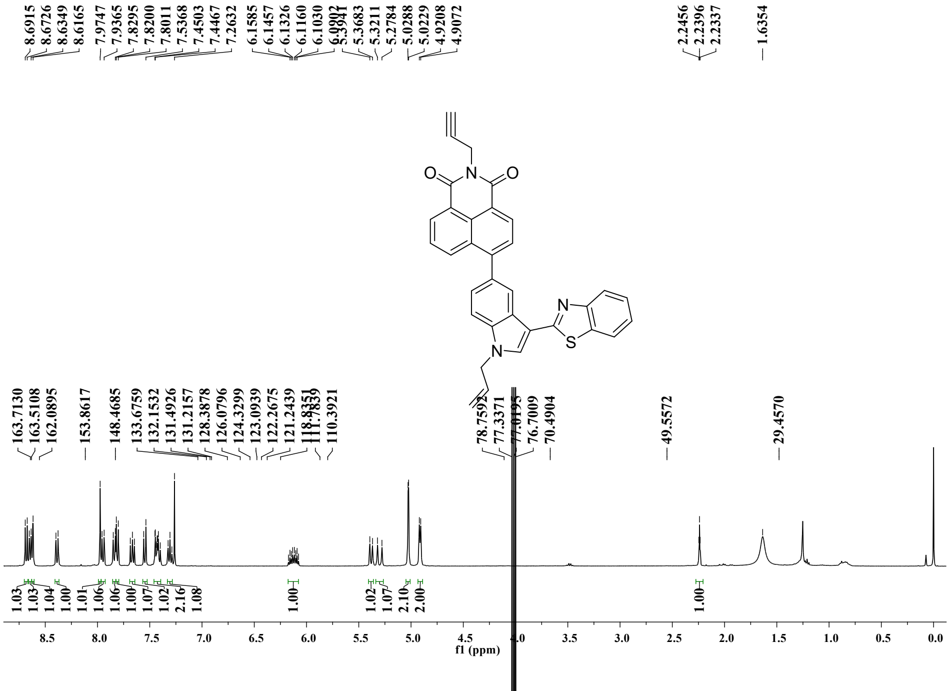

Figure S26: ^{1}H NMR spectrum of 6-(1-allyl-3-(benzo[*d*]thiazol-2-yl)-1*H*-indol-5-yl)-2-(prop-2-yn-1-yl)-1*H*-benzo[*de*]isoquinoline-1,3(2*H*)-dione (**13**)

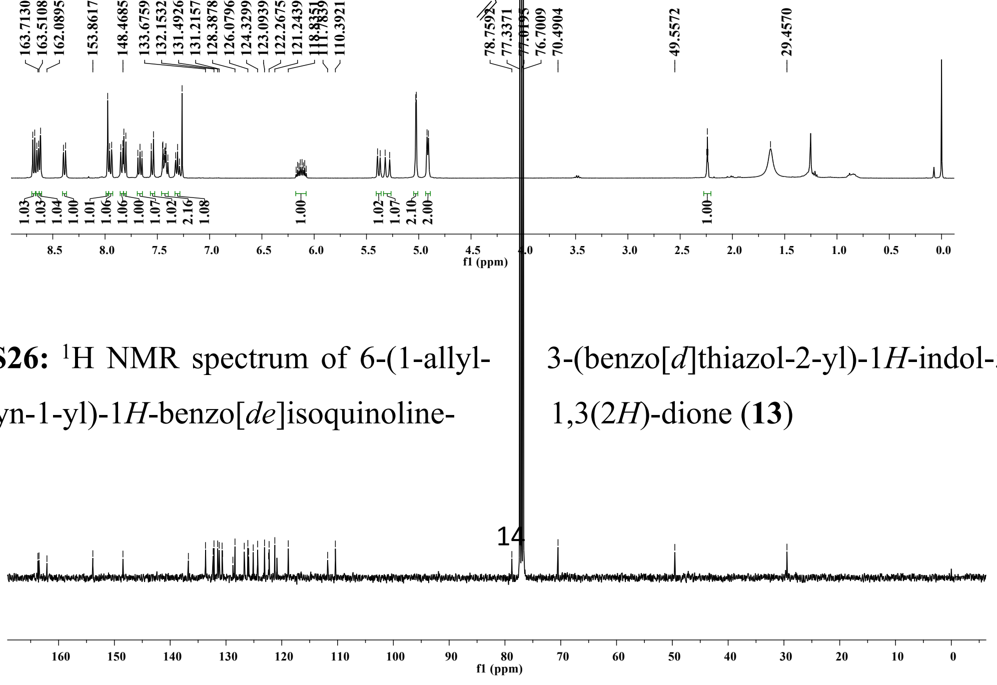

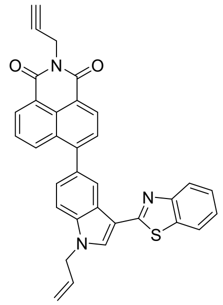

Figure S27: ^{13}C NMR spectrum of 6-(1-allyl-3-(benzo[*d*]thiazol-2-yl)-1*H*-indol-5-yl)-2-(prop-2-yn-1-yl)-1*H*-benzo[*de*]isoquinoline-1,3(2*H*)-dione (**13)**

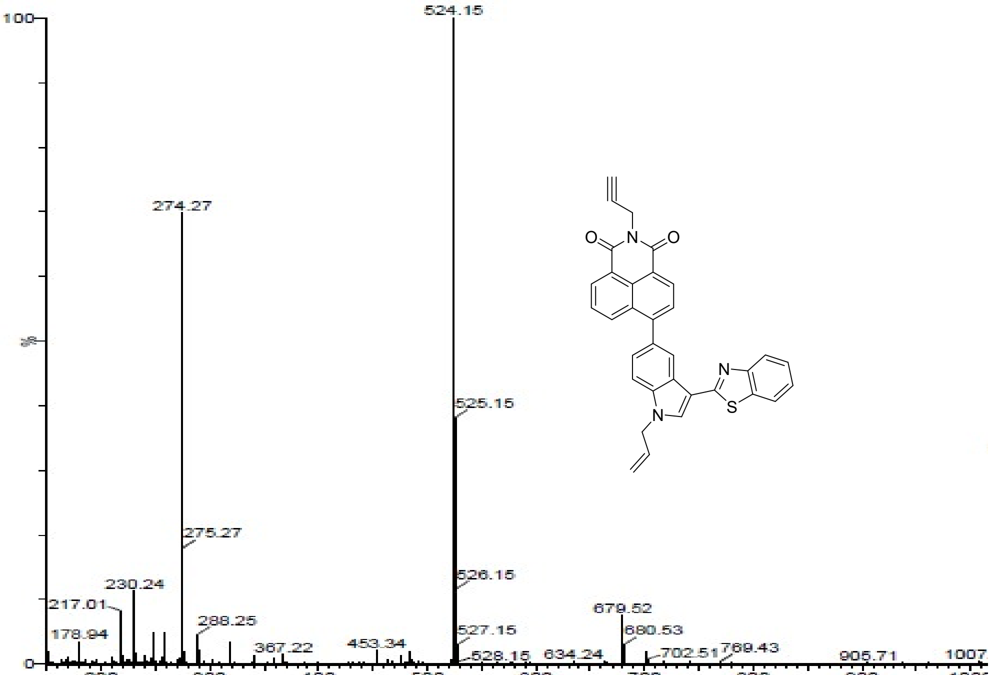

Figure S28: Mass spectrum of 6-(1-allyl-3-(benzo[*d*]thiazol-2-yl)-1*H*-indol-5-yl)-2-(prop-2-yn-1-yl)-1*H*-benzo[*de*]isoquinoline-1,3(2*H*)-dione (**13**)

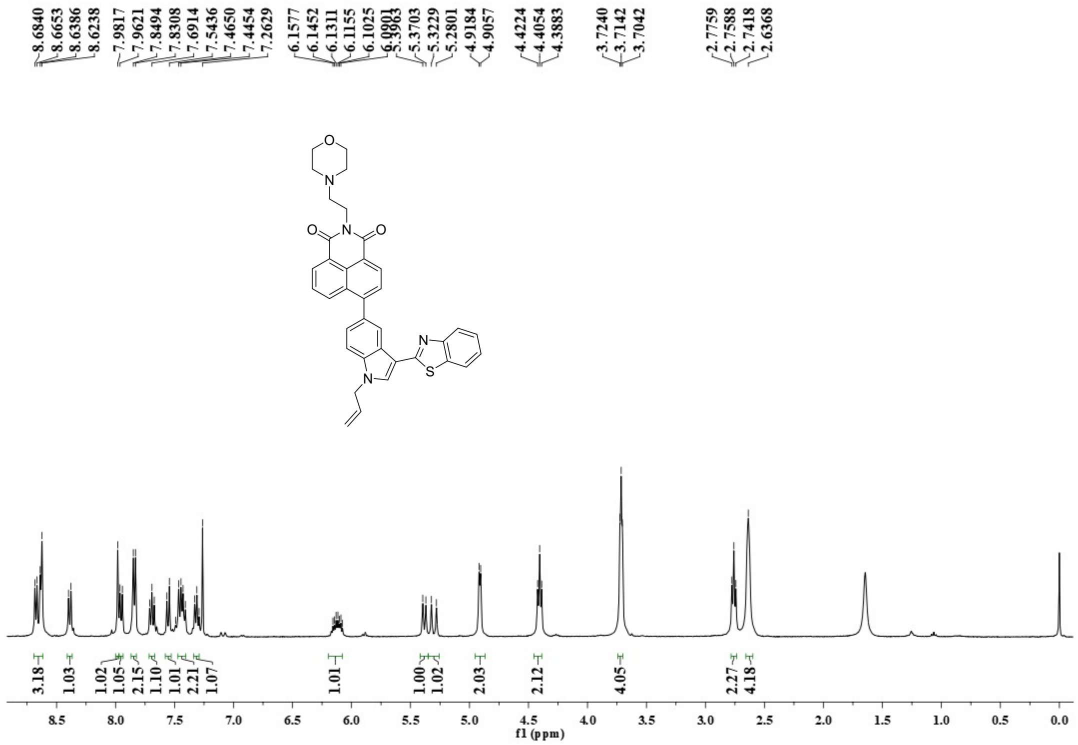

Figure S29: ^{1}H NMR spectrum of 6-(1-allyl-3-(benzo[*d*]thiazol-2-yl)-1*H*-indol-5-yl)-2-(2-morpholinoethyl)-1*H*-benzo[*de*]isoquinoline-1,3(2*H*)-dione (**14**)

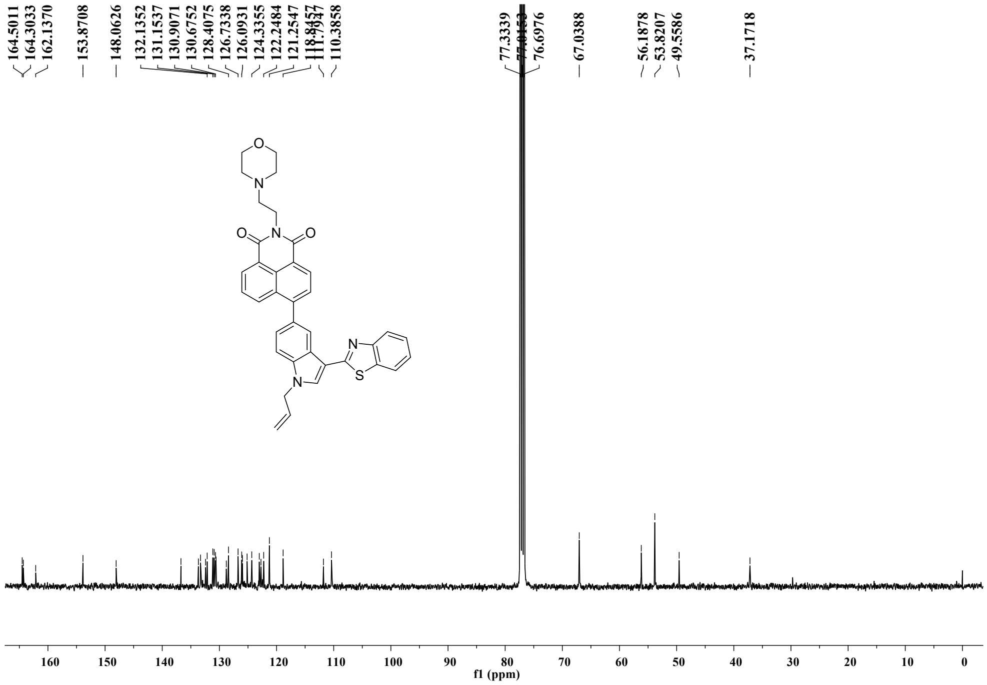

Figure S30: ^{13}C NMR spectrum of 6-(1-allyl-3-(benzo[*d*]thiazol-2-yl)-1*H*-indol-5-yl)-2-(2-morpholinoethyl)-1*H*-benzo[*de*]isoquinoline-1,3(2*H*)-dione (**14**)

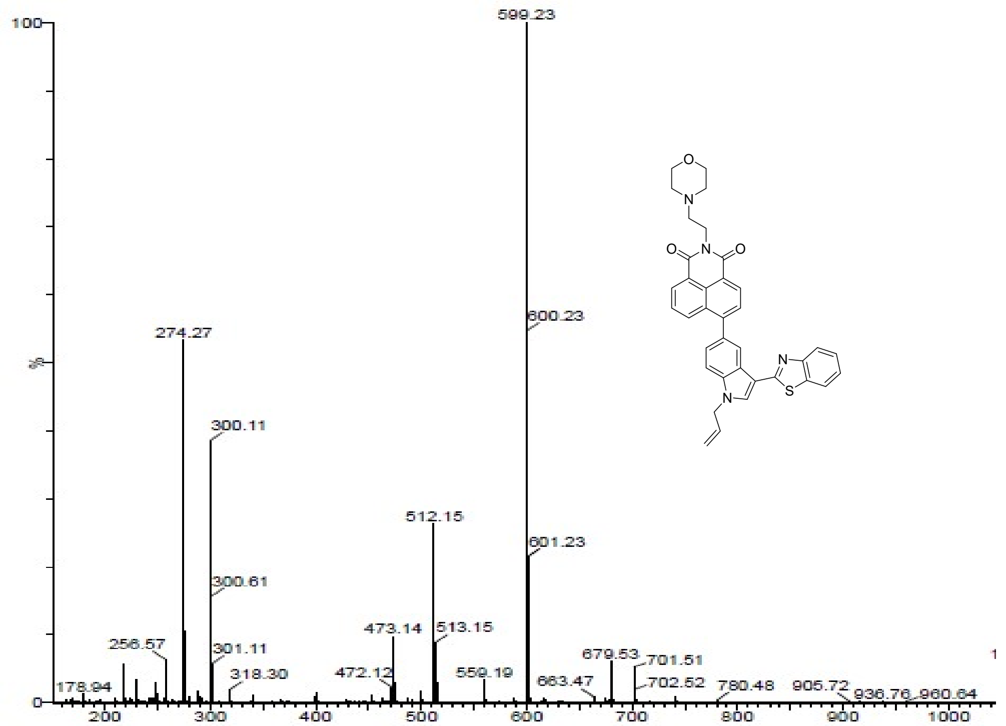

Figure S31: Mass spectrum of 6-(1-allyl-3-(benzo[*d*]thiazol-2-yl)-1*H*-indol-5-yl)-2-(2-morpholinoethyl)-1*H*-benzo[*de*]isoquinoline-1,3(2*H*)-dione (**14**)

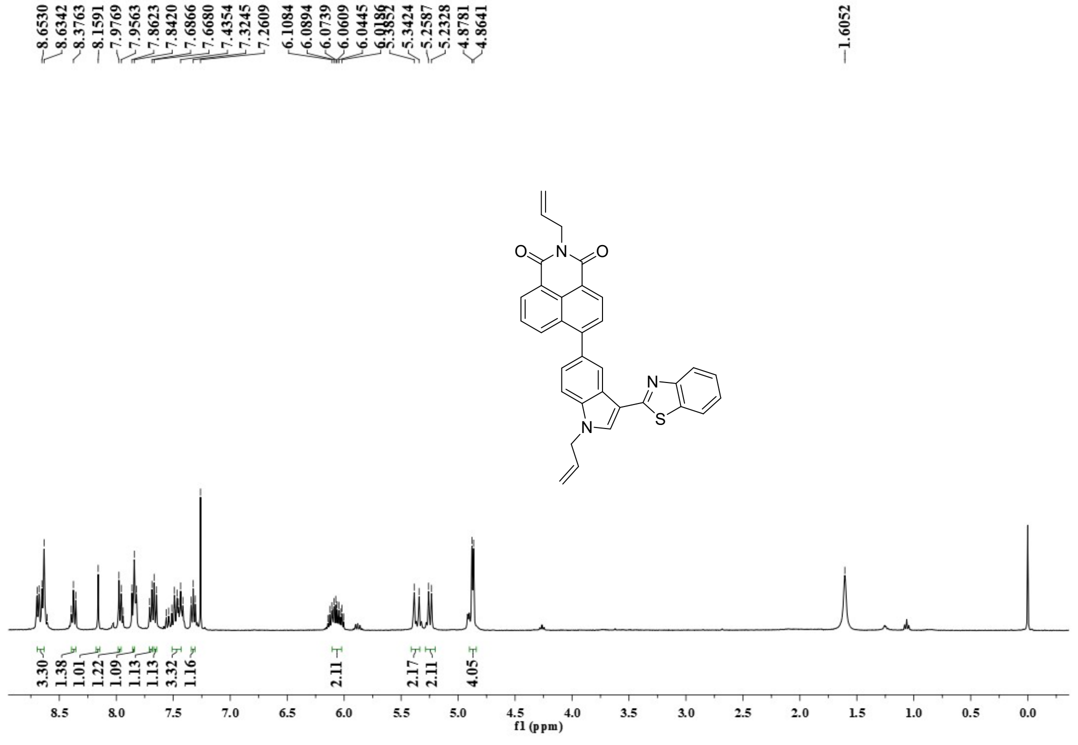

Figure S32: ^{1}H NMR spectrum of 2-allyl-6-(1-allyl-3-(benzo[*d*]thiazol-2-yl)-1*H*-indol-5-yl)-1*H*-benzo[*de*]isoquinoline-1,3(2*H*)-dione (**15**)

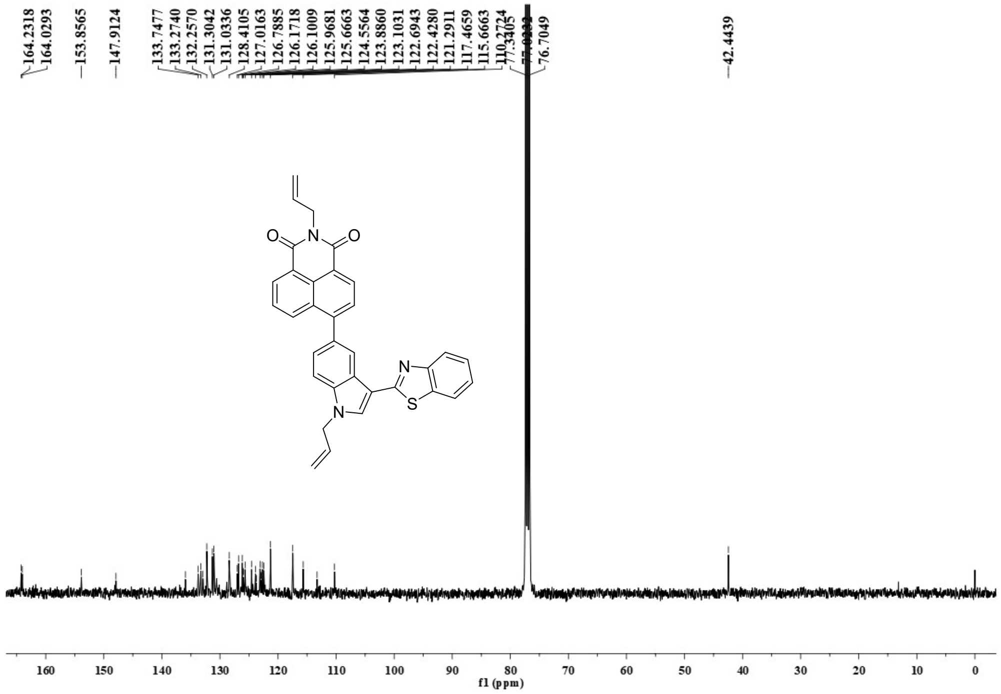

Figure S33: ^{13}C NMR spectrum of 2-allyl-6-(1-allyl-3-(benzo[*d*]thiazol-2-yl)-1*H*-indol-5-yl)-1*H*-benzo[*de*]isoquinoline-1,3(2*H*)-dione (**15**)

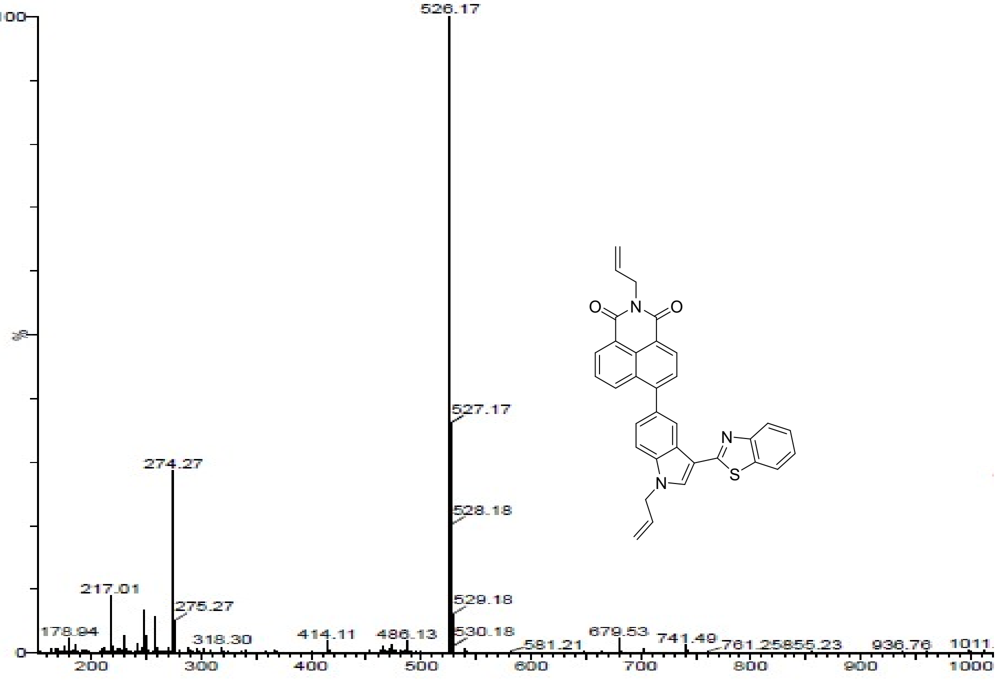

Figure S34: Mass spectrum of 2-allyl-6-(1-allyl-3-(benzo[*d*]thiazol-2-yl)-1*H*-indol-5-yl)-1*H*-benzo[*de*]isoquinoline-1,3(2*H*)-dione (**15**)

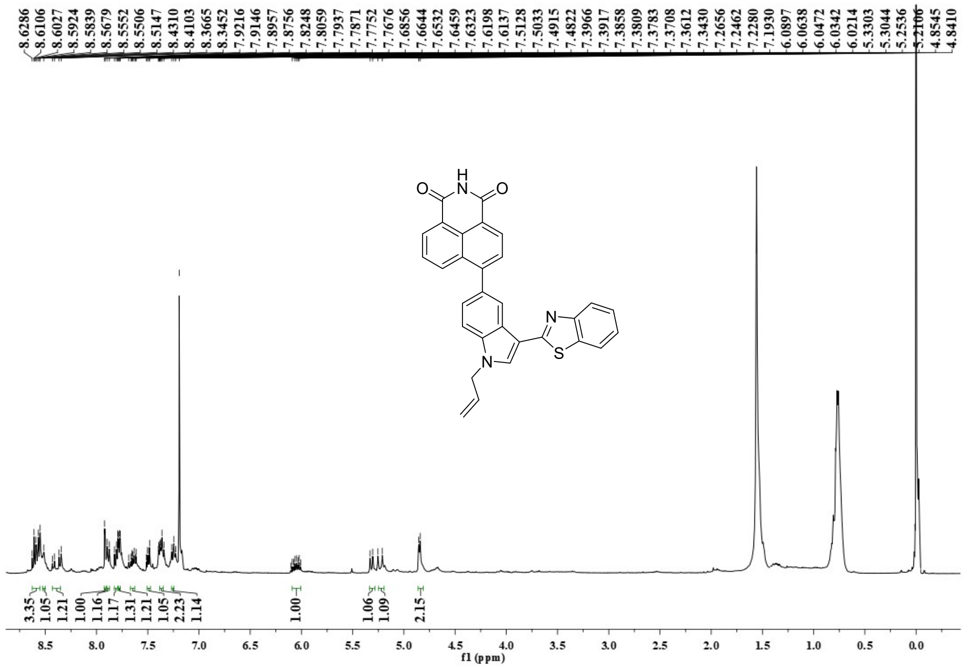

Figure S35: ^{1}H NMR spectrum of 6-(1-allyl-3-(benzo[*d*]thiazol-2-yl)-1*H*-indol-5-yl)-1*H*-benzo[*de*]isoquinoline-1,3(2*H*)-dione (**16**)

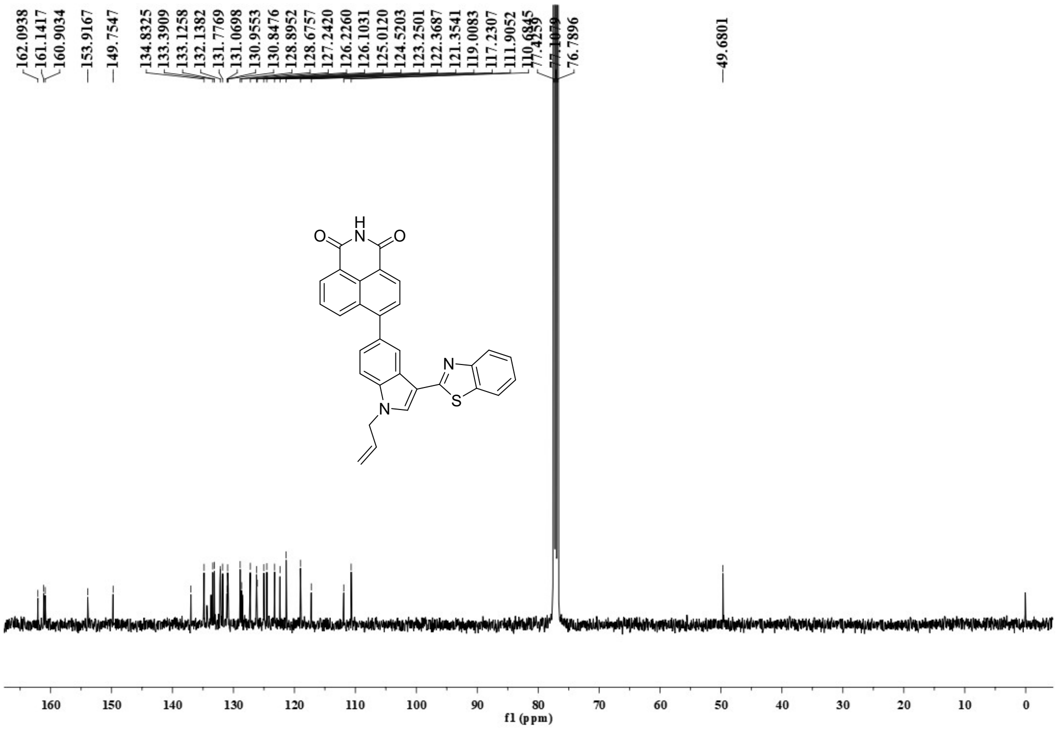

Figure S36: ^{13}C NMR spectrum of 6-(1-allyl-3-(benzo[*d*]thiazol-2-yl)-1*H*-indol-5-yl)-1*H*-benzo[*de*]isoquinoline-1,3(2*H*)-dione (**16**)

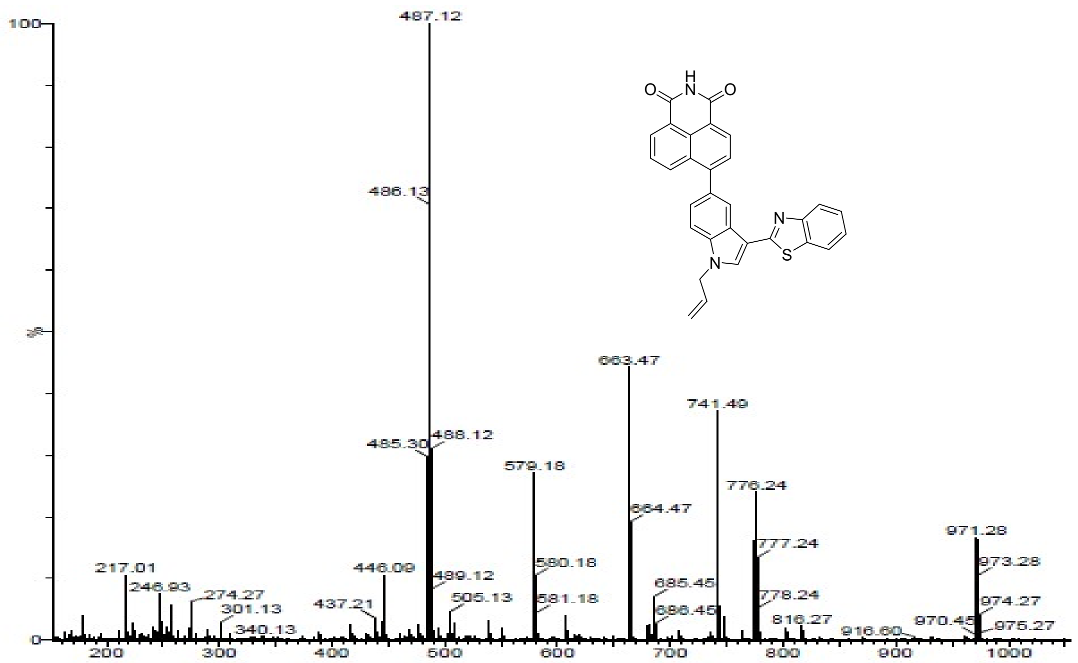

Figure S37: Mass spectrum of 6-(1-allyl-3-(benzo[*d*]thiazol-2-yl)-1*H*-indol-5-yl)-1*H*-benzo[*de*]isoquinoline-1,3(2*H*)-dione (**16**)

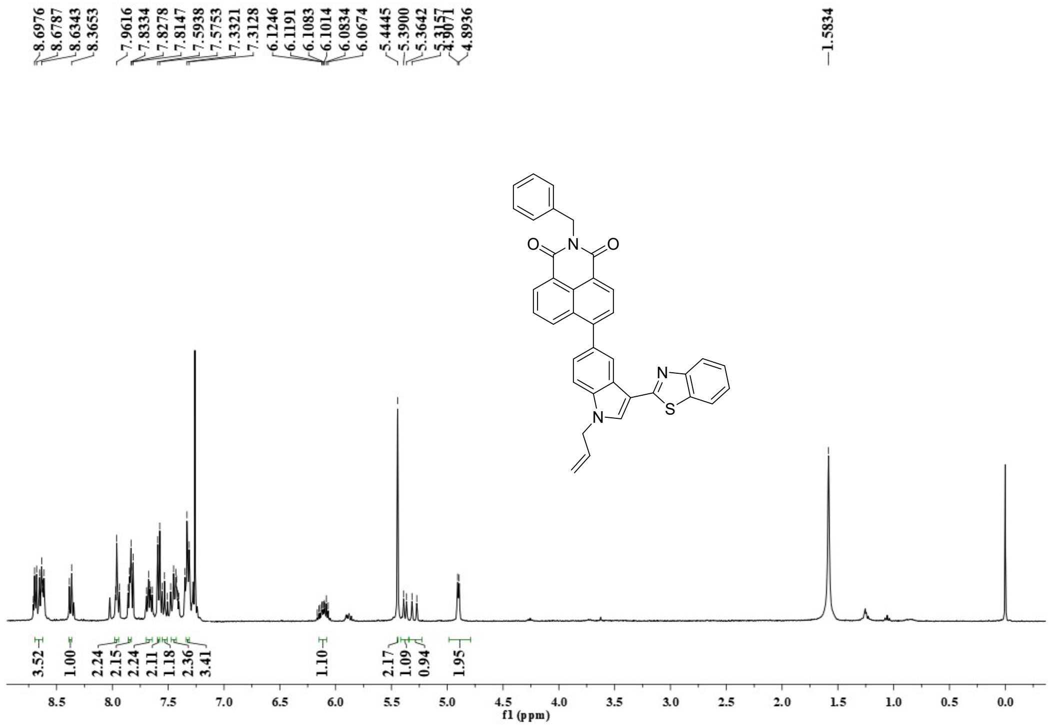

Figure S38: ^{1}H NMR spectrum of 6-(1-allyl-3-(benzo[*d*]thiazol-2-yl)-1*H*-indol-5-yl)-2-benzyl-1*H*-benzo[*de*]isoquinoline-1,3(2*H*)-dione (**17**)

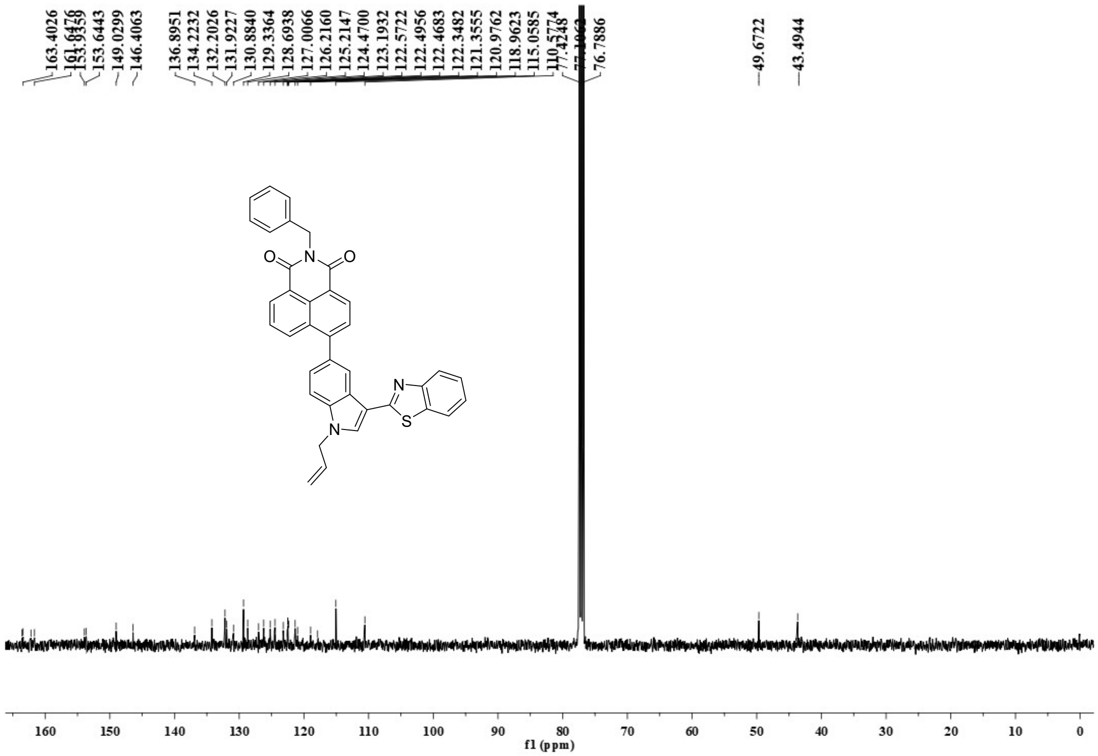

Figure S39: ^{13}C NMR spectrum of 6-(1-allyl-3-(benzo[*d*]thiazol-2-yl)-1*H*-indol-5-yl)-2-benzyl-1*H*-benzo[*de*]isoquinoline-1,3(2*H*)-dione (**17**)

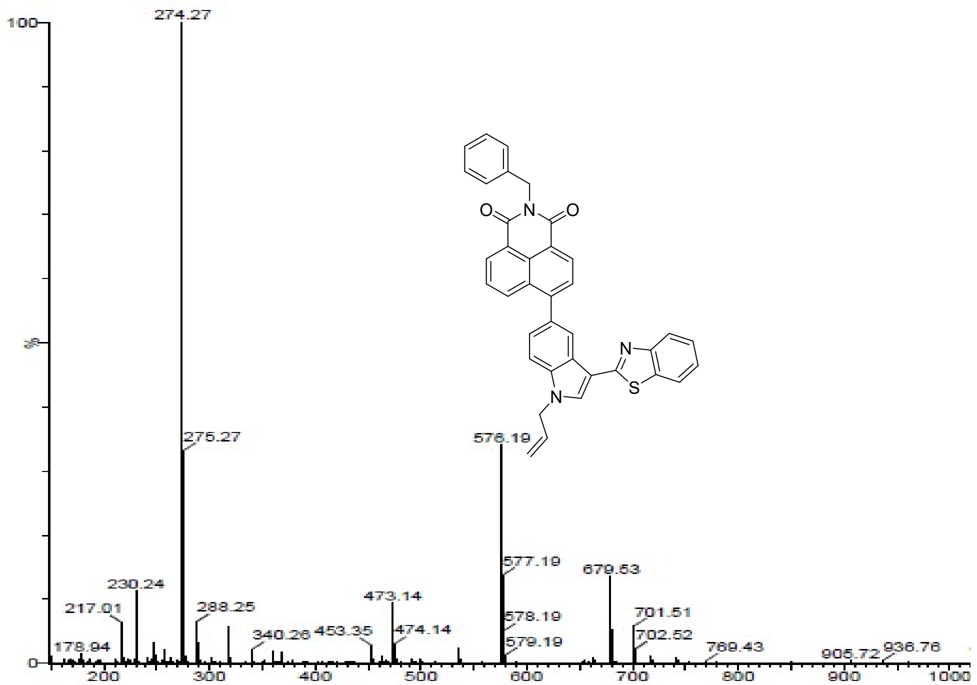

Figure S40: Mass spectrum of 6-(1-allyl-3-(benzo[*d*]thiazol-2-yl)-1*H*-indol-5-yl)-2-benzyl-1*H*-benzo[*de*]isoquinoline-1,3(2*H*)-dione (**17**)

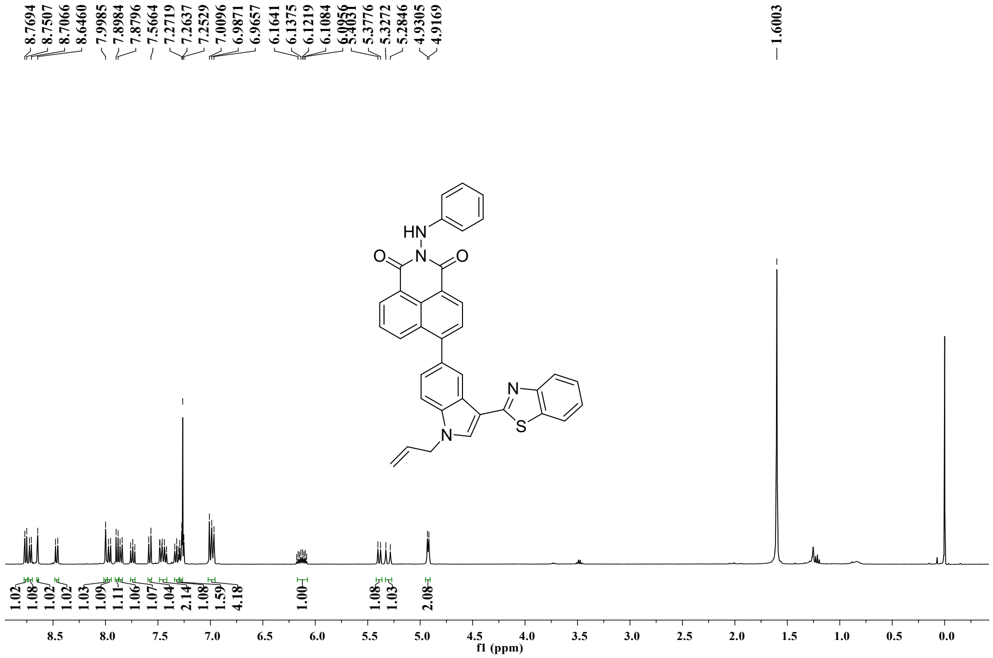

Figure S41: ^{1}H NMR spectrum of 6-(1-allyl-3-(benzo[*d*]thiazol-2-yl)-1*H*-indol-5-yl)-2-(phenylamino)-1*H*-benzo[*de*]isoquinoline-1,3(2*H*)-dione (**18**)

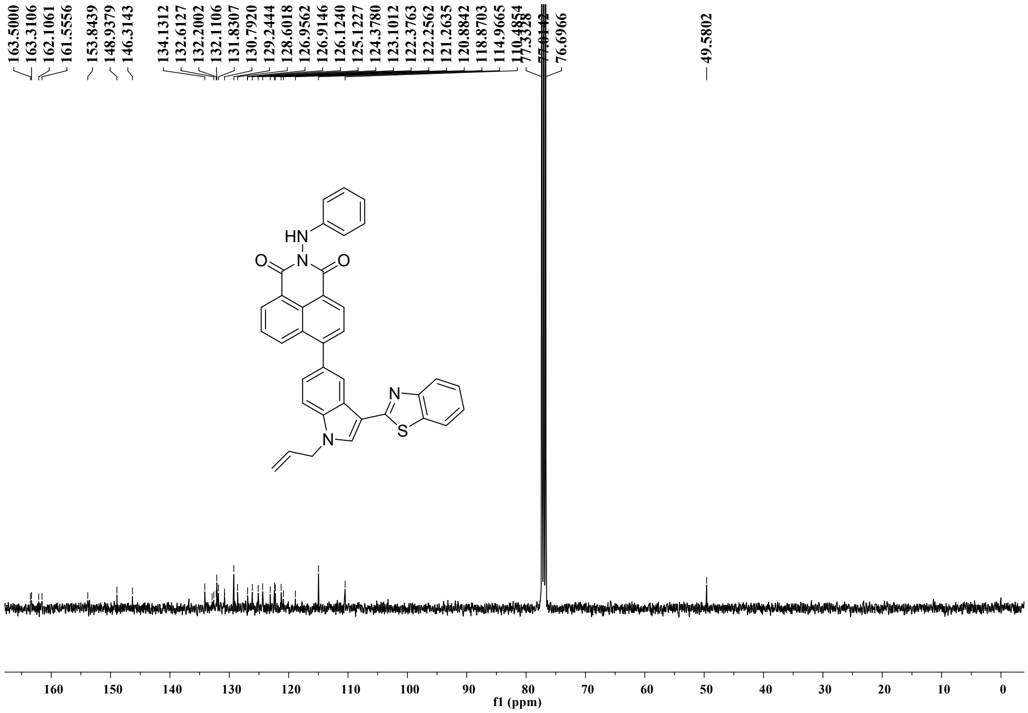

Figure S42: ^{13}C NMR spectrum of 6-(1-allyl-3-(benzo[*d*]thiazol-2-yl)-1*H*-indol-5-yl)-2-(phenylamino)-1*H*-benzo[*de*]isoquinoline-1,3(2*H*)-dione (**18**)

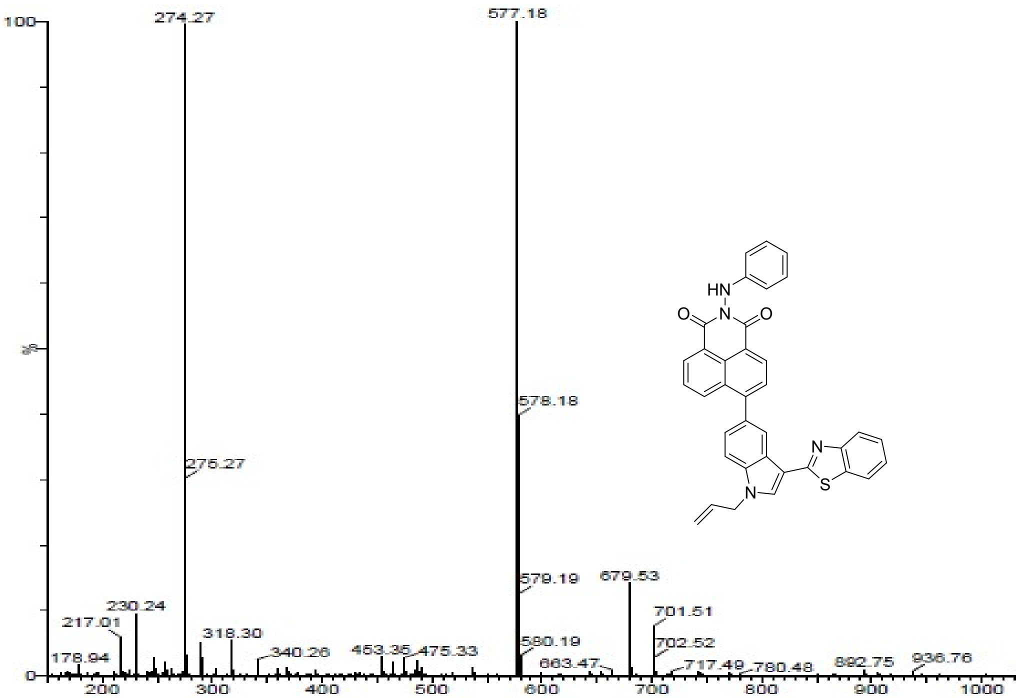

Figure S43: Mass spectrum of 6-(1-allyl-3-(benzo[*d*]thiazol-2-yl)-1*H*-indol-5-yl)-2-(phenylamino)-1*H*-benzo[*de*]isoquinoline-1,3(2*H*)-dione (**18**)

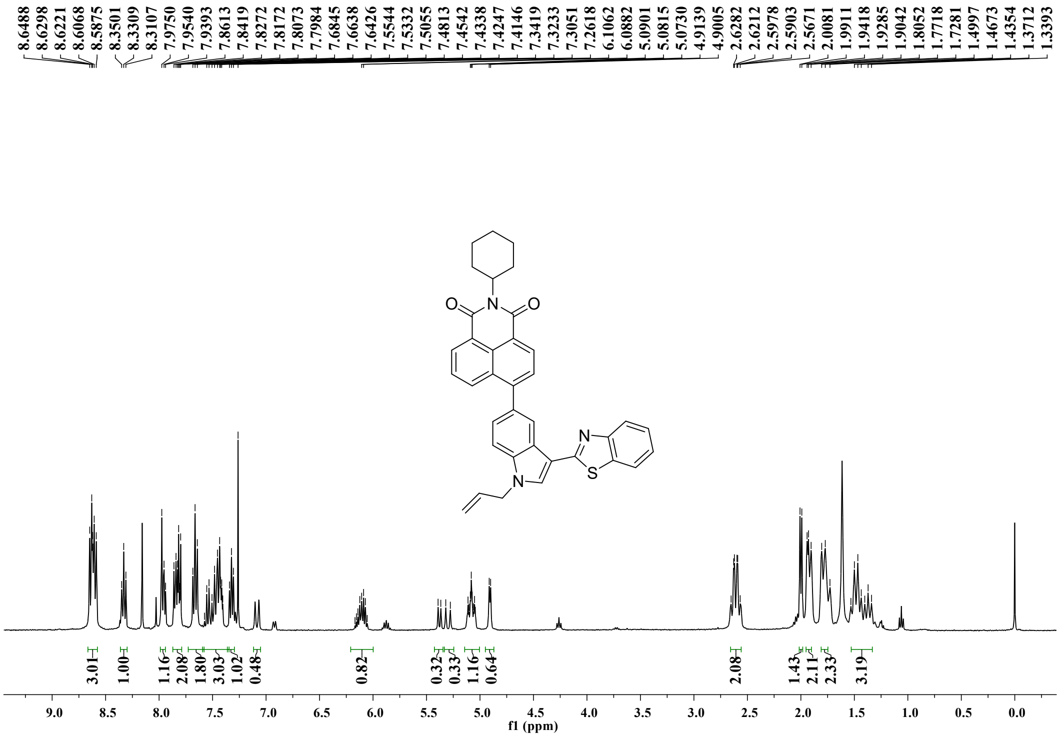

Figure S44: ^{1}H NMR spectrum of 6-(1-allyl-3-(benzo[*d*]thiazol-2-yl)-1*H*-indol-5-yl)-2-cyclohexyl-1*H*-benzo[*de*]isoquinoline-1,3(2*H*)-dione (**19**)

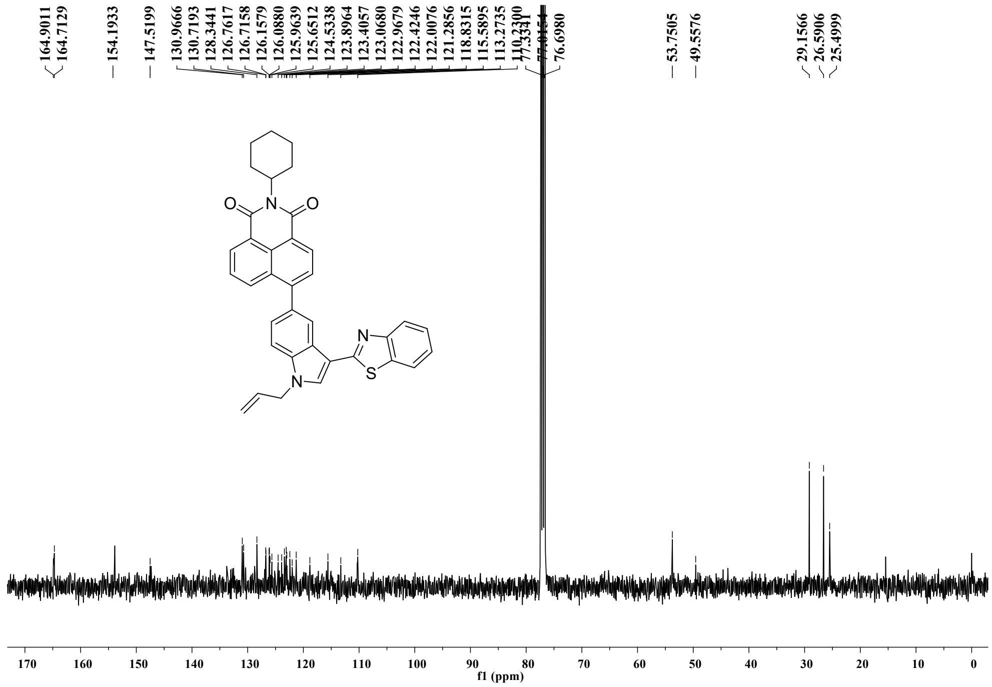

Figure S45: ^{13}C NMR spectrum of 6-(1-allyl-3-(benzo[*d*]thiazol-2-yl)-1*H*-indol-5-yl)-2-cyclohexyl-1*H*-benzo[*de*]isoquinoline-1,3(2*H*)-dione (**19**)

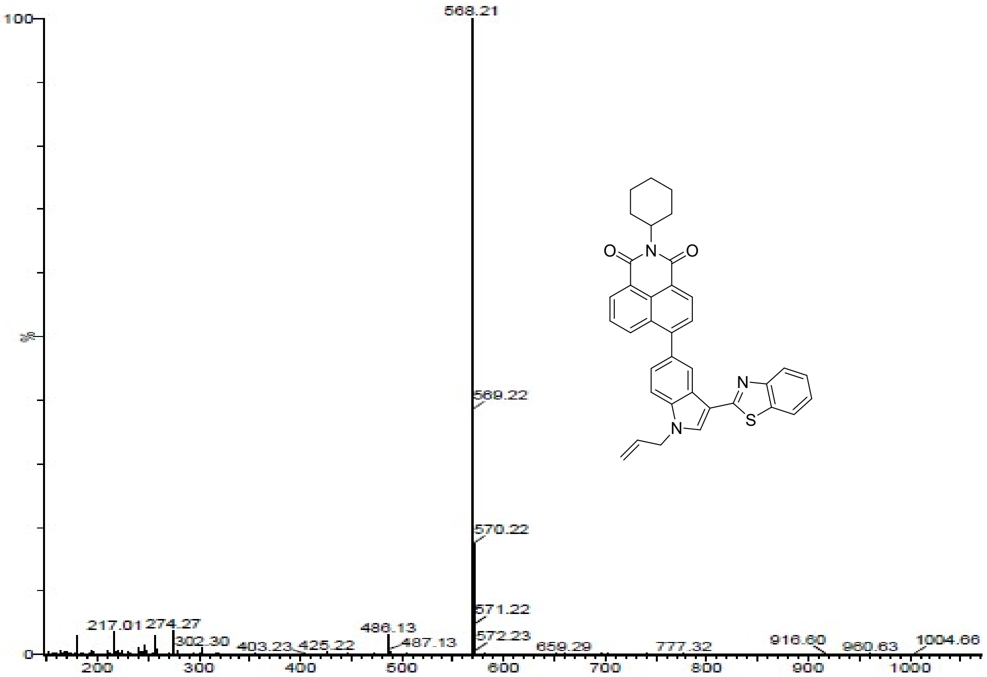

Figure S46: Mass spectrum of 6-(1-allyl-3-(benzo[*d*]thiazol-2-yl)-1*H*-indol-5-yl)-2-cyclohexyl-1*H*-benzo[*de*]isoquinoline-1,3(2*H*)-dione (**19**)

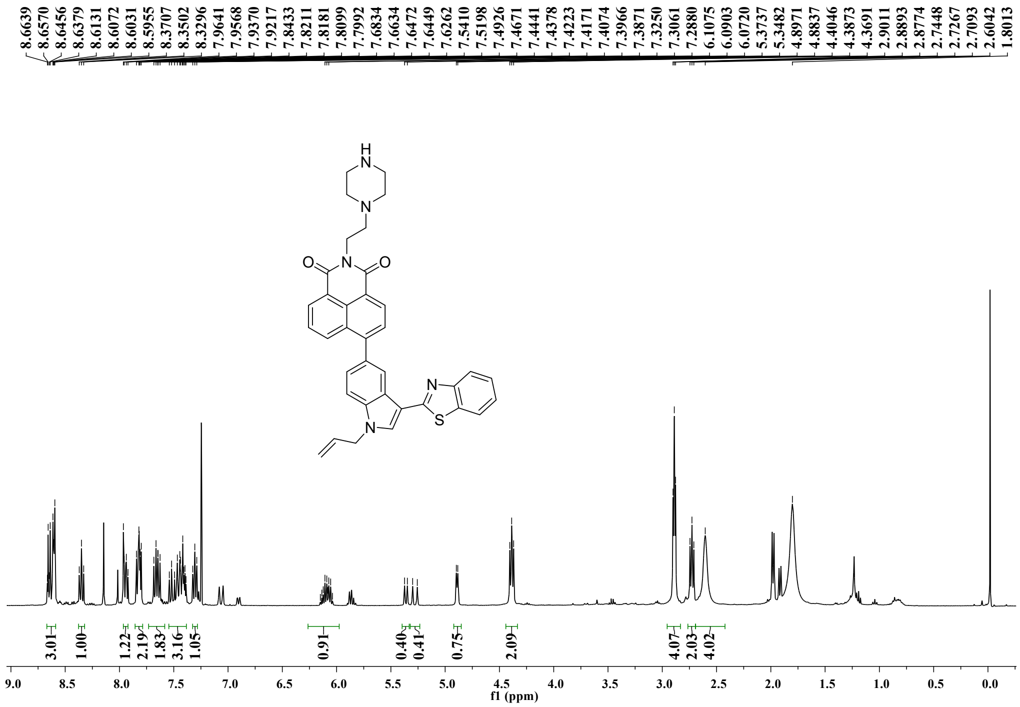

Figure S47: ^{1}H NMR spectrum of 6-(1-allyl-3-(benzo[*d*]thiazol-2-yl)-1*H*-indol-5-yl)-2-(2-(piperazin-1-yl)ethyl)-1*H*-benzo[*de*]isoquinoline-1,3(2*H*)-dione (**20**)

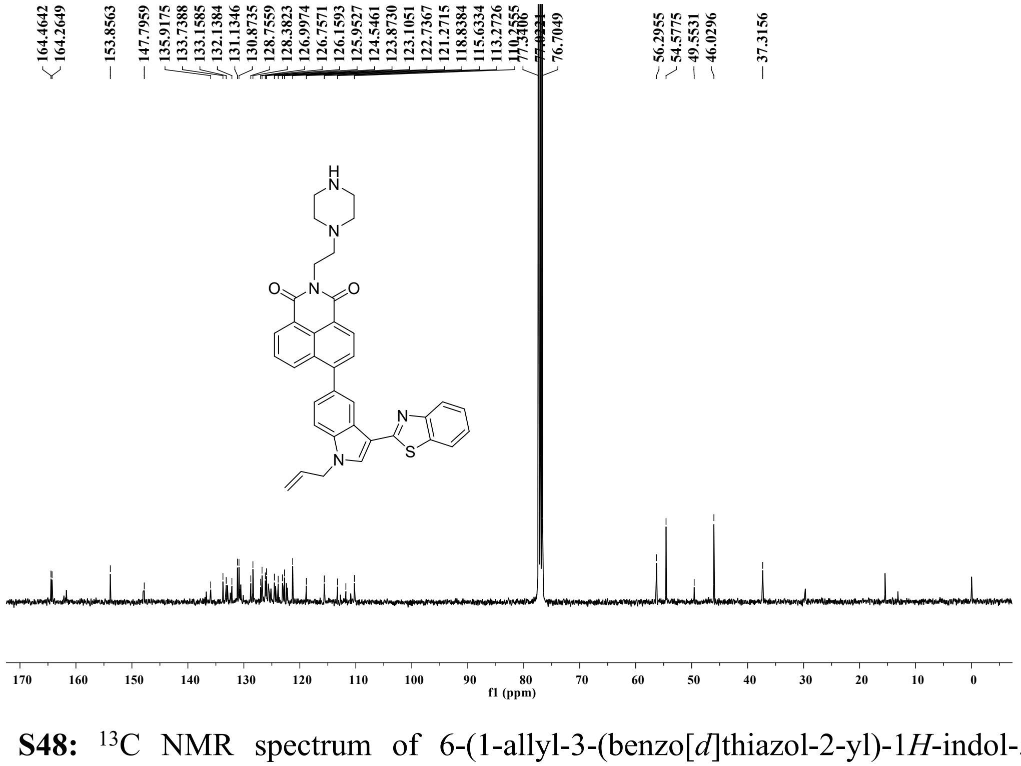

Figure S48: ^{13}C NMR spectrum of 6-(1-allyl-3-(benzo[*d*]thiazol-2-yl)-1*H*-indol-5-yl)-2-(2-(piperazin-1-yl)ethyl)-1*H*-benzo[*de*]isoquinoline-1,3(2*H*)-dione (**20**)

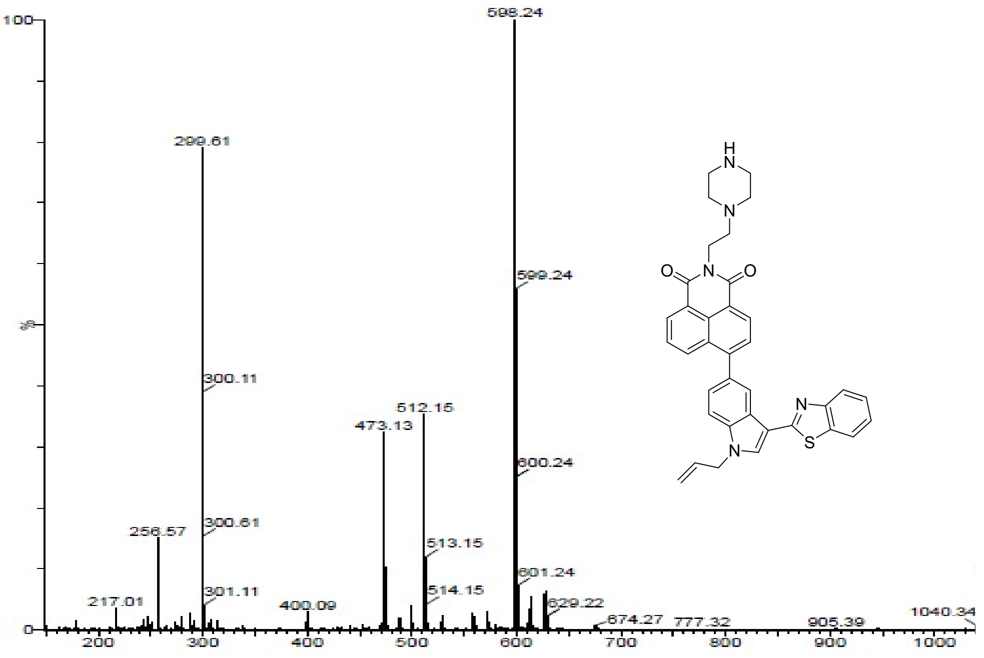

Figure S49: Mass spectrum of 6-(1-allyl-3-(benzo[*d*]thiazol-2-yl)-1*H*-indol-5-yl)-2-(2-(piperazin-1-yl)ethyl)-1*H*-benzo[*de*]isoquinoline-1,3(2*H*)-dione (**20**)

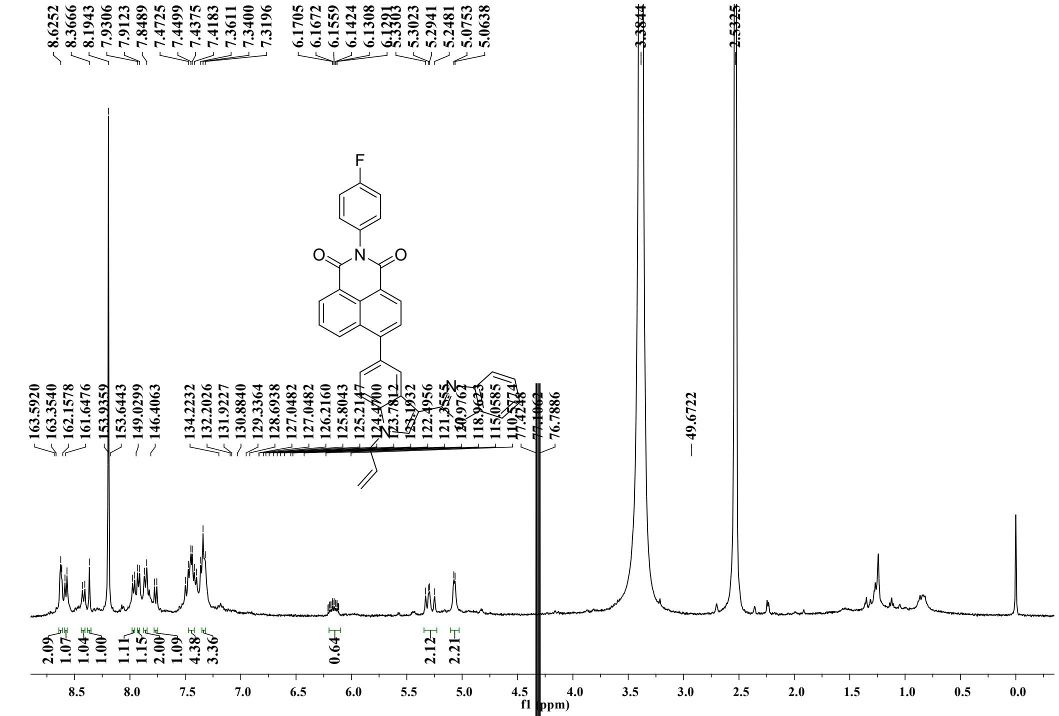

Figure S50: ^{1}H NMR spectrum of 6-(3-(benzo[*d*]thiazol-2-yl)-1-propyl-1*H*-indol-5-yl)-2-(4-fluorophenyl)-1*H*-benzo[*de*]isoquinoline-1,3(2*H*)-dione (**21**)

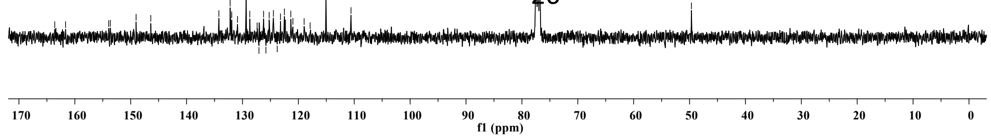

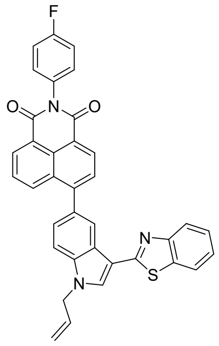

Figure S51: ^{13}C NMR spectrum of 6-(3-(benzo[*d*]thiazol-2-yl)-1-propyl-1*H*-indol-5-yl)-2-(4-fluorophenyl)-1*H*-benzo[*de*]isoquinoline-1,3(2*H*)-dione (**21**)

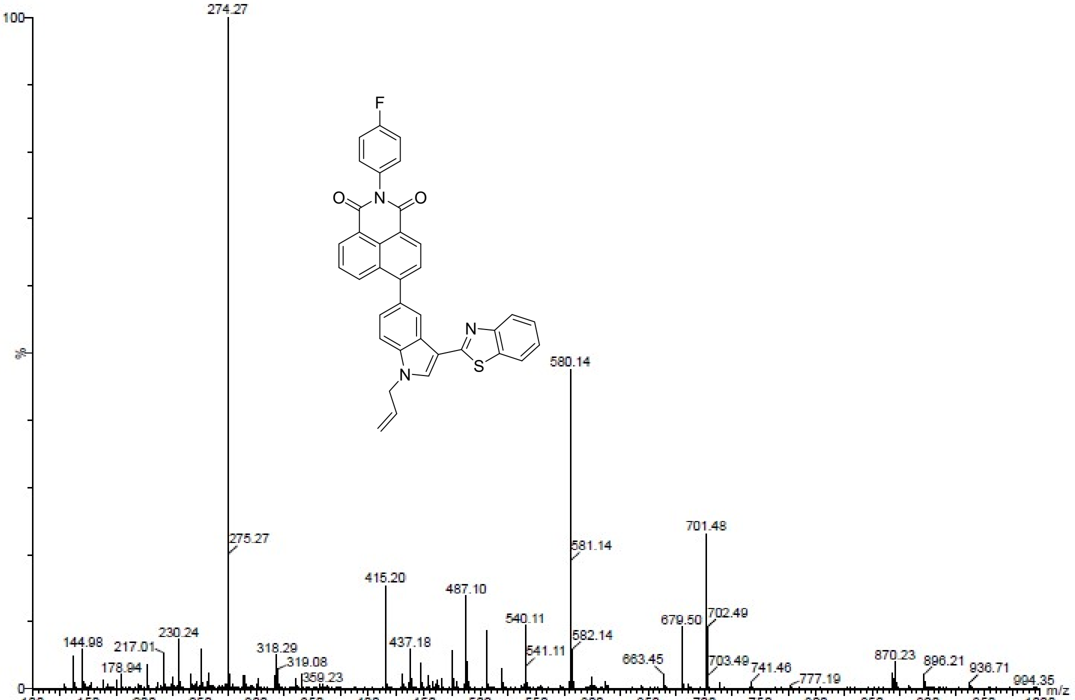

Figure S52: Mass spectrum of 6-(3-(benzo[*d*]thiazol-2-yl)-1-propyl-1*H*-indol-5-yl)-2-(4-fluorophenyl)-1*H*-benzo[*de*]isoquinoline-1,3(2*H*)-dione (**21**)

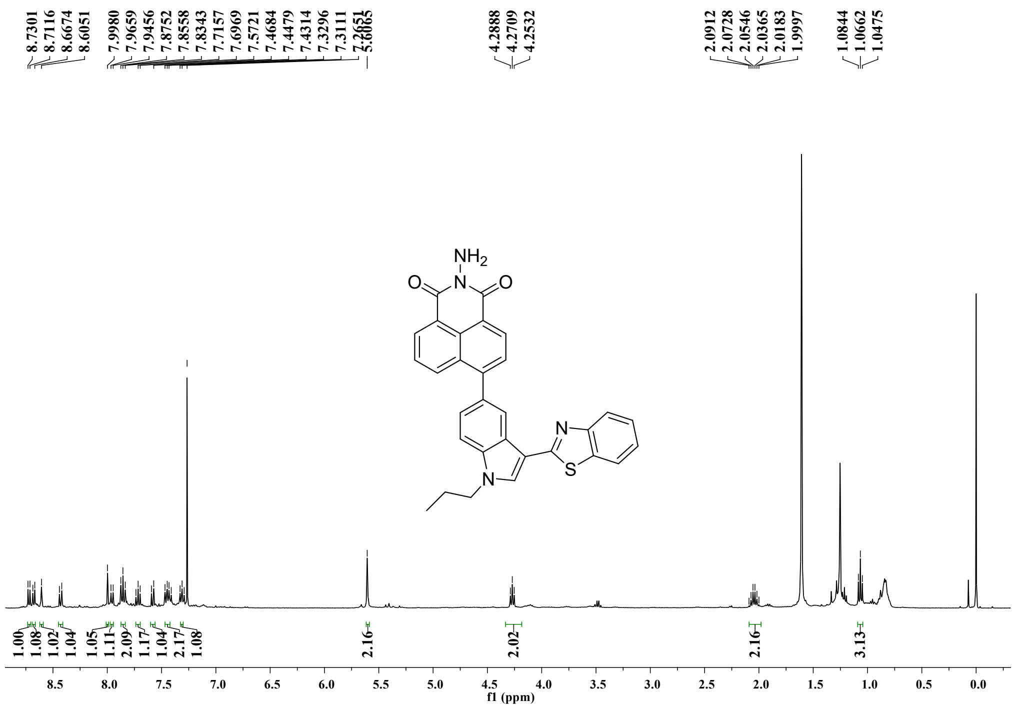

Figure S53: ^{1}H NMR spectrum of 2-amino-6-(3-(benzo[*d*]thiazol-2-yl)-1-propyl-1*H*-indol-5-yl)-1*H*-benzo[*de*]isoquinoline-1,3(2*H*)-dione **(22)**

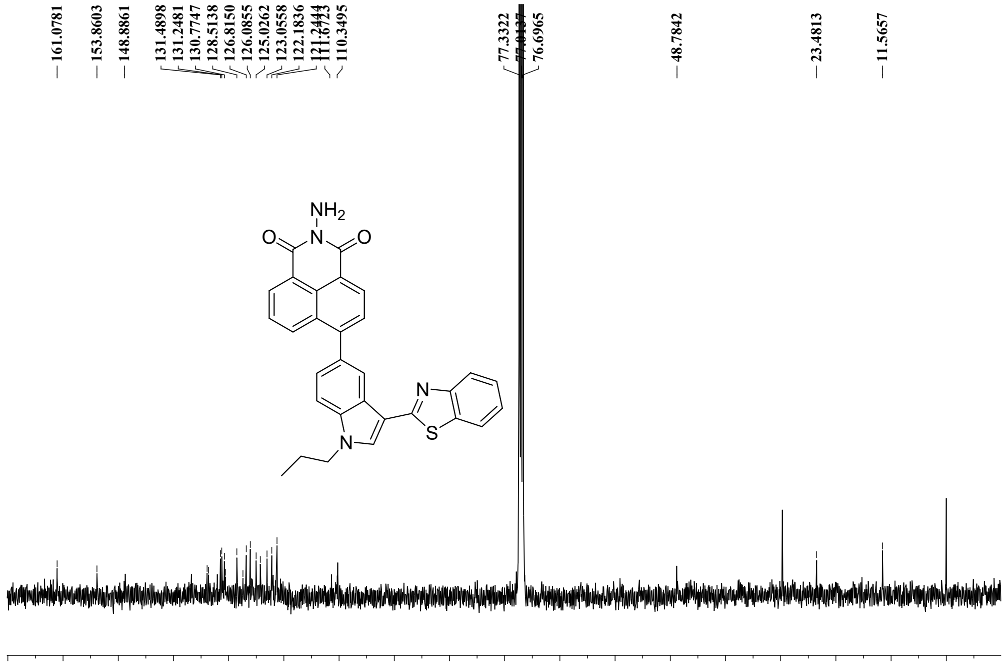

Figure S54: ^{13}C NMR spectrum of 2-amino-6-(3-(benzo[*d*]thiazol-2-yl)-1-propyl-1*H*-indol-5-yl)-1*H*-benzo[*de*]isoquinoline-1,3(2*H*)-dione **(22)**

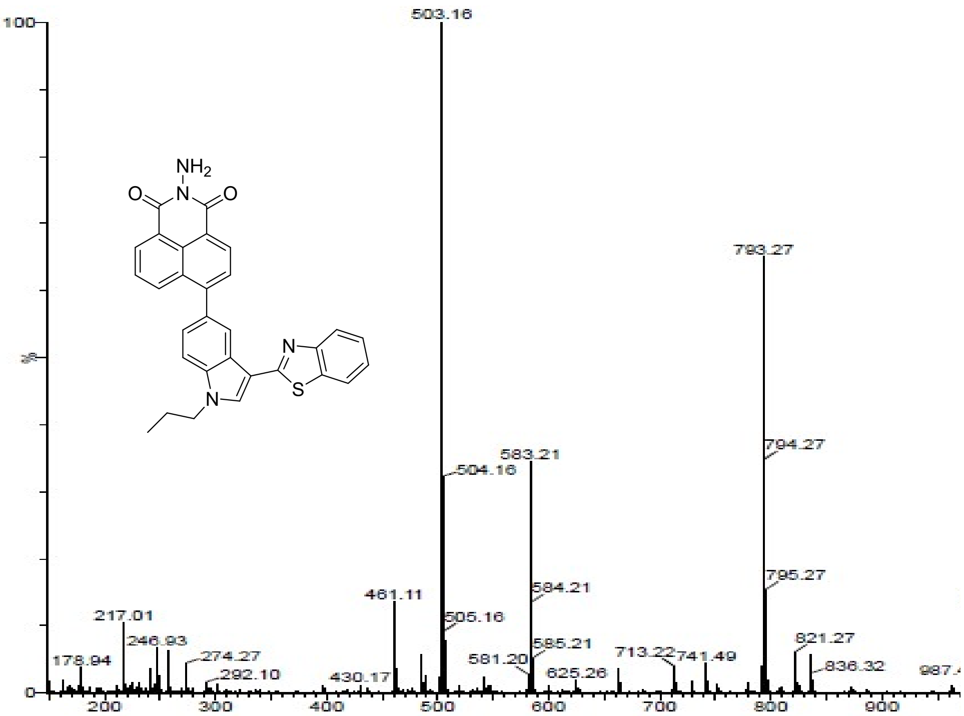

Figure S55: Mass spectrum of 2-amino-6-(3-(benzo[*d*]thiazol-2-yl)-1-propyl-1*H*-indol-5-yl)-1*H*-benzo[*de*]isoquinoline-1,3(2*H*)-dione **(22)**

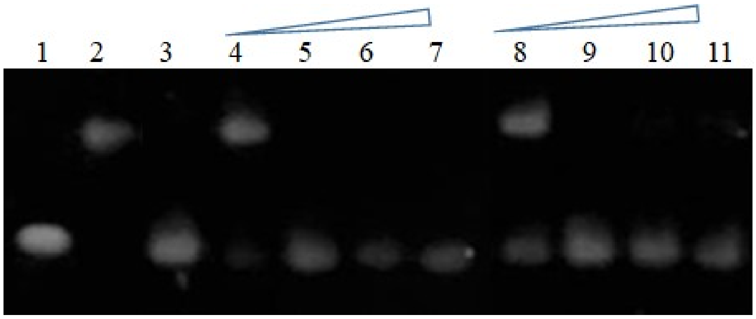

Figure S56. Agarose gel stained with ethidium bromide for inhibitoty activity towards Topo IIα relaxation by compounds **12** and **13**. Lane 1: pHOT1 plasmid DNA, lane 2: pHOT1 plasmid DNA + TOPO II, lane 3: plasmid DNA + TOPO II + etoposide (25 μM as positive control), lane 4-7 (compound **12**) and lane 8-11 (compound **13**): inhibition of relaxation of plasmid DNA by Topo IIα in the presence of 1**,** 5, 10, and 50 μM compound.

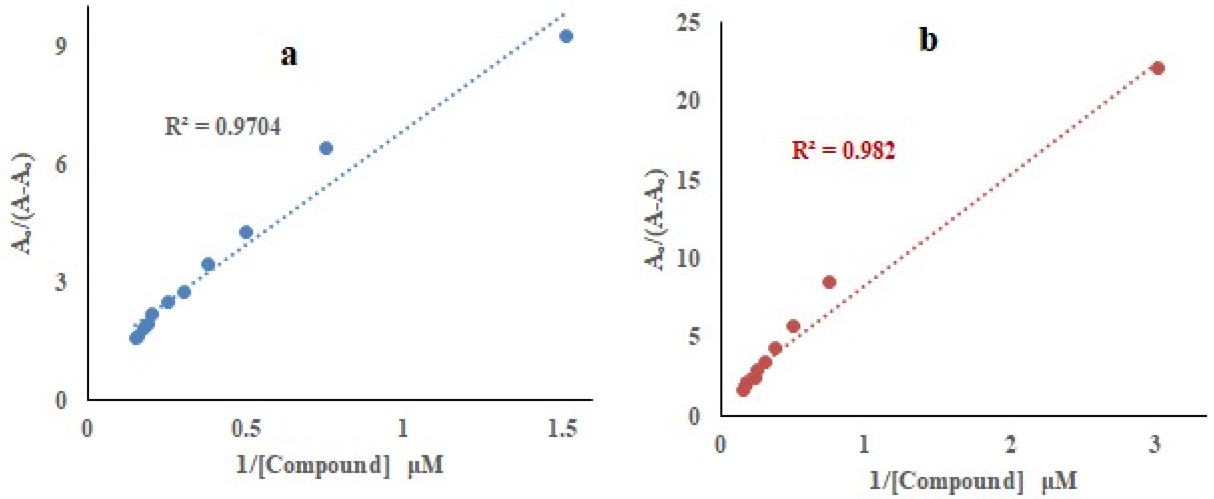

Figure S57. Benesi-Hildebrand plot {$A_o/(A-A_o)$ vs. 1/[compound]} of absorption spectra of HSA in the absence and presence of compound **12** (a) and compound **13** (b)

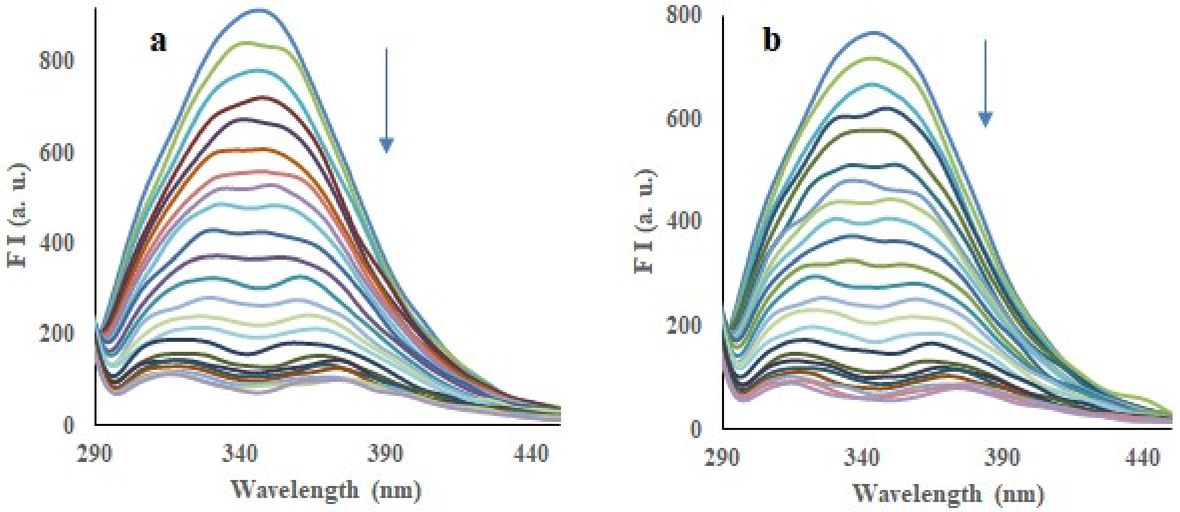

Figure S58. Emission spectra of HSA (10 μM) (λ_{ex} = 280 nm) in presence of increasing concentrations of compound **12** in phosphate buffer (*p*H 7.4) at 308 K (a) and 318 K (b)

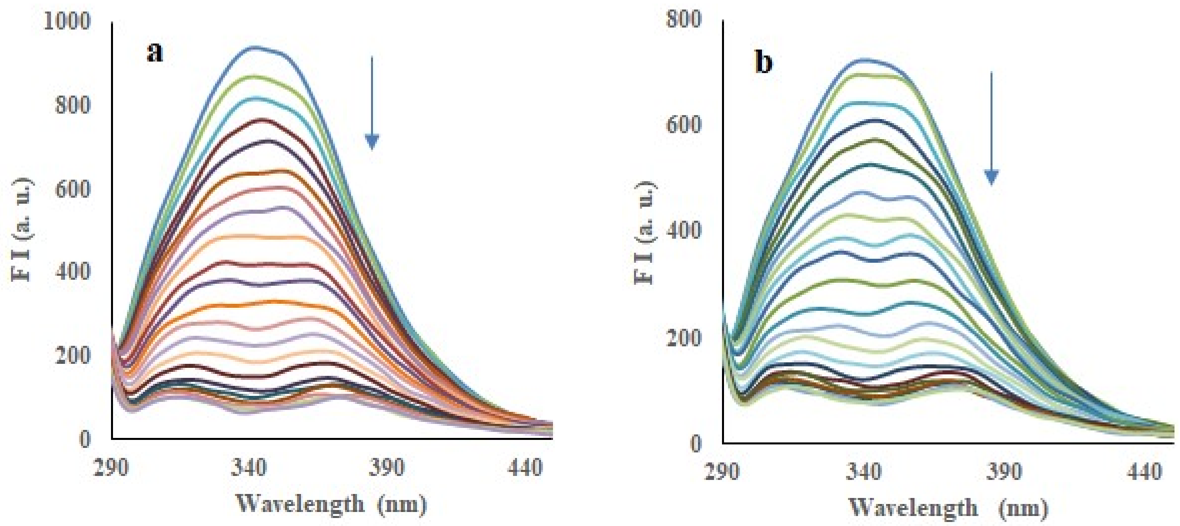

Figure S59. Emission spectra of HSA (10 μM) (λ_{ex} = 280 nm) in presence of increasing concentrations of compound **13** in phosphate buffer (*p*H 7.4) at 308 K (a) and 318 K (b)

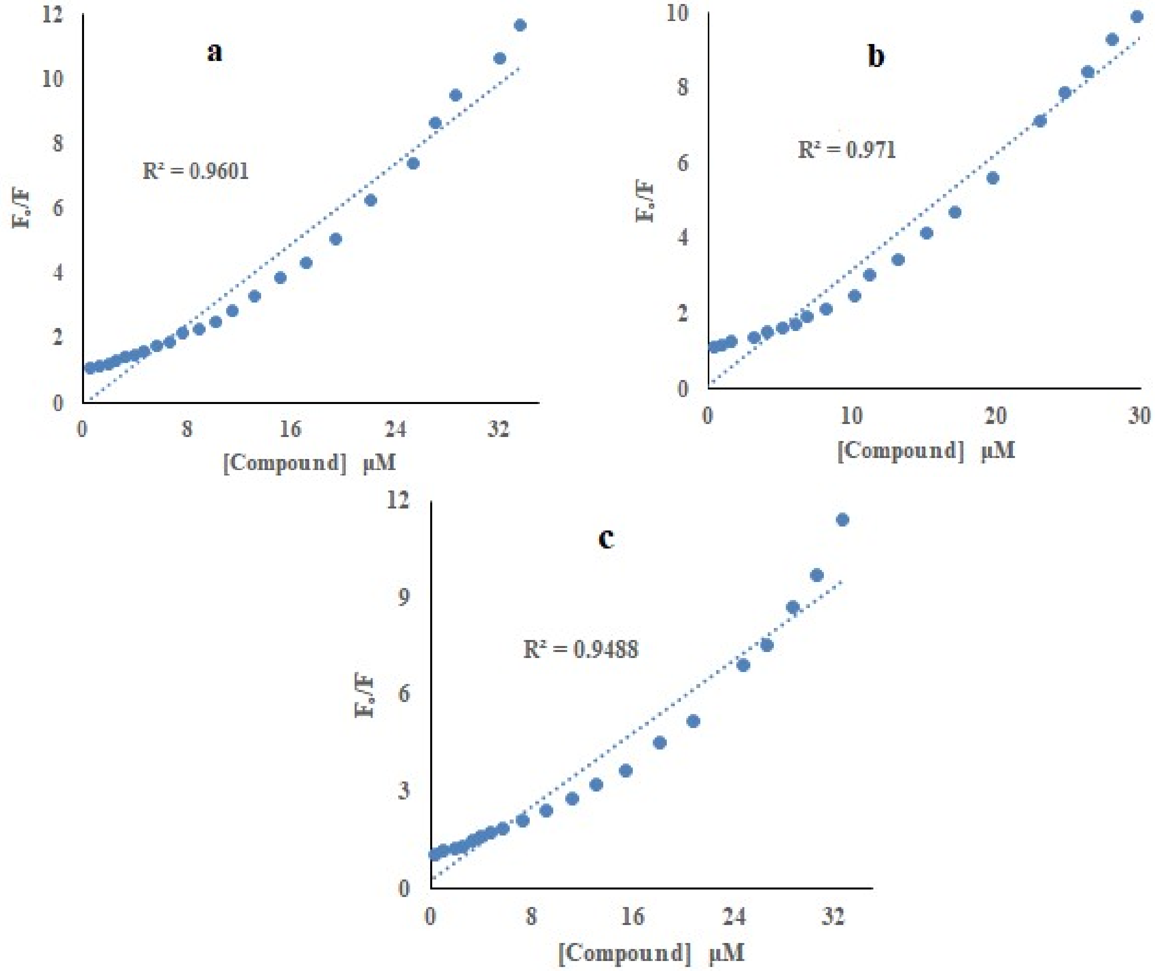

Figure S60. Stern-Volmer plots {F_0/F vs. [compound]} of emission spectra of HSA in the absence and presence of compound **12** at 298 K (a), 308 K (b) and 318 K (c)

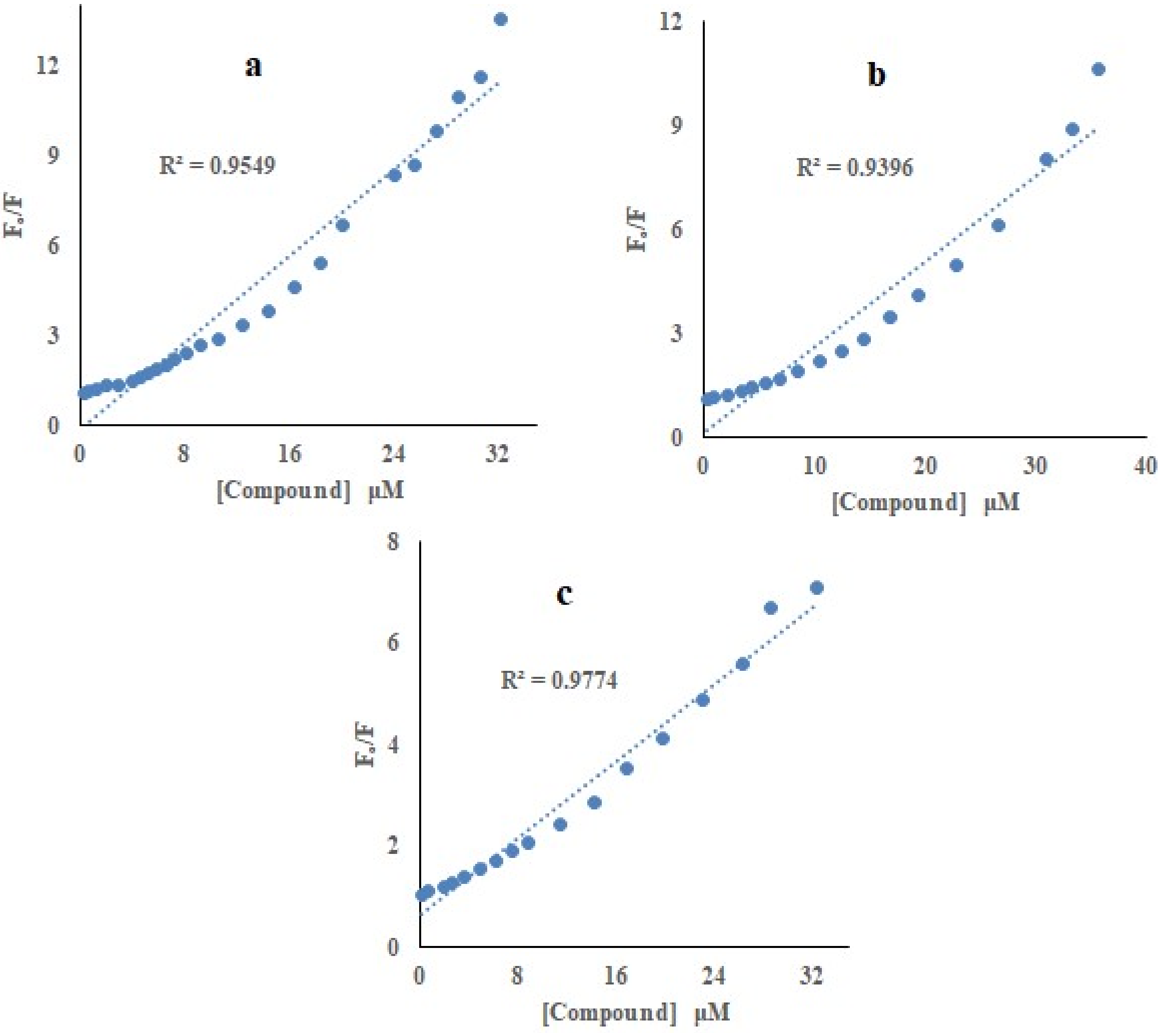

Figure S61. Stern-Volmer plots {F_0/F vs. [compound]} of emission spectra of HSA in the absence and presence of compound **13** at 298 K (a), 308 K (b) and 318 K (c)

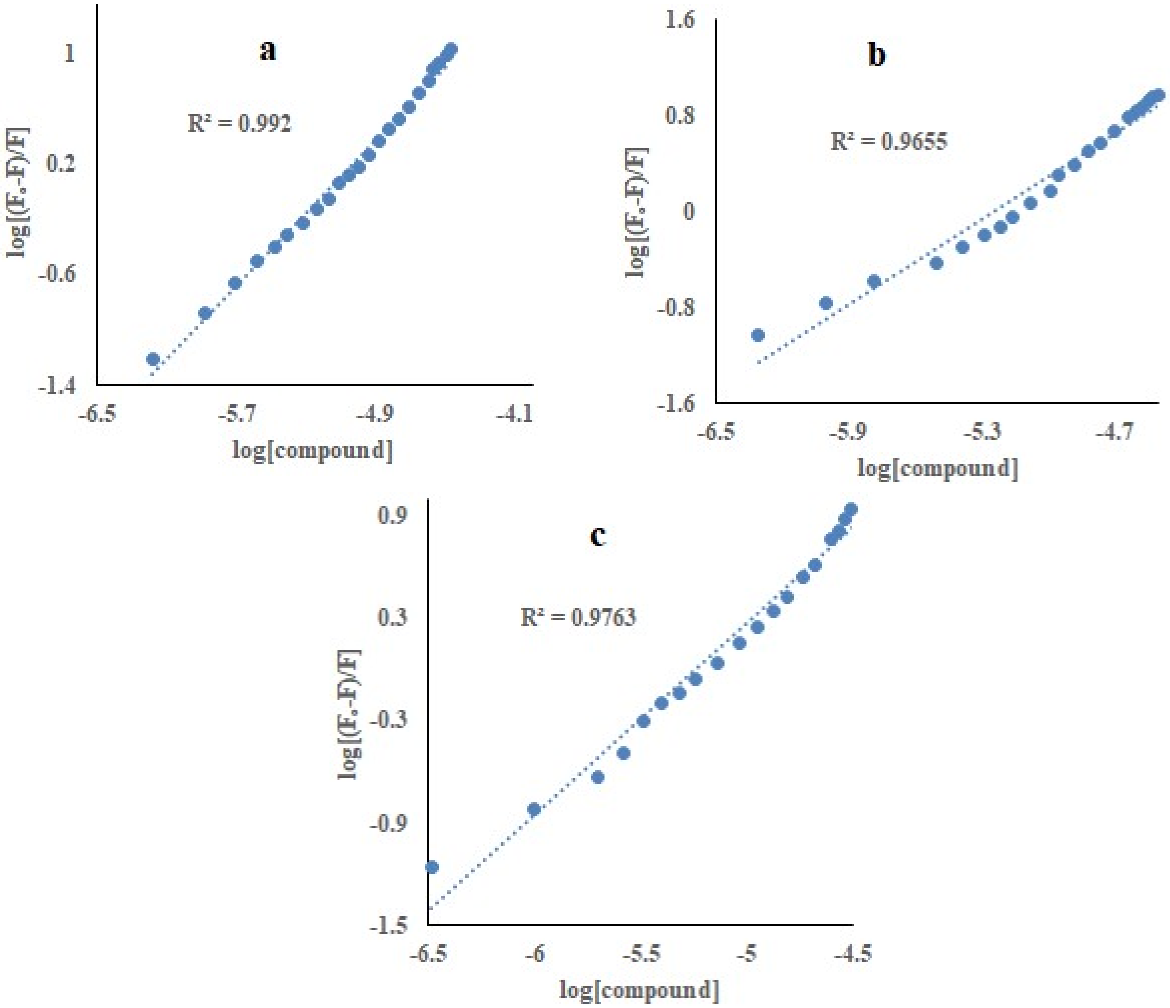

Figure S62. Modified Stern-Volmer plots {log [(F_0-F)/F] vs. log [compound]} of emission spectra of HSA in the absence and presence of compound **12** at 298 K (a), 308 K (b) and 318 K (c)

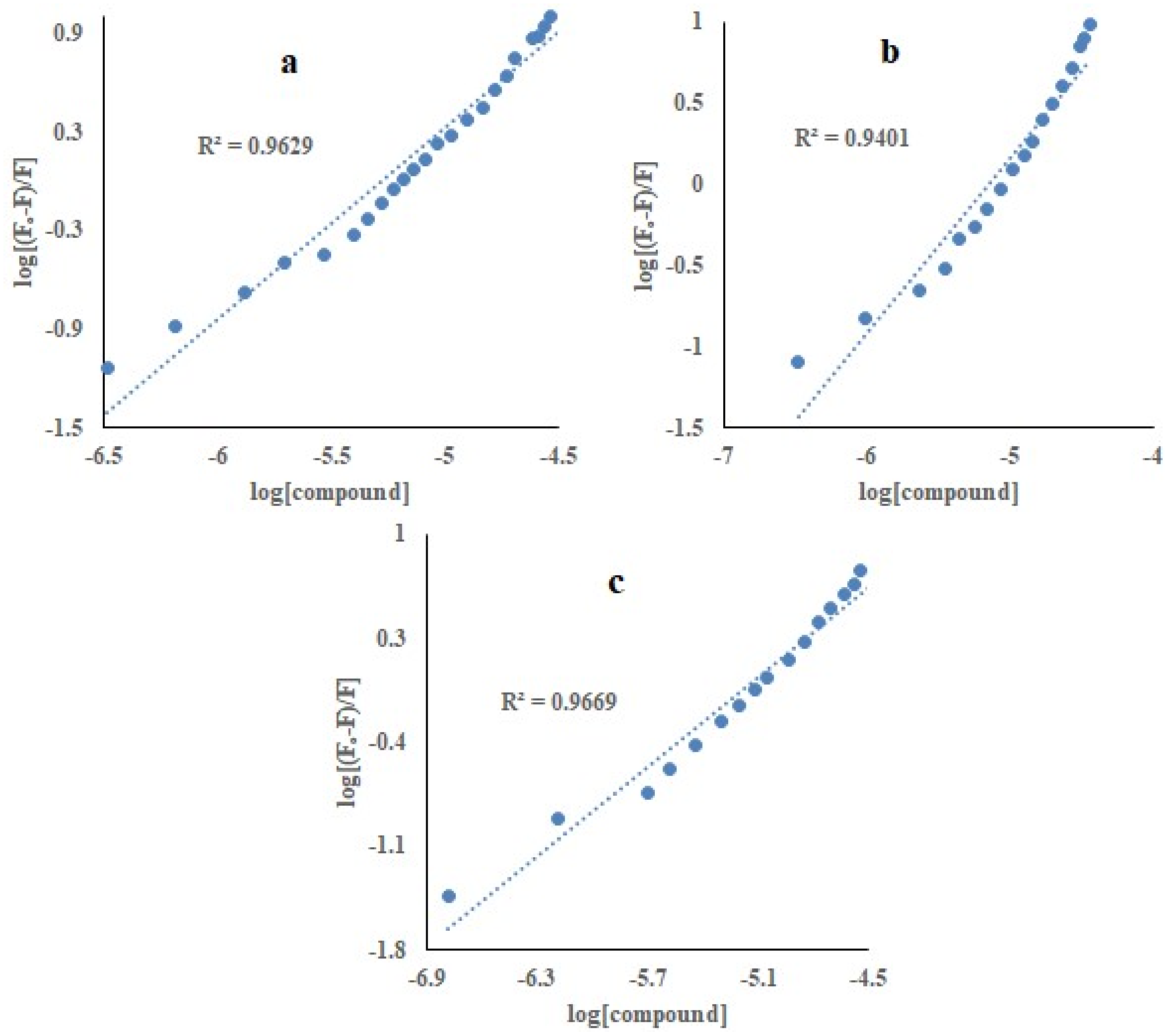

Figure S63. Modified Stern-Volmer plots {log [(F_0-F)/F] vs. log [compound]} of emission spectra of HSA in the absence and presence of compound **13** at 298 K (a), 308 K (b) and 318 K (c)

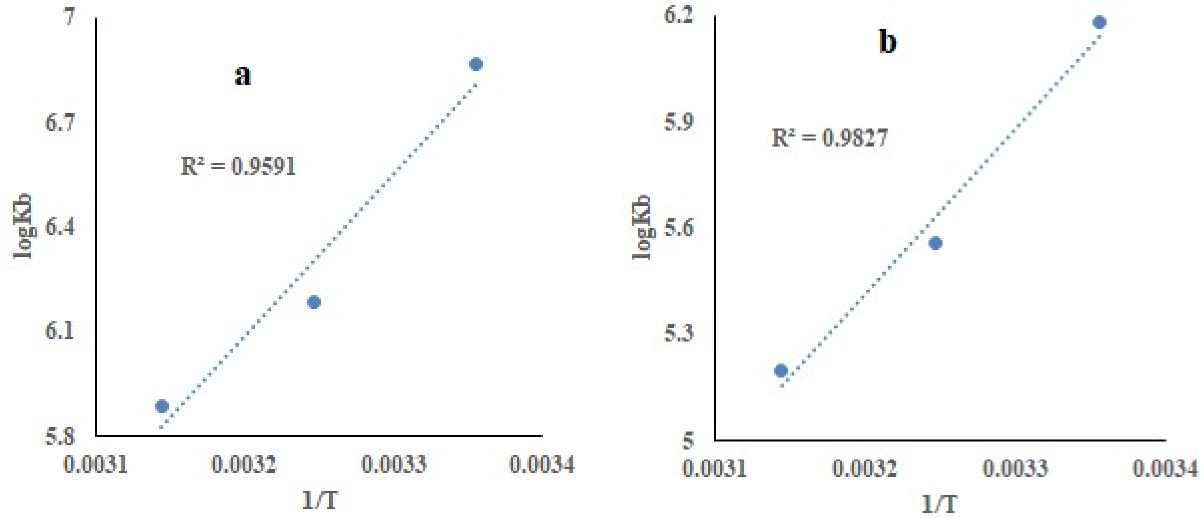

Figure S64. Van't Hoff plots {log K_b vs. 1/T} of emission spectra of HSA in the absence and presence of compound **12** (a) and compound **13** (b) at three different temperatures (298 K, 308 K and 318 K)

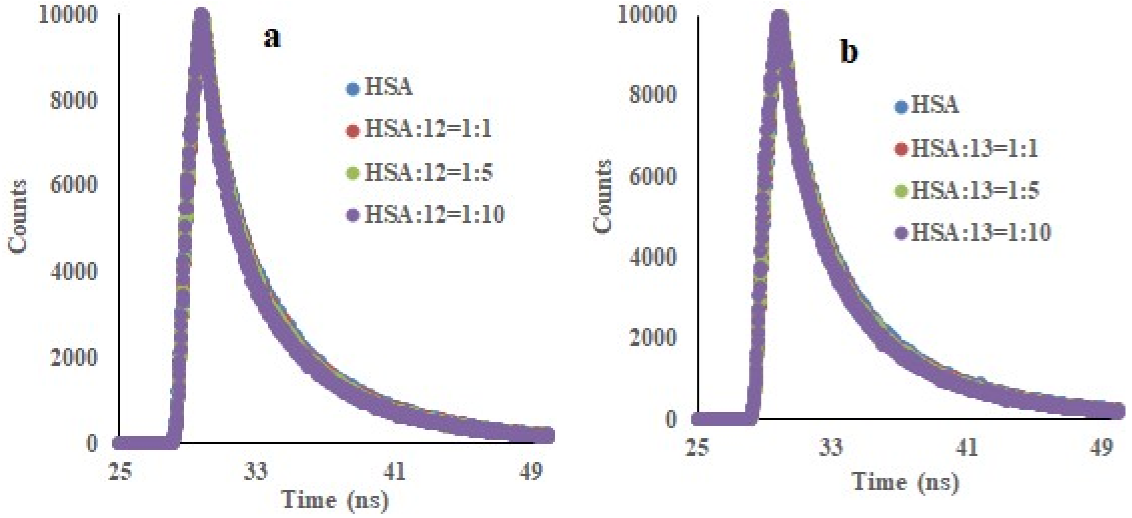

Figure S65. Fluorescence lifetime spectra of HSA in free form and the presence of compounds **12** (a) and **13** (b)

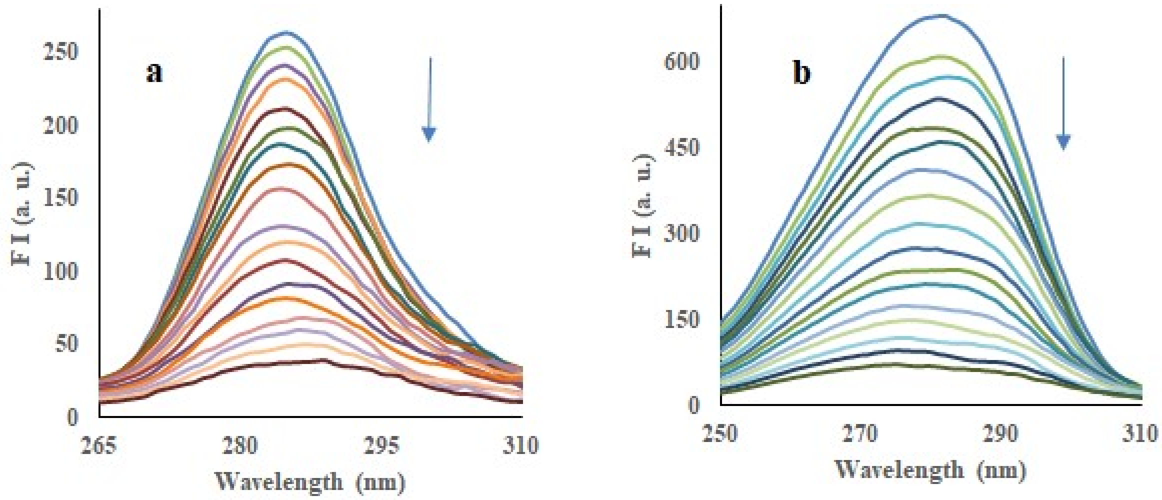

Figure S66. Synchronous fluorescence spectra of the HSA with increasing concentration of compound **13** using Δλ = 15 nm (a) and Δλ = 60 nm (b)

Table S1: The docking results based on the binding free energies (kcal/mol) of compounds **8-11** docked into 1ZXM and RMSD from the co-crystallized ligand

Mode	Compound 8		Compound 9		Compound 10		Compound 11	
	Affinity (kcal/mol)	RMSD (Å)	Affinity (kcal/mol)	RMSD (Å)	Affinity (kcal/mol)	RMSD (Å)	Affinity (kcal/mol)	RMSD (Å)
1	-10.5	0.000	-8.9	0.000	-8.1	0.000	-8.4	0.000
2	-10.3	3.700	-8.8	27.376	-8.1	14.138	-8.4	35.106
3	-9.4	13.964	-8.8	23.727	-7.7	14.371	-8.2	51.236
4	-9.4	39.779	-8.7	22.685	-7.4	15.323	-8.2	44.156
5	-9.4	19.027	-8.5	13.220	-7.4	18.185	-8.1	51.493
6	-9.4	15.661	-8.4	16.892	-7.4	42.265	-8.1	27.896
7	-8.9	40.360	-8.3	15.842	-7.4	45.203	-7.9	3.751
8	-8.9	9.746	-8.2	28.981	-7.2	34.006	-7.8	21.224
9	-8.8	39.715	-8.1	27.167	-7.2	33.641	-7.7	3.447

Table S2: The docking results based on the binding free energies (kcal/mol) of compounds **12-15** docked into 1ZXM and RMSD from the co-crystallized ligand

Mode	Compound 12		Compound 13		Compound 14		Compound 15	
	Affinity (kcal/mol)	RMSD (Å)	Affinity (kcal/mol)	RMSD (Å)	Affinity (kcal/mol)	RMSD (Å)	Affinity (kcal/mol)	RMSD (Å)
1	-10.7	0.000	-11.0	0.000	-10.1	0.000	-9.9	0.000
2	-10.5	21.980	-10.3	4.945	-9.9	4.532	8.4	4.345
3	-9.3	17.171	-10.0	8.868	-9.7	4.301	8.2	5.418
4	-9.3	18.866	-9.9	9.085	-9.6	15.277	8.1	14.247
5	-9.2	42.321	-9.9	9.185	-9.2	12.078	-7.9	10.880
6	-9.1	15.462	-9.8	9.647	-8.9	12.387	-7.6	40.227
7	-9.0	13.639	-9.8	17.299	-8.9	4.892	-7.	21.833
8	-8.7	12.118	-9.5	9.229	-8.8	36.393	-7.6	34.614
9	-8.7	34.149	-9.3	9.677	-8.7	12.558	-7.5	14.186

Table S3: The docking results based on the binding free energies (kcal/mol) of compounds **16-19** docked into 1ZXM and RMSD from the co-crystallized ligand

Mode	Compound 16		Compound 17		Compound 18		Compound 19	
	Affinity (kcal/mol)	RMSD (Å)	Affinity (kcal/mol)	RMSD (Å)	Affinity (kcal/mol)	RMSD (Å)	Affinity (kcal/mol)	RMSD (Å)
1	-9.1	0.000	-10.7	0.000	-10.4	0.000	-9.5	0.000
2	-8.9	3.532	-9.9	1.454	-10.4	4.066	-8.7	1.252
3	-8.7	21.972	-9.5	1.663	-10.2	36.770	-8.5	33.618
4	-8.7	5.184	-9.4	39.768	-10.1	36.700	-8.5	52.839
5	-8.5	13.130	-9.3	4.964	-9.9	11.192	-8.4	36.960
6	-8.5	3.147	-9.1	4.483	-9.6	9.370	-8.3	54.265
7	-8.4	7.289	-9.0	40.216	-9.6	11.971	-8.3	3.814
8	-8.4	32.823	-8.9	41.039	-9.5	3.604	-8.3	14.055
9	-8.4	35.526	-8.6	22.312	-9.5	23.684	-7.8	33.297

Table S4: The docking results based on the binding free energies (kcal/mol) of compounds **20-22** and etoposide docked into 1ZXM and RMSD from the co-crystallized ligand

Mode	Compound 20		Compound 21		Compound 22		Etoposide	
	Affinity (kcal/mol)	RMSD (Å)	Affinity (kcal/mol)	RMSD (Å)	Affinity (kcal/mol)	RMSD (Å)	Affinity (kcal/mol)	RMSD (Å)
1	-10.1	0.000	-10.2	0.000	-10.4	0.000	-11.2	0.000
2	-10.0	2.678	-10.0	16.868	-10.1	1.791	-10.8	4.577
3	-9.8	2.524	-9.7	4.024	-10.1	20.786	-10.6	2.206
4	-9.7	18.370	-9.5	27.913	-10.0	40.968	-9.8	12.535
5	-9.4	17.976	-9.5	16.263	-9.9	42.036	-9.6	12.619
6	-9.4	17.452	-9.4	16.542	-9.6	12.895	-9.3	45.489
7	-9.3	3.297	-9.4	30.416	-9.6	7.715	-9.3	24.083
8	-9.1	2.598	-9.3	19.851	-9.5	41.847	-9.3	20.267
9	-9.1	3.514	-9.2	7.819	-9.4	7.431	-9.3	34.636
